# Multidisciplinary home-based interventions in adverse events and quality of life among frail older people: A systematic review and meta-analysis

**DOI:** 10.1016/j.heliyon.2024.e40015

**Published:** 2024-10-31

**Authors:** Marta Carolina Ruiz-Grao, Celia Álvarez-Bueno, Miriam Garrido-Miguel, Carlos Berlanga-Macias, Marta Gonzalez-Molinero, Beatriz Rodríguez-Martín

**Affiliations:** a Nursing Faculty, University of Castilla-La Mancha, Albacete, Spain; b Health and Social Research Center, University of Castilla-La Mancha, Cuenca, Spain; c Nursing Faculty, University of Castilla-La Mancha, Cuenca, Spain; d Facultad de Ciencias de La Salud, Universidad Autónoma de Chile, Talca, Chile; e Faculty of Health Sciences, University of Castilla-La Mancha, Talavera de la Reina, Toledo, Spain

**Keywords:** Frail, Older people, Home-based interventions, Adverse events, Quality of life

## Abstract

**Objective:**

To determine the effects of multidisciplinary home-based interventions delivered by multidisciplinary teams to prevent adverse events (mortality, emergency visits, and hospital readmissions) and improve quality of life (QoL) among frail older adults.

**Methods:**

A systematic search of PubMed/MEDLINE, Scopus, the Cochrane Central Register of Controlled Trials, Web of Science, and the Cumulative Index to Nursing and Allied Health Literature was conducted until November 2023. The Risk of bias of the randomized controlled trials was assessed via the Cochrane risk of bias tool (RoB 2.0), and the synthesis and quality of evidence for each outcome were assessed via the Grading of Recommendation, Assessment, Development and Evaluation (GRADE). The effect size (ES), pooled OR (p-OR), and 95 % confidence intervals (95 % CIs) were calculated for QoL and adverse events. Subgroup analyses and meta-regressions were conducted.

**Results:**

Twenty-seven studies were included in the systematic review. The p-OR for the effect of home-based interventions on mortality was 0.88 (95 % CI: 0.75 to 1.02; I2 = 27.6 %), that for visits to the emergency department was 0.88 (95 % CI: 0.78 to 1.00; I2 = 56.4 %), that for hospital admissions in days was 0.85 (95 % CI: 0.52 to 1.37; I2 = 85.2 %), and that for hospital admissions was 0.90 (95 % CI: 0.79 to 1.01; I2 = 52.0 %). In addition, the pooled ES for the effect of home-based interventions on QoL was 0.08 (95 % CI: 0.02 to 0.17; I2 = 60.7 %). The type of intervention, type of control, evaluation of the risk of bias, duration of the intervention, mean age of the intervention group, and percentage of women in the intervention group did not modify the effect of MHBIs to prevent adverse events or to improve quality of life (QoL) among frail older adults.

**Conclusions:**

Multidisciplinary home-based interventions do not appear to reduce adverse events (mortality, visits to the emergency department, or hospital admissions/readmissions) in older people defined and/or considered frail. In addition, these interventions do not improve QoL in older people, or the evidence is unclear.

## Highlights

• Multidisciplinary home-based interventions do not appear to reduce adverse events nor improve QoL among frail older people.
• Data of this study should be carefully interpreted to inform policy makers to take actions and to reduce adverse events.
• Further research is needed to clarify the most appropriate multidisciplinary home-based interventions for frail older adults.

## List of abbreviations

Acute illness: (AI)
Admission avoidance: (AA)
Comprehensive geriatric assessment: (CGA)
Control group: (CG)
Chronic obstructive pulmonary disease: (COPD
Confidence Interval: (CI)
Quality of Life: (QoL)
Early supported discharge: (ESD)
Effect size: (ES)
Heart failure: (HF)
Making a post-acute illness recovery: (PAIR)
Multidisciplinary home-based interventions: (MHBIs)
Multiple geriatric conditions: (MGC)
National Health Services: (NHS)
Odds ratio: (OR)
Randomized controlled trial: (RCT)

## 1 Introduction

The concurrent increase in life expectancy and population ageing is associated with an increased risk of poor health, functional limitations, and frailty [1]. Frailty is defined as a progressive age-related decline in physiological systems that may result in decreased reserves of intrinsic capacity. This decline confers extreme vulnerability to stressors and increases the risk of a range of adverse health outcomes, including hospitalization, visits to emergency departments, falls, and mortality [[2], [3], [4]]. The prevalence of frailty among community-dwelling older adults is between 10.7 % and 12 % when physical frailty is used as a reference and close to 24 % when the deficit accumulation model is used, although these figures could vary by age and sex [1,5].

Frailty is associated with challenges in health systems and social services, including an increasing number of hospitalizations, visits to emergency departments, and mortality [[6], [7], [8], [9], [10]]. Therefore, interventions aimed at integrating different forms of health and social care in different settings could be necessary to mitigate frailty [11]. Integrated care includes multidisciplinary interventions that require the cooperation of multidisciplinary professional teams (including nurses, physicians, geriatricians, physiotherapists, social service physiotherapists, etc.) working together to deliver appropriate care in different settings [12]. A concept of interventions that permits older people to remain at home for as long as possible is being developed and validated [13,14]. This concept represents a comprehensive approach to address health and social care as part of a single and integrated system and is based on the interaction of three levels, i.e., person-centred care, place-based care, and the sustainability of care [14].

Home-based interventions based on hospitals could be defined as interventions that include active treatment at home for patients who otherwise would be admitted and receive care in the hospital. These interventions could contribute to increasing patient and carer satisfaction and safety and efficacy outcomes [[14], [15], [16]]. In addition, these interventions could reduce mortality rates, readmission rates, and costs [15,16]. In older adults, home-based hospital care could increase the time to readmission, improve quality of life (QoL), and reduce index costs among patients requiring hospital-level care with heart failure (HF) [17]. In addition, for frail older adults, case management could be effective in decreasing service use without increasing costs [18]. However, recent research has shown that interventions based on transitional care from hospital to home were effective in reducing the readmission rate [19], the residential care, and the health costs, and in improving satisfaction [20], in older adults but were inconclusive in improving mortality [20] or other health outcomes [19], including QoL, process-related outputs such as correct use of medication [14]. Unfortunately, previous systematic reviews and meta-analyses in the field have not been focused on randomized controlled trials (RCTs) including frail older adults as defined by the original studies; therefore, as far as we know, current evidence cannot be considered sufficiently strong for hospital-at-home interventions for frail older population.

The objective of our systematic review and meta-analysis was to determine the effectiveness of multidisciplinary home-based interventions (including case management, transitional care, home-based hospital services and mixed interventions) delivered by multidisciplinary teams to frail older people to prevent adverse events (mortality, visits to the emergency department, hospital admissions measured in days, and number of admissions) and improve the QoL of this population.

## 2 Methods

### 2.1 Search strategy and study selection

Our study was reported according to the Preferred Reporting Items for Systematic Reviews and Meta-Analyses (PRISMA) guidelines [21] (Supplemental Table S1) and carried out based on the recommendations of the Cochrane Manual for systematic reviews of interventions [22]. In addition, it was previously registered in PROSPERO (CRD42021291462).

The literature search was conducted using the PubMed/MEDLINE, Scopus, Cochrane Central Register of Controlled Trials, Web of Science, and Cumulative Index to Nursing and Allied Health Literature databases from their inception until November 2023.

The following terms were used in the search to link three different topics: i) home-based interventions (“home hospital”, “hospital at home”, “home-based hospitalization”, “home care services”, “hospital at home”, “patient facility”, “domiciliary care”, “home health care-delivered interventions”), ii) frailty (“frail elderly”, “elderly, frail”, “aged or elderly”, “aged 80 and over”, “centenarians”, “nonagenarians”, “octagenarians”, “functionally impaired elderly”), and iii) events (“involuntary hospitalization”, “involuntary treatment”, “avoidable hospitalization”, “patient admission”, “patient readmission”, “voluntary admission”, “patient readmission”, “30-day readmission”, “readmissions hospital”, “hospital readmissions”, “length of hospitalization”, “avoidable displacement from home”, “early supported discharge care”, “emergency department visit”, “visits to emergency department”, “emergency department admission”, “quality of life”, and “QoL”). Medical Subject Headings (MeSH) of the National Library of Medicine were used and combined to perform the PubMed search (Supplemental Table S2). Additionally, previous systematic reviews and meta-analyses and the references of the included studies were screened for potential studies.

#### 2.1.1 Eligibility criteria

We included studies [1] with a parallel or crossover randomized controlled trial (RCT) design [2]; including multidisciplinary home-based interventions; and [3] whose participants were diagnosed as frail older adults or older people who met frailty criteria.

Multidisciplinary home-based interventions (MHBIs) are considered interventions delivered by a multidisciplinary health team, defined as a formal team of two or more health professionals (nurses, physicians, geriatricians, physiotherapists, social service physiotherapists, etc.) working together to deliver health care. MHBIs are classified as follows: i) transitional care, defined as a set of actions designed to ensure the coordination and continuity of health care as patients transfer between locations (e.g., from the hospital to the patient's home, from primary care offices to the patient's home) or between different levels of care within the same location (e.g., from primary care to specialty care offices. In this study, we focused on transitional care between locations that included the patient's home and/or home care of older adults [23]. Additionally, MHBIs could include improved communication that contributes to safe transitional care through professional-oriented interventions (e.g., education), organizational/cultural interventions (e.g., transfer nurses and discharge protocols), or patient- and next-of-kin-oriented interventions (e.g., discharge support) [24]. ii) Case management is a targeted, community-based, and proactive approach that involves case finding, geriatric assessment, individualized care planning, and care coordination [25]. Case management is focused on ensuring the transition between services using different tools (e.g., comprehensive geriatric assessment) [26]. iii) Home-based hospital care includes the active treatment at home of patients who otherwise would be admitted and receive care in the hospital [14]. Home-based hospital care is a type of intervention closely related to transitional care [27]. Finally, iv) mixed interventions, for those cases in which interventions included more than one type of the abovementioned interventions or for which it is not possible to be included in one of the abovementioned interventions (e.g., home-based physical exercise).

Studies were excluded if [1] they included healthy patients within the intervention group [2]; the reported outcome variables were not related to avoid adverse events (mortality, emergency visits, hospital readmissions, and mortality) or improved QoL; or [3] they were written in a language other than English or Spanish.

### 2.2 Data extraction and quality assessment

An ad hoc table was used to extract the following data: first authors, year of publication, country, participants (sample size and control group (CG) size), demographic characteristics (age and % of female), health status of participants, potential principal stressful events common in the study population, classification/type of intervention (transitional care, case-management, hospital at home, or mixed interventions), length of intervention (days/months), team provider, and main outcomes (QoL, emergency visits, hospital readmissions, and mortality). Finally, information regarding the definition and/or consideration of frailty of the participants is summarized in a supplemental table.

The risk of bias of the individual RCTs was assessed via the Cochrane risk of bias tool for randomized trials (RoB 2.0) [28], which assesses the risk of bias according to 6 domains: randomization process, deviations from intended interventions, missing outcome data, measurement of the outcome, selection of reported results, and overall risk of bias.

The synthesis and quality of evidence for each outcome were analysed with the Grading of Recommendation, Assessment, Development and Evaluation (GRADE) approach [29]. The quality of the evidence was classified into four categories: high, moderate, low and very low [30]. (Supplemental Table S3).

Retrieval and selection of studies were performed in a stepwise manner. After duplicate records were removed, titles and abstracts were screened. Two reviewers assessed 15 % of the samples (MCR-G and CA-B). If their agreement reached 95 %, the first author continued the inclusion process alone. In the next step, two researchers (MCR-G and CA-B) independently read the full texts of the selected articles for the inclusion and exclusion criteria. In cases of doubt or disagreement, a third researcher (M G-M) was consulted.

Data extraction and quality assessment were independently performed by two researchers (i.e., MCR-G and CA-B).

### 2.3 Data statistical analysis

The pooled OR (p-OR) and 95 % CI were estimated for mortality, visits to the emergency department and hospital readmissions (measured in days of stay and number of admissions). In addition, the pooled standard mean difference (ES) and related 95 % confidence intervals (95 % CI) were estimated for patients’ QoL. When studies reported RRs, they were included in the analyses with ORs. The estimations were performed via the DerSimonian and Laird random effect methods. The inconsistency of the results across the studies was assessed via the *I*^*2*^ statistic, their corresponding P values, and the 95 % CI. *I*^*2*^ values from 0% to 30 % were considered “not important” inconsistencies, those from 30% to 50 % represented “moderate” inconsistencies, those from 50% to 75 % represented “substantial” inconsistencies, and those from 75% to 100 % represented “considerable” inconsistencies [31].

For each variable, subgroup analyses were performed based on the type of intervention, type of control, and risk of bias. Meta-regressions were conducted considering the duration of the intervention, the mean age of the intervention group, and the percentage of women in the intervention group. To determine whether any particular study could influence the results of the meta-analyses, we performed sensitivity analysis, where studies were individually removed from the pooled estimates. Finally, publication bias was assessed via Egger's regression asymmetry test. Statistical analyses were performed via StataSE software, version 15.

## 3 Results

### 3.1 Systematic review

#### 3.1.1 Study selection

**Fig. 1:**
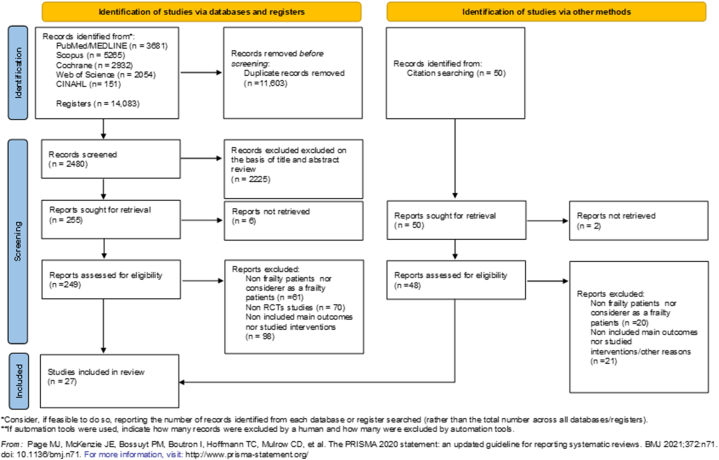
PRISMA 2020 flow diagram for new systematic reviews that included searches of databases, registers and other sources.

A total of 14,083 articles were identified. After screening the titles and abstracts, 249 full texts were reviewed, 27 of which were included in this systematic review (Fig. 1). All included studies were RCTs [[32], [33], [34], [35], [36], [37], [38], [39], [40], [41], [42], [43], [44], [45], [46], [47], [48], [49], [50], [51], [52], [53], [54], [55], [56], [57], [58]] and were published between 1998 and 2022. Table 1 summarizes the most relevant characteristics of the articles included in the systematic review (a list of excluded studies and reasons is provided in Supplemental Table S4).

**Table 1:** Preliminary study characteristics.

Reference	Country	Sample size	Age and %females	Health condition	Potential principal stressful event common in study's population,	Classification of intervention	Length of intervention (months)	Team provider	Main outcomes (time to follow-up/Quality of Life questionnaires used)
***Bernabei* et al.*, 1998***	Italy	200 (CG: 100)	≥65 70.5 %	MCNCD	MGC	CM	12	General practitioners, geriatrician, nurses, social workers.	Visits to emergency department (at 12 months follow-up) Mortality (at 12 months follow-up)
***Berntsen* et al.*, 2019***	Norway	1218 (CG: 779)	>60 65 %	MCNCD	MGC	CM	6–12	Nurse, physician, physiotherapist, occupational therapist, and pharmacist.	Visits to emergency department (at follow-up) Hospital admissions/readmissions (at 30 days and 12 months follow-up) Mortality (at 3- and 6-months follow-up)
***Bleijenberg* et al.*, 2016***	Netherlands	3092 (CG:856)	≥60 55.3%–56.0 %	MCND	MGC	MIXED	6–12	General practitioners, registered practice nurses	Visits to emergency department (at 12 months follow-up) Hospital admissions/readmissions (at 12 months follow-up) Mortality (at 12 months follow-up) Health Related Quality of Life (HRQL) (baseline, at 6- and 12-months follow-up): EuroQol-5D questionnaire
***Caplan* et al.*, 2006***	Australia	104 (CG: 34)	84–83.86 62.5 %	MCNCD	PAIR	HAH	6	Nurses, physiotherapists, occupational therapists, and doctors	Readmission to hospital within 28 days after end of rehabilitation Mortality (at 6 months follow-up)
***Casas-Herrero* et al.*, 2022***	Spain	188 (CG: 100)	>75 70.2 %	MCI	MGC	MIXED	3	Nurses, geriatrician, physiotherapists, and sport science graduate	Visits to emergency department (at 3 months follow-up) Hospital admissions/readmissions (at 3 months follow-up) Mortality (at 3 months follow-up) Health Related Quality of Life (HRQL) (at 3 months follow-up): EQ-VAS, Visual analogue scale of the EuroQol questionnaire
***Courtney* et al.*, 2009***	Australia	128 (CG:64)	≥65 62.3 %	MCNCD	AI	MIXED	6	Nurse and physiotherapist	Visits to emergency department (Baseline, 4-, 12- and 24-weeks follow-up) Hospital admissions/readmissions (Baseline, at 4-, 12- and 24-weeks follow-up) Mortality (at 24 weeks follow-up) HRQL (at 24 weeks follow-up): Medical Outcomes Study 12-item Short Form Survey (SF-12v2t)
***Dalby* et al.*, 2000***	Canada	142 (CG:69)	>70 67 %	MCNCD	MGC	CM	14	Nurse and primary care physician	Visits to emergency department (at 14 months follow-up) Hospital admissions/readmissions (overnight) (at 14 months follow-up14) Length of stay in hospital (at 14 months follow-up) Mortality (at 14 months follow-up)
***Ekdahl* et al.*, 2016***	Sweden	382 (CG:174)	≥75 49.5 %	MCNCD	MGC/PAIR	MIXED	36	Geriatric ambulatory team	Hospital admissions/readmissions (at 36 months follow-up) Length of hospital stay (at 36 months follow-up) Mortality (at 36 months follow-up) HRQL (baseline):
***Gagnon et aL.,l*1999**	Canada	427 (CG:215)	≥70 58 %	MCNCD	MGC/PAIR	CM	10	Nurse and physicians	Visits to emergency department (at 10 months follow-up) Hospital admissions/readmissions (at 10 months follow-up) Length of hospital stay (at 10 months follow-up) Mortality (at 10 months follow-up) HRQL (at 10 months follow-up): Medical Study Short Form (SF-36@
***Gilbert* et al.*, 2021***	France	705 (CG: 384)	≥75 63 %	AMED - MCNCD	PAIR	MIXED	1	Nurse	Visits to emergency department Hospital admissions/readmissions (at 3 months follow-up) Length of hospital stay Mortality (at 30 days follow-up) Quality of life (QL) (at 30 days follow-up): French version of EuroQoL-5D
***Jacobsohn* et al*, 2022***	USA	1756 (CG:893)	≥60 53 %	AMED	PAIR	TC	1	Community paramedic coaches	Visits to emergency department (at 14- and 30 days follow-up) HRQL (baseline): Medical Outcomes Study 12-item Short Form Survey (SF-12
***Jepma* et al*, 2021***	Netherlands	306 (CG:153)	≥70 49 %	CHF	PAIR	MIXED	6	Community nurse, Geriatricians, pharmacist and physical therapist	Hospital admissions/readmissions (at 3, 6 and 12 months follow-up) Mortality (at 3, 6 and 12 months follow-up)
***Lembeck* et al.*, 2019***	Denmark	537 (CG:267)	≥65 60 %	MCNCD	PAIR	CM	3	Nurse study, municipal nurses, and general practitioner	Hospital admissions/readmissions (at 8 days, 30 days and 3 months follow-up) Mortality (at 6 months follow-up)
***Leung* et al.*, 2004***	Hong Kong	92 (CG: 47)	≥65 53 %	MCNCD	MGC/PAIR	CM	12	Nurses trained in nursing older adults, geriatricians	Visits to emergency department (baseline and at 12 months follow-up) Hospital admissions/readmissions (baseline and at 12 months follow-up) Mortality (at 12 months follow-up)
***Liang* et al*, 2021***	Taiwan	200 (CG:100)	>65 58 %	MCNCD	MGC/PAIR	MIXED	6	Senior nurses, physicians, and technology engineers.	Visits to emergency department (6-months follow-up) Hospital admissions/readmissions (6-months follow-up) Mortality (6-months follow-up) HRQL (0,3 and 6 months follow-up): Chinese version of the EuroQol (EQ) scale (C-EQ5D-5L)
***Lindhardt* et al.*, 2019***	Denmark	330 (Group A: 117; Group B: 116; CG:116))	≥65 54 %	MCNCD	MGC/PAIR	CM	6	Nurse, general practitioner, and municipality preventive consultant	Hospital admissions/readmissions (at 3- and 6-months follow-up) Length of hospital stay (at 3- and 6-months follow-up) Mortality (at 6 months follow-up) Quality of life (at 3 months follow-up): EuroQol Five-item, Five-level (EQ-5D-5L) scale
***Melis* et al.*, 2008***	United Kingdom	151 (CG:66)	≥70 74.8 %	MCNCD - MCI	MGC	MIXED	3–6	Geriatric specialist nurse, primary care physicians, geriatricians	Mortality (at 6 and 12 months follow-up)
***Mogensen* et al.*, 2018***	Denmark	131 (CG:64)	≥65 64 %	AMED - MCNCD	AI	HAH	3	Municipally employed nurses, general practitioners, and physician's specialist in internal medicine	Hospital admissions/readmissions (at 7 days, 14 days, 21 days, 30 days and 90 days follow-up) Length of hospital stay (at 7 days and 90 days follow-up) Mortality (at 30 days and 90 days follow-up) Quality of life (at 7 days follow-up): self-completed EQ-5D instrument
***Parsons* et al.*, 2017***	New Zealand	113 (CG:57)	>65 61 %	MCNCD	MGC	MIXED	12	Multidisciplinary team (nurse case management)	Mortality (at 12 months follow-up) HRQL (12 months follow-up): 36-item Short-Form questionnaire (SF-36)
***Sahota* et al.*, 2017***	United Kingdom	250 (CG: 125)	≥70 64 %	AMED - MCNCD	AI/PAIR	TC	3	Senior occupational therapist, senior physiotherapist, assistant practitioner, and social services practitioner	Hospital admissions/readmissions (at 28- and 91-days follow-up) Length of hospital stay (at 28- and 91-days follow-up) Mortality (during follow-up) HRQL (91 days follow-up): EQ-5D- 3 L
***Sandberg* et al.*, 2015***	Sweden	153 (CG:73)	≥65 66.7 %	MCNCD	MGC	CM	12	Nurses, physicians, and physiotherapist	Visits to emergency department (at 6- and 12-months follow-up) Hospital admissions/readmissions (at 6- and 12-months follow-up) Length of hospital stay (at 6- and 12-months follow-up) Mortality (at 6- and 12-months follow-up)
***Schapira* et al.*, 2021***	Argentina	240 (CG: 120)	≥75 72.5 %	MCNCD	PAIR	MIXED	6	Nurses, geriatrician, physiotherapists, pharmacists, registered dietitians, speech therapists or social workers.	Visits to emergency department (at 6 months follow-up) Hospital admissions/readmissions (at days follow-up) Mortality (at 6 months follow-up)
***Senior* et al.*, 2014***	New Zealand	105 (CG:53)	≥65 54 %	MCNCD	MGC/PAIR	MIXED	24	Geriatricians and nurses	Mortality (at 24 months follow-up) HRQL (at 18 months follow-up): 36-item Short-Form questionnaire (SF-36)
***Shepperd* et al*, 2021***	United Kingdom	1055 (CG:355)	≥65 60.6 %	MCNCD	AI	MIXED	12	Geriatricians, nurse practitioners, physiotherapists and occupational therapists, social workers, mental health nurses and old age psychiatrists	Hospital admissions/readmissions (1 and 6 months follow-up) Mortality (6 and 12 months follow-up) HRQL (6 months follow-up): EQ5D-5L VAS
***Spoorenberg* et al.*, 2018***	Nethelands	1456 (CG: 709)	≥75 55 %	MCNCD	MGC	CM	12	General practitioners, nursing home physician, district nurses and social worker	Mortality (at 12 months follow-up)
***Suikkanen* et al.*, 2020***	Finland	299 (CG:149)	≥65 75 %	MCNCD	MGC	MIXED	12	Physiotherapist	Visits to emergency department (at 12- and 24-months follow-up) Hospital admissions/readmissions (at 12- and 24-months follow-up) Mortality (at 12- and 24-months follow-up) HRQL (at 12 months follow-up): HRQoL index 15 D questionnaire
***Thygesen* et al.*, 2015***	Denmark	531 (CG:261)	≥65 48 %	MCNCD	MGC/PAIR	MIXED	6	Municipal nurse and general practitioner	Visits to emergency department (at 1- and 6-months follow-up) Hospital admissions/readmissions (at 1- and 6-months follow-up) Mortality (at 1- and 6-months follow-up)

CG: control group; IG: intervention group; AI: acute illness; MGC: multiple geriatric conditions (dementia, immobility, incontinence, stroke deficits, etc.); PAIR: postacute illness recovery; AMED: acute medical emergency disease; MCNCD: multimorbidity chronic and nonchronic disease; CHF: cardiovascular heart failure; MCI: mid-level cognitive impairment.

#### 3.1.2 Study participants and intervention characteristics

A total of 14,291 participants classified as frail older adults or older people who met frailty criteria were included in the systematic review, of which 5843 belonged to the CG. Only six studies used established and validated criteria of fragility [34,40,46,48,56,58] (Supplemental Table S5). The included samples ranged from 92 [56] to 1756 [50] participants, with a mean age of 60 years or over, and included 48 % [49] to 75 % women [48] (see Table 1). Most of the included participants presented with multimorbidities and comorbidities. The participants presented with chronic health problems, including chronic HF, chronic obstructive pulmonary disease (COPD), stroke, hypertension, diabetes, and dementia. In addition, the main geriatric syndromes identified among these patients were falls, malnutrition, major cognitive disorders, disability, mobility impairment, or depression. The participants received interventions because of the presence of an acute medical condition (urinary infection, hip fracture, or gastrointestinal disease) or acute decompensation (Supplemental Table S6). The principal potential common stressor events in people living with frailty were an acute illness (AI) [35,42,53], multiple geriatric conditions (MGC) (including dementia, immobility, incontinence, and stroke deficits) [32,34,36,43,45,48,54,55,57,58], postacute illness recovery (PAIR) [33,39,40,46,50,51], and, in other cases, a combination of these conditions, such as AI/PAIR [42] or MGC/PAIR [37,38,41,47,49,52,56] (see Table 1).

The 27 interventions reported by the included studies were classified as transitional care (n = 2) [44,50], case-management services (n = 9) [31,36,38,40,41,45,54,56,58], interventions focused on home-based hospital care (n = 2) [34,42], and mixed interventions (n = 14) [34,35,37,39,43,[46], [47], [48], [49],[51], [52], [53],^55^,^57^]. The interventions were developed by multidisciplinary teams that included nurses, physicians, geriatricians, physiotherapists, social services practitioners, occupational therapists, or pharmacists. In a few cases, sports science graduates were included in the team [34,48].

The CGs performed conventional or routine care, including usual care based on continuing with their normal daily living activities and receiving habitual outpatient clinical care. This care included medical treatments and physical rehabilitation when needed [34], evidence-based care for emergency admission to the hospital [32], hospital specialist-based assistance [42], discharge letters from the hospital to the general practitioner [49], and nurses’ home visits and discharge planning [52].

All studies except 6 RCTs [40,43,47,49,57,58] included information for more than one outcome included in this meta-analysis. The main outcomes for this systematic review and meta-analysis were hospital readmissions reported in 22 RCTs [[32], [33], [34], [35], [36], [37], [38], [39], [40], [41], [42],[44], [45], [46],^48^,^49^,[51], [52], [53],^55^,^56^], lengths of hospital stay in 8 RCTs [[36], [37], [38], [39],^41^,^42^,^44^,^46^], emergency department admissions in 15 RCTs [32,[34], [35], [36],^38^,^39^,^45^,^46^,[48], [49], [50],^52^,[54], [55], [56]], and mortality in all but one included RCT [50]. In addition, QoL was reported in 15 RCTs [34,[36], [37], [38], [39],[41], [42], [43], [44],^47^,^48^,^50^,^52^,^53^,^55^].

#### 3.1.3 Quality assessment and risk of bias

As assessed by the RoB 2.0 tool, only three RCTs (11 %) were considered to have a low risk of bias [34,49,57], and three RCTs (11 %) were considered to have a high risk of bias [32,44,56], whereas the remaining RCTs presented “some concerns” (78 %).

When the risk of bias was analysed by individual domains, the randomization process was assessed as low risk in 74 % of the RCTs and high risk of bias in 3.7 % of the RCTs. Deviations from intended interventions were assessed as having a low risk of bias in 33.3 % of the RCTs, as having a high risk of bias in 11.1 % of the RCTs, and as having some concerns in the remaining RCTs. The domain of missing outcome data was assessed as having a low risk of bias in 81.5 % of the RCTs, and the remaining studies presented a high risk of bias (7.4 %) and some concerns (11.1 %). The domain of measurement of the outcome was assessed as low risk of bias in 40.7 % of the RCTs, and the remaining studies showed high risk of bias (40.7 %) and some concerns (18.6 %). Finally, the domain of the selection of reported studies was assessed as having a low risk of bias in 66.7 % of the RCTs, concerning 29.6 % of the studies, and as having a high risk of bias in 3.7 % of the RCTs (Supplemental Fig. S1).

### 3.2 Data synthesis

#### 3.2.1 Meta-analysis

##### 3.2.1.1 Mortality

**Fig. 2:**
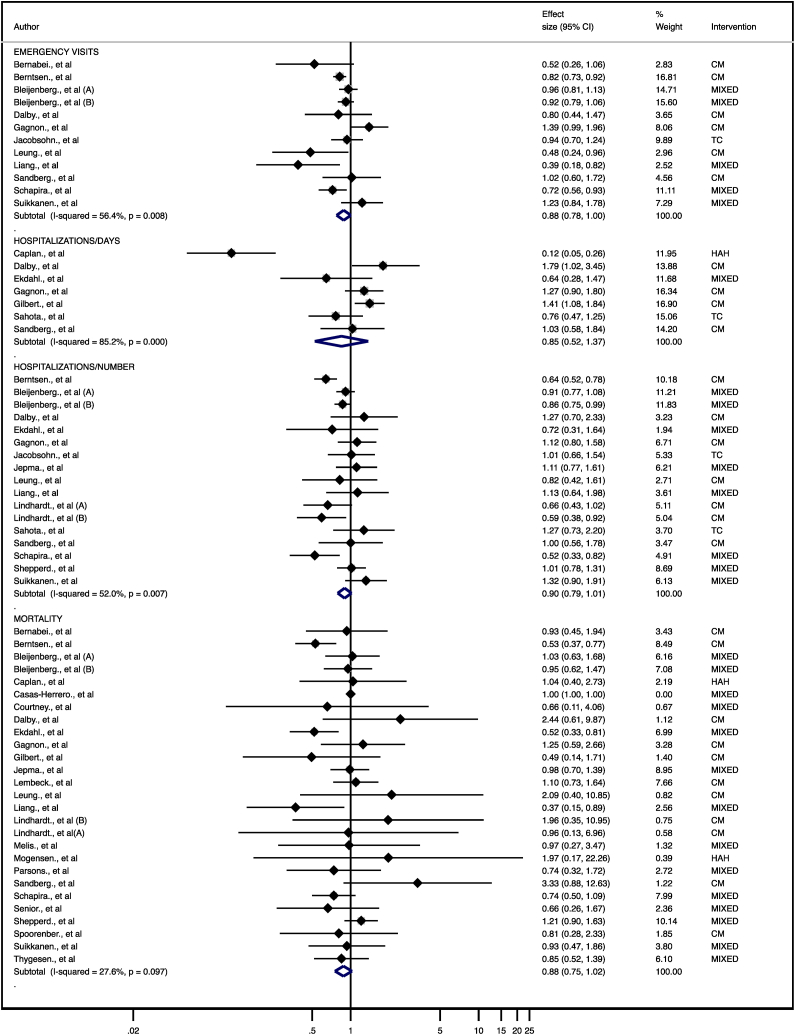
Forest plot of the effect size for several interventions in frail older people compared with control groups for emergency department visits (number), hospitalizations (days), hospitalizations (number) and mortality. CM: Case management; HAH: Hospital at home; TC: Transitional care; MIXED: included a combination of interventions or another type of intervention.

Twenty-seven studies included data to perform the meta-analysis for mortality. The p-OR estimate revealed no significant difference in mortality (0.88, 95 % CI (0.75–1.02)) between the MHBI group and the control group, with no serious inconsistency (*I*^*2*^ 27.6 %, p = 0.097) (Fig. 2). The quality of evidence according to the GRADE rating was moderate (Supplemental Table S3).

##### 3.2.1.2 Emergency visits

Twelve studies included data to perform the meta-analysis for emergency visits. The overall p-OR estimate revealed no significant difference for emergency visits (0.88, 95 % CI (0.78–1.00) in the MHBI group with respect to the control group, with moderate inconsistency (*I*^*2*^ 56.4 %, p = 0.008) (Fig. 2). The quality of evidence according to the GRADE rating was moderate (Supplemental Table S3).

##### 3.2.1.3 Hospitalization days

Seven studies included data to perform the meta-analysis for hospitalization days. The p-OR estimate revealed no significant difference for hospitalization days (0.85, 95 % CI (0.52–1.37) between the MHBI group and the control group, with serious inconsistency (*I*^*2*^ 85,2 % p = 0.000) (Fig. 2). The quality of evidence according to the GRADE rating was low (Supplemental Table S3).

##### 3.2.1.4 Number of hospitalizations

Seventeen studies included data to perform the meta-analysis for the number of hospitalizations. The p-OR estimate revealed no significant difference in the number of hospitalizations (0.90, 95 % CI (0.79–1.01)) between the MHBI group and the control group, with moderate/severe inconsistency (*I*^*2*^ 52,0 % p = 0.000) (Fig. 2). The quality of evidence according to the GRADE rating was low (Supplemental Table S3).

##### 3.2.1.5 Quality of life

**Fig. 3:**
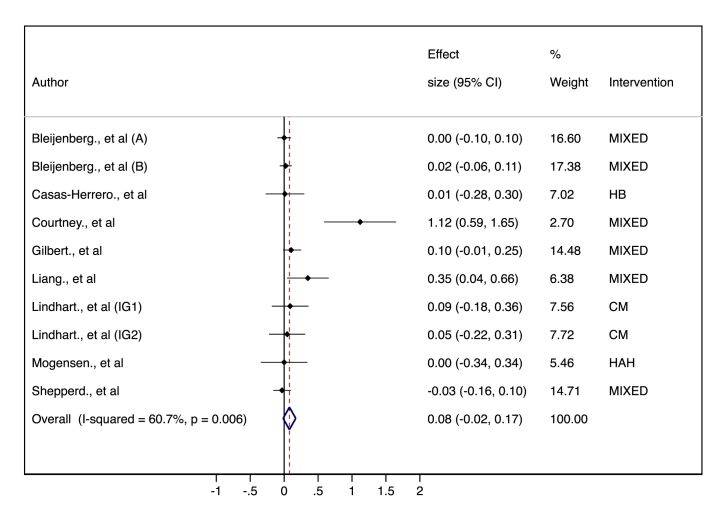
Forest plot of the effect sizes of several interventions on quality of life in frail older people compared with those in the control group. CM: Case management; HAH: Hospital at home; TC: Transitional care; MIXED: included a combination of interventions or another type of intervention.

Ten studies included data to perform the meta-analysis for quality of life. The included studies measured QoL via validated questionnaires: the EQ-VAS visual analogue scale of the EuroQol questionnaire [34], the 12-item Short Form Survey (SF-12v2t) [35], the French version of the EuroQoL-5D [39], the EuroQol Five-item Five-level (EQ-5D-5L) scale [41], the self-completed EQ-5D instrument [42], the Chinese version of the EuroQol (EQ) scale (C-EQ5D-5L) [52], the EuroQol-5D visual analogue scale (EQ5D-5L VAS) [53], and the EuroQol-5D questionnaire [55]. The p-OR estimate revealed no significant difference in quality of life (0.08, 95 % CI (−0.02 to 0.17)) between the MHBI group and the control group, with moderate inconsistency (*I*^*2*^ 60.7 %, p = 0.006) (Fig. 3). The quality of evidence according to the GRADE rating was moderate (Supplemental Table S3).

#### 3.2.2 Subgroup analyses and meta-regressions

Subgroup analyses revealed that the type of intervention, type of control, and evaluation of the risk of bias did not modify the pooled estimations for the effects of MHBIs on adverse events and QoL among frail older adults (Supplemental Table S7). Meta-regressions based on the duration of the intervention, mean age of the intervention group, and percentage of women in the intervention group were not associated with heterogeneity according to the random effects meta-regression models for the effect of MHBIs on preventing adverse events (mortality, emergency visits, and hospital readmissions) or improving quality of life (QoL) among frail older adults (Supplemental Table S8).

#### 3.2.3 Sensitivity analyses

After removing the individual studies from the analyses, p-OR was modified after removing the following samples: i) Gagnon et al. and Suikkanen et al. from the emergency estimations and ii) Gagnon et al. and Suikkanen et al. from the hospitalization in number estimations. Finally, we modified the mortality estimations (Supplemental Table S9).

### 3.2.4 Publication bias

Egger's test identified publication bias for the analyses of the days of hospitalization and mortality but not for QoL (Supplemental Table S10).

## 4 Discussion

This systematic review and meta-analysis was conducted to evaluate the ability of home-based interventions delivered by multidisciplinary teams to prevent adverse events, including mortality, visits to the emergency department, hospital admissions measured in days and number of admissions, among frail older people. Furthermore, improvements in QoL were studied in this population. The results showed that MHBIs did not reduce overall adverse events, including mortality, visits to the emergency department and hospital admissions measured as days and number of admissions, nor did they improve QoL among frail older people. Finally, the type of intervention, type of control, evaluation of the risk of bias, duration of the intervention, mean age of the intervention group, and percentage of women in the intervention group did not modify the effect of MHBIs to prevent adverse events (mortality, emergency visits, and hospital readmissions) or to improve quality of life (QoL) among frail older adults.

To our knowledge, this is the first systematic review and meta-analysis based on experimental studies to examine interventions focused on MHBIs to avoid adverse events and/or improve QoL in older adults considered frail. Previous systematic reviews have addressed the effectiveness of different outpatient care interventions to avoid and prevent adverse events in older people [20,24,[59], [60], [61]]. Interventions, including home care, may succeed in reducing hospital readmissions in older people, although the heterogeneity of interventions, measures, and methodologies impedes a robust level of evidence [20,60]. However, similar to our study, a systematic review focused on identifying potential interventions to develop effective and sustainable interventions to reduce avoidable displacement from home for older people with multimorbidity or frailty. This previous study has shown inconclusive evidence regarding the effectiveness of the interventions on hospital and emergency department admissions and readmissions [14,20]. Moreover, this evidence predominantly comes from quasiexperiments and observational studies that are not sufficiently robust to information policy and decision-making to reduce avoidable displacement from home [14].

Our data do not support that these interventions effectively improve mortality, visits to the emergency department, hospital admissions measured in days or the number of hospital admissions. Moreover, the evidence in this regard is mixed. Studies focused on the general population (aged >16 years) have shown that interventions based on home-based hospital care are associated with an increase in patient satisfaction and a reduction in adverse events, including mortality, readmission rates, and costs [16]. In addition, early supported discharge and home-based hospital care, compared with usual care in the hospital for patients with acute exacerbation of COPD, has been associated with lower rates of all-cause readmission and lower mortality [62,63]. A systematic review and meta-analysis focused on home-based hospital interventions for community-dwelling patients (median age: 71.0 years) with chronic diseases who presented to the emergency department and revealed that mortality did not differ between the home-based hospital and in-hospital care groups, similar to our study [64]. However, the risks of readmission and long-term care admission were lower in the home-based hospital intervention group than in the in-hospital care group [64]. Additionally, this study has the same limitations: our study focused on the high heterogeneity of some outcomes (hospital readmissions, mortality, etc.). This phenomenon was similar to those reported in other similar reviews [64]. However, in older adults with decompensated HF, home-based hospital care increased the time to readmission and improved the QoL compared with routine hospitalization but did not significantly reduce mortality or readmission [17]. These data are also reported for patients with stroke [65].

Our results show that MHBs do not improve the QoL of patients living with frailty. Some studies consider that subjective measures, such as QoL or process-related outputs, including the correct use of medication, should be evaluated instead of analysing typical outcomes, such as mortality or hospital admissions, but these variables are not reported by all studies [14]. However, when conducting the analyses for QoL, a trend of home-based interventions to improve QoL among frail older people was observed [35,41], with mixed and CM interventions being the intervention designs with better results.

Moreover, two main types of home-based hospital care have been recently identified: early supported discharge (ESD) and admission avoidance (AA). ESD is based on accelerating the discharge of admitted patients and partially displacing hospital care, whereas AA directly transfers patients to home-based hospital care from the emergency department or through general practitioner referrals, avoiding physical contact with the hospital [66]. The effects of ESD are similar to those of inpatient care [67], showing a trend towards higher readmissions for mixed conditions [66]. Furthermore, AA programs may lead to greater benefits in terms of satisfaction with health care received, clinical outcomes (mortality and hospital readmission rates) and costs when compared with inpatient care [20,59,66], but this service leads to no differences in 6-month mortality, hospital readmissions, or patient's self‐reported health status [20,68]. Similarly, our study is in line with a recent systematic review update [20] based on 20 RCTs whose main objective was to compare AA with acute hospital inpatient care, which revealed that, for an older population that included frail individuals, AA had little or no effect on mortality at the six-month follow‐up (risk ratio (RR) 0.88, 95 % confidence interval (CI) 0.68 to 1.13; P = 0.30; I2 = 0 %) and on the likelihood of being readmitted to the hospital after discharge from home-based hospital care or inpatient care within 3–12 months of follow‐up (RR 1.14, 95 % CI 0.971.34; P = 0.11; I2 = 41). In the same way, home-based hospital care resulted in little to no difference in patients' self‐reported health status, but satisfaction with health care received may be improved with AA [20]. The initial average length of hospital stay ranged from 4.1 to 18.5 days in the hospital group and from 1.2 to 5.1 days in the home-based hospital care group. AA likely decreases the likelihood of institutionalization and could reduce costs.

Currently, some National Health Services (NHSs) are developing virtual wards, defined as hospital-led alternatives to inpatient hospital care that may incorporate remote monitoring, such as apps, technology platforms, or other devices (i.e., wearable or pulse oximeters). Virtual wards have been observed to operate similarly to home-based hospital care for older people and could provide an alternative to inpatient care [69]. Nevertheless, not all NHSs are ready to adopt this model of care, and these services cannot be appropriated for the majority of older people [70]. Acute care-at-home services is a new intervention design that has been evaluated for older people with the aim of reducing hospital admissions by assessing and treating acutely unwell patients in their own home. It has been considered a viable alternative to hospitals for older patients, but this design suffers from higher mortality and readmission rates. These high rates could be due to the high proportion of frail patients who depend on care received by these services [71].

The interventions in this review include transitional care interventions for older adults that are focused primarily on care coordination and medication management and could impact health-related outcomes [71]. Among these interventions, the most commonly reported outcomes are beneficial in older populations, including a reduced incidence of readmission or hospitalization and improvements in mortality and QoL [72]. Moreover, recent research has shown heterogeneity in key components of these interventions, such as care continuity and coordination, medication management, symptom recognition, and care seeking [72]. Moreover, case management is a collaborative process focused on assessing, planning, facilitating, and advocating for services to meet an individual's holistic health needs, with the aim of managing older people's needs in the community and reducing access to acute health services [73]. The number of community-dwelling frail older people is increasing with the increasing number of very old people, and care management needs to be influenced by a salutogenic health care perspective and by a rehabilitative and family-oriented approach [74]. However, some studies have shown that community-based case management interventions may be effective in reducing visits to the emergency department, but not in the case of hospital admission in older people [73]. An integrative review focused on community nurse-led services for older people that included frail older adults did not provide clear evidence of a reduction in hospital admissions, mortality, emergency department visits or length of stay between the intervention and control groups [75]. However, this study concluded that self-help education and support for physical health problems among the older population in general and high-intensity team-based hospital-at-home services for specific diseases could be more effective at reducing adverse events (hospital admissions) than standard care or individual case management [75]. Furthermore, a recent review revealed that adapted physical exercise interventions could be the most appropriate type of intervention to address frailty in older adults [5]. This study suggested that these interventions may be carried out within more stable clinical settings, such as hospitals in home settings [5].

Finally, some of the interventions included in this study are based on the use of comprehensive geriatric assessment (CGA), which is a multidimensional interdisciplinary diagnostic process focused on determining an older person's medical, psychological and functional capability to develop a coordinated and integrated care plan. CGAs are not limited only to assessment; rather, they lead to tangible interventions [76]. However, a recent review revealed that CGAs had no impact on mortality in community-dwelling, frail, older people, and there is low-certainty evidence that in this population, those who undergo CGAs may have a reduced risk of unplanned hospital admission. In the case of the effect of the CGA on emergency department visits and/or QoL, the evidence is unclear, and standardized assessments are needed [76].

### 4.1 Study limitations

This systematic review and meta-analysis has several limitations. First, the terminology used in screening and managing frailty has recently been agreed upon in the field of geriatrics, and although the word “frail” has been used before, most interventional studies have focused on specific diseases rather than frailty. In addition, the population included in the studies was heterogeneous in terms of the diseases suffered. Second, this review is focused on outpatient and home-based interventions to avoid or reduce adverse events that are mainly conducted in home settings. We have not considered other settings, including those that may be applied in nursing homes, which should be the focus of future studies. Third, the interventions analysed differed in terms of health care providers and key coordinators involved, intervention setting, intervention components, follow-up duration, or measured outcomes, indicating that a specific intervention for frail older adults has not been clearly established. Fourth, the activities developed among the CG were heterogeneous and included receiving usual care, this care sometimes included a range of interventions. Fifth, the analysis revealed substantial levels of heterogeneity for several variables, and publication bias was observed for the number of hospitalizations and mortality variables; therefore, unpublished studies could modify our estimates. Sixth, all but one of the included studies were conducted in high-income countries. Seventh, an evidence gap exists regarding the interventions that target multidomain functions (e.g., psychosocial well-being or perceived health status); therefore, future studies should consider measuring outcomes that include subjective outcomes, such as self-efficacy, because it could be an important gap considering the increased confidence in managing their conditions/cope with frailty. Eighth, the risk of bias of the included studies could threaten the results. Finally, to our knowledge, this is the first systematic review and meta-analysis based on experimental studies to examine interventions focused on MHBIs to avoid adverse events and/or improve QoL in this population, which could explain the difficulty in contrasting our results with those of similar previously published studies.

## 5 Conclusions

The results of the present review and meta-analysis show that multidisciplinary home-based interventions do not reduce overall adverse events, including mortality, visits to the emergency department, or hospital admissions/readmissions, nor do they improve QoL among older frail patients. The ability of MHBIs to prevent adverse events (mortality, emergency visits, and hospital readmissions) or to improve quality of life (QoL) among frail older adults was not modified by the type of intervention, type of control, evaluation of the risk of bias, duration of the intervention, mean age of the intervention group, or percentage of women in the intervention group. However, the data of this review and meta-analysis should be interpreted carefully to help policy-makers reduce adverse events and avoid displacement from home in frail older people. Further studies are needed to clarify and investigate the most appropriate multidisciplinary home-based interventions or the combination of these interventions for frail older people in an integrated care setting.

## CRediT authorship contribution statement

**Marta Carolina Ruiz-Grao:** Writing – original draft, Methodology, Formal analysis, Conceptualization. **Celia Álvarez-Bueno:** Writing – review & editing, Methodology, Funding acquisition, Formal analysis, Conceptualization. **Miriam Garrido-Miguel:** Methodology, Formal analysis, Data curation. **Carlos Berlanga-Macias:** Writing – review & editing, Methodology, Formal analysis, Data curation. **Marta Gonzalez-Molinero:** Writing – review & editing, Methodology, Data curation. **Beatriz Rodríguez-Martín:** Writing – review & editing, Methodology, Conceptualization.

## Availability of data and materials

Data are available upon request. The data tables used to run the analyses are available upon request to the first author of this study.

## Funding sources

This research was funded by the 10.13039/501100007480University of Castilla-La Mancha cofunded by 10.13039/501100002924FEDER (Fondo Europeo de Desarrollo Regional) funds (Spain) (2022-GRIN-34426).

## Declaration of competing interest

The authors of this manuscript declare no conflict of interest.
