# Supplementary material for: Multidisciplinary home-based interventions in adverse events and quality of life among frail older people: A systematic review and meta-analysis
Source: Heliyon. 2024 Oct 31;10(21):e40015. doi: 10.1016/j.heliyon.2024.e40015 (PMC11582431; doi:10.1016/j.heliyon.2024.e40015)
Supplement: Multimedia component 1 [file mmc1.docx]

**Supplemental material:** Multidisciplinary home-based interventions in quality of life and adverse events among frail older people: a systematic review and meta-analysis

Content list:

- Tables:
- S1. PRISMA checklist
- S2. Search syntax for all databases
- S3. Summary of findings (SoF) and quality of evidence (GRADE) for multidisciplinary home-based interventions among frail older people
- S4. List of excluded studies and reasons
- S5. Frailty definition per study
- S6. Inclusion criteria, main health participants problems and intervention (experimental and control groups)
- S7. Subgroup analyses based on type of intervention, type of control, and evaluation of the risk of bias
- S8. Meta-regression according to duration of the intervention, mean age of the intervention group, and percentage of women in the intervention group
- S9. Sensitivity analyses
- S10. Publication bias
- Figures:
  - S1. Risk of bias of studies included as assessed with the RoB2 tool

**PRISMA checklist**

Table S1: PRISMA Checklist

| **Section and Topic** | **Item #** | **Checklist item** | **The location where the item is reported** |
| --- | --- | --- | --- |
| **TITLE** | | |  |
| Title | 1 | Identify the report as a systematic review. | Title page |
| **ABSTRACT** | | |  |
| Abstract | 2 | See the PRISMA 2020 for Abstracts checklist. | Page 1 |
| **INTRODUCTION** | | |  |
| Rationale | 3 | Describe the rationale for the review in the context of existing knowledge. | Page 2 |
| Objectives | 4 | Provide an explicit statement of the objective(s) or question(s) the review addresses. | Page 2 |
| **METHODS** | | |  |
| Eligibility criteria | 5 | Specify the inclusion and exclusion criteria for the review and how studies were grouped for the syntheses. | Page 3 & 4 |
| Information sources | 6 | Specify all databases, registers, websites, organisations, reference lists and other sources searched or consulted to identify studies. Specify the date when each source was last searched or consulted. | Page 3 |
| Search strategy | 7 | Present the full search strategies for all databases, registers and websites, including any filters and limits used. | Supplemental material |
| Selection process | 8 | Specify the methods used to decide whether a study met the inclusion criteria of the review, including how many reviewers screened each record and each report retrieved, whether they worked independently, and if applicable, details of automation tools used in the process. | Page 4 |
| Data collection process | 9 | Specify the methods used to collect data from reports, including how many reviewers collected data from each report, whether they worked independently, any processes for obtaining or confirming data from study investigators, and if applicable, details of automation tools used in the process. | Page 4 & 5 |
| Data items | 10a | List and define all outcomes for which data were sought. Specify whether all results that were compatible with each outcome domain in each study were sought (e.g. for all measures, time points, analyses), and if not, the methods used to decide which results to collect. | Page 5  Supplemental material |
|  | 10b | List and define all other variables for which data were sought (e.g. participant and intervention characteristics, funding sources). Describe any assumptions made about any missing or unclear information. | Page 5  Supplemental material |
| Study risk of bias assessment | 11 | Specify the methods used to assess the risk of bias in the included studies, including details of the tool(s) used, how many reviewers assessed each study and whether they worked independently, and if applicable, details of automation tools used in the process. | Page 5 |
| Effect measures | 12 | Specify for each outcome the effect measure(s) (e.g. risk ratio, mean difference) used in the synthesis or presentation of results. | Page 4 & 5 |
| Synthesis methods | 13a | Describe the processes used to decide which studies were eligible for each synthesis (e.g. tabulating the study intervention characteristics and comparing against the planned groups for each synthesis (item #5)). | Page 4 & 5 |
|  | 13b | Describe any methods required to prepare the data for presentation or synthesis, such as handling of missing summary statistics, or data conversions. | Page 4 & 5 |
|  | 13c | Describe any methods used to tabulate or visually display results of individual studies and syntheses. | Page 4 & 5 |
|  | 13d | Describe any methods used to synthesize results and provide a rationale for the choice(s). If meta-analysis was performed, describe the model(s), method(s) to identify the presence and extent of statistical heterogeneity, and software package(s) used. | Page 4 & 5 |
|  | 13e | Describe any methods used to explore possible causes of heterogeneity among study results (e.g. subgroup analysis, meta-regression). | Page 8 & 9 |
|  | 13f | Describe any sensitivity analyses conducted to assess robustness of the synthesized results. | Page 4, 5, 8 & 9 |
| Reporting bias assessment | 14 | Describe any methods used to assess risk of bias due to missing results in a synthesis (arising from reporting biases). | Page 5 |
| Certainty assessment | 15 | Describe any methods used to assess certainty (or confidence) in the body of evidence for an outcome. | Page 5 |
| **RESULTS** | | |  |
| Study selection | 16a | Describe the results of the search and selection process, from the number of records identified in the search to the number of studies included in the review, ideally using a flow diagram. | Figure 1 |
|  | 16b | Cite studies that might appear to meet the inclusion criteria, but which were excluded, and explain why they were excluded. | Not applicable. |
| Study characteristics | 17 | Cite each included study and present its characteristics. | Table 1 |
| Risk of bias in studies | 18 | Present assessments of risk of bias for each included study. | Supplemental material |
| Results of individual studies | 19 | For all outcomes, present, for each study: (a) summary statistics for each group (where appropriate) and (b) an effect estimate and its precision (e.g. confidence/credible interval), ideally using structured tables or plots. | Figures 2 and 3 |
| Results of syntheses | 20a | For each synthesis, briefly summarise the characteristics and risk of bias among contributing studies. | Several sentences page 6, 7, 8, 9 & supplemental Table S5 & S6 |
|  | 20b | Present results of all statistical syntheses conducted. If meta-analysis was done, present for each the summary estimate and its precision (e.g. confidence/credible interval) and measures of statistical heterogeneity. If comparing groups, describe the direction of the effect. | Figures 2 & 3 |
|  | 20c | Present results of all investigations of possible causes of heterogeneity among study results. | Page 8 & 9 |
|  | 20d | Present results of all sensitivity analyses conducted to assess the robustness of the synthesized results. | Supplemental material |
| Reporting biases | 21 | Present assessments of risk of bias due to missing results (arising from reporting biases) for each synthesis assessed. | Supplemental material |
| Certainty of evidence | 22 | Present assessments of certainty (or confidence) in the body of evidence for each outcome assessed. | Supplemental material and several places on Page 6, 7, 8 & 9 |
| **DISCUSSION** | | |  |
| Discussion | 23a | Provide a general interpretation of the results in the context of other evidence. | Page 8 & 9 |
|  | 23b | Discuss any limitations of the evidence included in the review. | Page 9,10, 11 & 12 |
|  | 23c | Discuss any limitations of the review processes used. | Page 12 |
|  | 23d | Discuss implications of the results for practice, policy, and future research. | Page 12 |
| **OTHER INFORMATION** | | |  |
| Registration and protocol | 24a | Provide registration information for the review, including register name and registration number, or state that the review was not registered. | Page 3 |
|  | 24b | Indicate where the review protocol can be accessed, or state that a protocol was not prepared. | Page 3 |
|  | 24c | Describe and explain any amendments to information provided at registration or in the protocol. | Not applicable |
| Support | 25 | Describe sources of financial or non-financial support for the review, and the role of the funders or sponsors in the review. | See funding and author contribution |
| Competing interests | 26 | Declare any competing interests of review authors. | see a conflict of interest |
| Availability of data, code and other materials | 27 | Report which of the following are publicly available and where they can be found: template data collection forms; data extracted from included studies; data used for all analyses; analytic code; any other materials used in the review. | Available on Figshare. See data availability. |

*From:*  Page MJ, McKenzie JE, Bossuyt PM, Boutron I, Hoffmann TC, Mulrow CD, et al. The PRISMA 2020 statement: an updated guideline for reporting systematic reviews. BMJ 2021;372:n71. doi: 10.1136/bmj.n71

For more information, visit: <http://www.prisma-statement.org/>

**Table S2.** Search syntax for all databases

| **Database** | **No** | **Search** |
| --- | --- | --- |
| **PubMed** | #1 | Search:((((((((((((("home"[Title/Abstract]) OR ("home hospital"[Title/Abstract])) OR ("home based hospitalization"[Title/Abstract])) OR ("out-patient facility"[Title/Abstract])) OR ("hospital in home"[Title/Abstract])) OR ("home care services, hospital based"[MeSH Terms])) OR ("Home care"[Title/Abstract])) OR ("Home-based primary care interventions"[All fields])) OR ("Hospital at home"[Title/Abstract])) OR ("Domiciliary care"[Title/Abstract])) OR ("outreach programme"[Title/Abstract])) OR ("hospital outreach"[Title/Abstract])) OR ("comprehensive elderly care"[Title/Abstract])) OR ("home hospitalisation"[Title/Abstract]) Filters: from 2000 – 2022 |
|  | #2 | ("frail elderly"[Title/Abstract] OR "frail elderly"[MeSH Terms] OR "elderly frail"[Title/Abstract] OR "Functionally-impaired Elderly"[Title/Abstract] OR "Aged OR elderly"[Title/Abstract] OR "aged 80 and over"[Title/Abstract] OR "aged, 80 and over"[MeSH Terms] OR "Centenarians"[Title/Abstract] OR "Nonagenarians"[Title/Abstract] OR "Octogenarians"[Title/Abstract]) Filters: from 2000 – 2022 |
|  | #3 | ("Involuntary hospitalisation"[Title/Abstract] OR "Involuntary Treatment"[MeSH Terms] OR "Avoidable hospitalisation"[Title/Abstract] OR "Patient admission"[Title/Abstract] OR "Patient Readmission"[MeSH Terms] OR "Voluntary Admission"[Title/Abstract] OR "Patient Readmission"[Title/Abstract] OR "30 Day Readmission"[Title/Abstract] OR "Thirty Day Readmission"[Title/Abstract] OR "readmissions hospital"[Title/Abstract] OR "Hospital Readmissions"[Title/Abstract] OR "Avoidable displacement from home"[Title/Abstract] OR "QoL"[Title/Abstract] OR "quality of life"[Title/Abstract]) Filters: from 2000 – 2022 |
|  | #4 | #1 AND #2 AND #3 |
| **Scopus** | #1 | TITLE-ABS ( "home"  OR  "home hospital"  OR  "home based hospitalization"  OR  "out-patient facility"  OR  "hospital in home"  OR  "Home Care Services, Hospital-Based"  OR  "Home care"  OR  "Home-based primary care interventions"  OR  "Hospital at home"  OR  "Domiciliary care"  OR  "outreach programme"  OR  "hospital outreach"  OR  "comprehensive elderly care"  OR  "home hospitalisation"  OR  "Home health care-delivered interventions" )  AND  PUBYEAR  >  2000 |
|  | #2 | TITLE-ABS ( "Frail elderly"  OR  "Elderly, Frail"  OR  "Functionally-impaired Elderly"  OR  "Aged OR elderly"  OR  "Aged, 80 and over"  OR  "Centenarians"  OR  "Nonagenarians"  OR  "Octogenarians" )  AND  PUBYEAR  >  2000 |
|  | #3 | TITLE-ABS ( "Involuntary hospitalisation" OR "Involuntary Treatment" OR "Avoidable hospitalisation" OR "Patient admission" OR "Patient Readmission" OR "Voluntary Admission" OR "Patient Readmission" OR "Early supported discharge care" OR "30 Day Readmission" OR "Thirty Day Readmission" OR "Hospital Readmissions" OR "Readmissions, Hospital" OR "Avoidable displacement from home" OR “QoL” OR “quality of life” ) AND PUBYEAR > 2000 |
|  | #4 | #1 AND #2 AND #3 |
| **Cochrane**  **Library** | #1 | "Hospital at home" |
|  | #2 | MeSH descriptor: [Home Care Services, Hospital-Based] explode all trees |
|  | #3 | MeSH descriptor: [Home Care Services] explode all trees |
|  | #4 | MeSH descriptor: [Hospitalization] explode all trees |
|  | #5 | ("domiciliary care"):ab,ti |
|  | #6 | ("home hospital"):ab,ti |
|  | #7 | ("outreach programme"):ab,ti |
|  | #8 | “("comprehensive elderly care"):ab,ti |
|  | #9 | ("home hospitalization"):ab,ti |
|  | #10 | MeSH descriptor:[Frail Elderly] explode all trees |
|  | #11 | (elderly frail):ab,ti |
|  | #12 | MeSH descriptor:[Aged, 80 and over] explode all trees |
|  | #13 | (centenarians):ab,ti |
|  | #14 | (nonagenarians):ab,ti |
|  | #15 | ("involuntary hospitalisation"):ab,ti |
|  | #16 | MeSH descriptor:[Involuntary treatment] explode all trees |
|  | #17 | ("avoidable hospitalisation2):ab,ti |
|  | #18 | ("patient admission"):ab,ti |
|  | #19 | MeSH descriptor:[Patient Readmission] explode all trees |
|  | #20 | ("voluntary Admission"):ab,ti |
|  | #21 | ("patient readmission"):ab,ti |
|  | #22 | ("hospital readmissions"):ab,ti |
|  | #23 | ("avoidable displacement from home"):ab,ti |
|  | #24 | ("QoL"):ab,ti |
|  | #24 | ("quality of life"):ab,ti |
|  |  | (#1 OR #2 OR #3 OR #4 OR #5 OR #6 OR #7 OR #8 OR #9) AND (#10 OR #11 OR #12 OR #13 OR #14) AND (#15 OR #16 OR #17 OR #18 OR #19 OR #20 OR #21 OR #22 OR #23 OR #24 OR #25) with Cochrane Library publication date from Jan 2000 to Nov 2023, in Cochrane Reviews |
| **Web of Science** | #1 | ts=(hospital* NEAR/3 home) |
|  | #2 | ts = "home hospital" |
|  | #3 | ts = "home based hospitalization" |
|  | #4 | ts = "out-patient facility" |
|  | #5 | ts = "Home Care Services, Hospital-Based" |
|  | #6 | ts= "Home care" |
|  | #7 | ts= "Home-based primary care interventions" |
|  | #8 | ts= "Hospital at home" |
|  | #9 | ts= "Domiciliary care" |
|  | #10 | ts= "outreach programme" |
|  | #11 | ts= "hospital outreach" |
|  | #12 | ts= "comprehensive elderly care" |
|  | #13 | ts= "home hospitalisation" |
|  | #14 | ts= "Home health care-delivered interventions" |
|  | #15 | ts= "Frail elderly" |
|  | #16 | ts= "Elderly, Frail" |
|  | #17 | ts= "Functionally-impaired Elderly" |
|  | #18 | ts= "Aged or elderly" |
|  | #19 | ts= "Aged, 80 and over" |
|  | #20 | ts= "Centenarians" |
|  | #21 | s= "Nonagenarians" |
|  | #22 | ts= "Octogenarians" |
|  | #23 | ts= "Involuntary hospitalisation" |
|  | #24 | ts= "Involuntary treatment" |
|  | #25 | ts="Avoidable hospitalisation" |
|  | #26 | ts= "Patient admission" |
|  | #27 | ts= "Patient readmission" |
|  | #28 | ts= "Early supported discharge care" |
|  | #29 | ts= "30 Day Readmission" |
|  | #30 | ts="Thirty Day Readmission" |
|  | #31 | ts= TS= "Hospital Readmissions" |
|  | #32 | ts= TS= "Readmissions, Hospital" |
|  | #33 | ts="Avoidable displacement from home" |
|  | #34 | ts="QoL" |
|  | #35 | ts="quality of life" |
|  | #34 | (#1 OR #2 OR #3 OR #4 OR #5 OR #6 OR #7 OR #8 OR #9 OR #10 OR #11 OR #12 OR #13 OR #14 ) AND (#15 OR #16 OR #17 OR #18 OR #19 OR #20 OR #21 OR #22 ) AND (#23 OR #24 OR #25 OR #26 OR #27 OR #28 OR #29 OR #30 OR #31 OR #32 OR #33 OR #34 OR #35) |
|  | #36 | ESCI Timespan=2000–2023 |
| **CINAHL** | #1 | "home hospital" |
|  | #2 | "home based hospitalization" |
|  | #3 | "out-patient facility" |
|  | #4 | "Home Care Services, Hospital-Based" |
|  | #5 | home care nurse |
|  | #6 | "Home care nurse" |
|  | #7 | Home-based primary care |
|  | #8 | "Hospital at home" |
|  | #9 | "Domiciliary care" |
|  | #10 | comprehensive elderly care |
|  | #11 | home hospitalization |
|  | #12 | "Frail elderly" |
|  | #13 | "Elderly, Frail" |
|  | #14 | Functionally-impaired Elderly |
|  | #15 | "Aged or elderly" |
|  | #16 | "Aged, 80 and over" |
|  | #17 | "Centenarians" |
|  | #18 | "Nonagenarians" |
|  | #19 | "Octogenarians" |
|  | #20 | "Involuntary hospitalisation" |
|  | #21 | "Involuntary treatment" |
|  | #22 | "Avoidable hospitalisation" |
|  | #23 | "Patient readmission" |
|  | #24 | Early supported discharge care |
|  | #25 | 30 Day Readmission |
|  | #26 | "Hospital Readmissions" |
|  | #27 | "QoL" |
|  | #28 | "quality of life" |
|  | #29 | (#1 OR #2 OR #3 OR #4 OR #5 OR #6 OR #7 OR #8 OR #9 OR #10 #11) AND (#12 OR #13 OR #14 OR #15 OR #16 OR #17 OR #18 OR #19) AND (#20 OR #21 OR #22 OR #23 OR #24 OR #25 OR #26 OR #27 OR #28) |
|  | #30 | Limits:abs;ti; AND(2000–2023)year  Only academic research |

**Table S3:** Summary of findings (SoF) and quality of evidence (GRADE) for multidisciplinary home-based interventions among frail older people

| **No. of studies** | **Study design** | **Risk of bias** | **Inconsistency** | **Indirectness** | **Imprecision** | **Outcome** | **Number patients**  **Multidisclinary home-based interventions** | **Number of patients Control intervention** | **Standard mean difference (95% CI)** | **Quality of evidence (GRADE)** | **Importance** |
| --- | --- | --- | --- | --- | --- | --- | --- | --- | --- | --- | --- |
| 27 | RCT | Serious | Modetate | Not serious | Moderate | Mortality | 6939 | 5346 | OR 0.88 (0.75 to 1.02) | Low | Important |
| 12 | RCT | Very serious | Moderate | Not serious | Serious | Emergency visits | 4418 | 3401 | OR 0.88 (0.78 to 1.00) | Moderate | Important |
| 7 | RCT | Serious | Serious | Not serious | Serious | Hospitalizations days | 1089 | 1074 | OR 0.85 (0.52 to 1.37) | Low | Important |
| 17 | RCT | Very serious | Moderate | Not serious | Serious | Hospitalizations number | 5737 | 4205 | OR 0.90 (0.79 to 1.01) | Low | Important |
| 10 | RCT | Serious | Moderate | Serious | Not Serious | Quality of life | 3809 | 2020 | OR 0.08 (−0.02 to 0.17) | Moderate | Important |

Abbreviations: CI, Confidence interval; RCT, Randomized clinical trial; OR, Odds Ratio

Quality of evidence. High: The research provides a very good indication of the likely effect. The probability that the effect is different is low Moderate: The research provides a good indication of the likely effect. The probability that the effect is substantially different is Moderate. Low: The research gives some indication of the probable effect. However, the probability that the effect is substantially different is high. Very low: The research does not provide a reliable estimate of the probable effect. The probability that the effect is substantially different is very high. Inconsistency: Probability of having different results in the studies included in the meta-analysis. This is assessed with the *I*^2^ test. Importance: The importance of the outcomes are determined by the author and regarding to the research question. They are weighted according to their importance for clinical decision making.

**Table S4.** List of excluded studies and reasons (in screening)

| **Authors** | **Study title** | **Exclusion reason** |
| --- | --- | --- |
| Reistetter, Timothy A.; Eschbach, Karl; Prochaska, John; Jupiter, Daniel C.; Hong, Ickpyo; Haas, Allen M.; Ottenbacher, Kenneth J. | Understanding Variation in Postacute Care Developing Rehabilitation Service Areas Through Geographic Mapping | Non frailty patients nor considerer as a frailty patients |
| Bowles, Kathryn H.; McDonald, Margaret; Barron, Yolanda; Kennedy, Erin; O'Connor, Melissa; Mikkelsen, Mark | Surviving COVID-19 After Hospital Discharge: Symptom, Functional, and Adverse Outcomes of Home Health Recipients | Non frailty patients nor considerer as a frailty patients |
| Konetzka, R. Tamara; Jung, Daniel H.; Gorges, Rebecca J.; Sanghavi, Prachi | Outcomes of Medicaid home- and community-based long-term services relative to nursing home care among dual eligibles | Non frailty patients nor considerer as a frailty patients |
| Fabius, Chanee; Shugrue, Noreen; Robison, Julie T. | Outcomes Associated with Home and Community-based Service Use Among Older Adults following a Nursing Home Transition | Non frailty patients nor considerer as a frailty patients |
| Glans, Maria; Ekstam, Annika Kragh; Jakobsson, Ulf; Bondesson, Asa; Midlov, Patrik | Risk factors for hospital readmission in older adults within 30days of discharge - a comparative retrospective study | Non frailty patients nor considerer as a frailty patients |
| Sorensen, Andrea; Grotts, Jonathan F.; Tseng, Chi-Hong; Moreno, Gerardo; Maranon, Richard; Whitmire, Natalie; Viramontes, Omar; Atkins, Sandy; Sefilyan, Ester; Simmons, June W.; Mangione, Carol M. | A Collaboration Among Primary Care-Based Clinical Pharmacists and Community-Based Health Coaches | Non frailty patients nor considerer as a frailty patients |
| Samaranayake, Chinthaka B.; Neill, Jane; Bint, Michael | Respiratory acute discharge service: a hospital in the home programme for chronic obstructive pulmonary disease exacerbations (RADS study) | Non frailty patients nor considerer as a frailty patients |
| Hoang, Chau M.; Davids, Jennifer S.; Maykel, Justin A.; Flahive, Julie M.; Sturrock, Paul R.; Alavi, Karim | Not All Discharge Settings Are Created Equal: Thirty-Day Readmission Risk after Elective Colorectal Surgery | Non frailty patients nor considerer as a frailty patients |
| Hong, Ickpyo; Knox, Sara; Pryor, Loree; Mroz, Tracy M.; Graham, James E.; Shields, Meredith F.; Reistetter, Timothy A. | Is Referral to Home Health Rehabilitation After Inpatient Rehabilitation Facility Associated With 90-Day Hospital Readmission for Adult Patients With Stroke? | Non frailty patients nor considerer as a frailty patients |
| Keim, Susan K.; Ratcliffe, Sarah J.; Naylor, Mary D.; Bowles, Kathryn H. | Patient Factors Linked with Return Acute Healthcare Use in Older Adults by Discharge Disposition | Non frailty patients nor considerer as a frailty patients |
| Knox, Sara; Downer, Brian; Haas, Allen; Middleton, Addie; Ottenbacher, Kenneth J. | Function and Caregiver Support Associated With Readmissions During Home Health for Individuals With Dementia | Non frailty patients nor considerer as a frailty patients |
| Coym, Anja; Ullrich, Anneke; Hackspiel, Lisa Kathrin; Ahrenholz, Mareike; Bokemeyer, Carsten; Oechsle, Karin | Systematic symptom and problem assessment at admission to the palliative care ward - perspectives and prognostic impacts | Non frailty patients nor considerer as a frailty patients |
| Augustine, Matthew R.; Davenport, Claire; Ornstein, Katherine A.; Cuan, Mitchell; Saenger, Pamela; Lubetsky, Sara; Federman, Alex; DeCherrie, Linda V.; Leff, Bruce; Siu, Albert L. | Implementation of Post-Acute Rehabilitation at Home: A Skilled Nursing Facility-Substitutive Model | Non frailty patients nor considerer as a frailty patients |
| Huyer, Gregory; Brown, Catherine R. L.; Spruin, Sarah; Hsu, Amy T.; Fisher, Stacey; Manuel, Douglas G.; Bronskill, Susan E.; Qureshi, Danial; Tanuseputro, Peter | Five-year risk of admission to long-term care home and death for older adults given a new diagnosis of dementia: a population-based retrospective cohort study | Non frailty patients nor considerer as a frailty patients |
| **Authors** | **Study title** | **Exclusion reason** |
| Mitchell, Michael A.; Dhaliwal, Inderdeep; Mulpuru, Sunita; Amjadi, Kayvan; Chee, Alex | Early Readmission to Hospital in Patients With Cancer With Malignant Pleural Effusions Analysis of the Nationwide Readmissions Database | Non frailty patients nor considerer as a frailty patients |
| Su, Mei-Chin; Wang, Yi-Jen; Chen, Tzeng-Ji; Chiu, Shiao-Hui; Chang, Hsiao-Ting; Huang, Mei-Shu; Hu, Li-Hui; Li, Chu-Chuan; Yang, Su-Ju; Wu, Jau-Ching; Chen, Yu-Chun | Assess the Performance and Cost-Effectiveness of LACE and HOSPITAL Re-Admission Prediction Models as a Risk Management Tool for Home Care Patients: An Evaluation Study of a Medical Center Affiliated Home Care Unit in Taiwan | Non frailty patients nor considerer as a frailty patients |
| Leavitt, Mary Ann; Hain, Debra J.; Keller, Kathryn B.; Newman, David | Testing the Effect of a Home Health Heart Failure Intervention on Hospital Readmissions, Heart Failure Knowledge, Self-Care, and Quality of Life | Non frailty patients nor considerer as a frailty patients |
| Levine, David M.; Ouchi, Kei; Blanchfield, Bonnie; Saenz, Agustina; Burke, Kimberly; Paz, Mary; Diamond, Keren; Pu, Charles T.; Schnipper, Jeffrey L. | Hospital-Level Care at Home for Acutely Ill Adults A Randomized Controlled Trial | Non frailty patients nor considerer as a frailty patients |
| Kosar, Cyrus M.; Loomer, Lacey; Ferdows, Nasim B.; Trivedi, Amal N.; Panagiotou, Orestis A.; Rahman, Momotazur | Assessment of Rural-Urban Differences in Postacute Care Utilization and Outcomes Among Older US Adults | Non frailty patients nor considerer as a frailty patients |
| Bowles, Kathryn H.; Murtaugh, Christopher M.; Jordan, Lizeyka; Barron, Yolanda; Mikkelsen, Mark E.; Whitehouse, Christina R.; Chase, Jo-Ana D.; Ryvicker, Miriam; Feldman, Penny Hollander | Sepsis Survivors Transitioned to Home Health Care: Characteristics and Early Readmission Risk Factors | Non frailty patients nor considerer as a frailty patients |
| Jones, Christine D.; Falvey, Jason; Hess, Edward; Levy, Cari R.; Nuccio, Eugene; Baron, Anna E.; Masoudi, Frederick A.; Stevens-Lapsley, Jennifer | Predicting Hospital Readmissions from Home Healthcare in Medicare Beneficiaries | Non frailty patients nor considerer as a frailty patients |
| Schluter, Philip J.; McAuliffe, Megan J.; Askew, Deborah A.; Jamieson, Hamish A. | Hearing ability is not a risk factor for admission to aged residential care of older persons in New Zealand | Non frailty patients nor considerer as a frailty patients |
| Jamieson, Hamish; Abey-Nesbit, Rebecca; Bergler, Ulrich; Keeling, Sally; Schluter, Philip J.; Scrase, Richard; Lacey, Cameron | Evaluating the Influence of Social Factors on Aged Residential Care Admission in a National Home Care Assessment Database of Older Adults | Non frailty patients nor considerer as a frailty patients |
| Hoffman, Geoffrey J.; Min, Lillian C.; Liu, Haiyin; Marciniak, Dan J.; Mody, Lona | Role of Post-Acute Care in Readmissions for Preexisting Healthcare-Associated Infections | Non frailty patients nor considerer as a frailty patients |
| de Man, Yvonne; Atsma, Femke; Jonkers, Wilma; de Rooij, Sophia E. J. A.; Westert, Gert P.; Jeurissen, Patrick P. T.; Groenewoud, A. Stef | Care trajectories of chronically ill older adult patients discharged from hospital: a quantitative cross-sectional study using health insurance claims data | Non frailty patients nor considerer as a frailty patients |
| Weerahandi, Himali; Bao, Haikun; Herrin, Jeph; Dharmarajan, Kumar; Ross, Joseph S.; Jones, Simon; Horwitz, Leora I. | Home Health Care After Skilled Nursing Facility Discharge Following Heart Failure Hospitalization | Non frailty patients nor considerer as a frailty patients |
| Paredes, Anghela Z.; Malik, Azeem T.; Cluse, Marcus; Strassels, Scott A.; Santry, Heena R.; Eiferman, Daniel; Jones, Christian; Vazquez, Daniel | Discharge disposition to skilled nursing facility after emergent general surgery predicts a poor prognosis | Non frailty patients nor considerer as a frailty patients |
| Popescu, Ioana; Sood, Neeraj; Joshi, Sushant; Huckfeldt, Peter; Escarce, Jose; Nuckols, Teryl K. | Trends in the Use of Skilled Nursing Facility and Home Health Care Under the Hospital Readmissions Reduction Program An Interrupted Time-series Analysis | Non frailty patients nor considerer as a frailty patients |
| **Authors** | **Study title** | **Exclusion reason** |
| Rofu, Aseel; Boulos, Daniel; Hanna, Mary; Jackson, Bruce; Coutsouvelis, John; Mak, Vivienne; Rofu, Noor; Egorova, Tatyana; Uruthirasigna, Nivethikga; Bhatia, Kiren; Kirsa, Sue | Validation of a risk stratification tool for a Hospital Outreach Medication Review (HOMR) program | Non frailty patients nor considerer as a frailty patients |
| Simpson, Michelle; Tejada, Jonny Macias; Driscoll, Amy; Singh, Maharaj; Klein, Matthew; Malone, Michael | The Bundled Hospital Elder Life Program-HELP and HELP in Home Care-and Its Association With Clinical Outcomes Among Older Adults Discharged to Home Healthcare | Non frailty patients nor considerer as a frailty patients |
| Yu, Jenny Jia; Sunderland, Yana | Outcomes of hospital in the home treatment of acute decompensated congestive cardiac failure compared to traditional in-hospital treatment in older patients | Non frailty patients nor considerer as a frailty patients |
| Seamer, Paul; Brake, Simon; Moore, Patrick; Mohammed, Mohammed A.; Wyatt, Steven | Did government spending cuts to social care for older people lead to an increase in emergency hospital admissions? An ecological study, England 2005-2016 | Non frailty patients nor considerer as a frailty patients |
| Tanderup, Anette; Ryg, Jesper; Rosholm, Jens-Ulrik; Lassen, Annmarie Touborg | Association between the level of municipality healthcare services and outcome among acutely older patients in the emergency department: a Danish population-based cohort study | Non frailty patients nor considerer as a frailty patients |
| Cunha Ferre, Maria Florencia; Gallo Acosta, Cristian Matias; Ruth Dawidowski, Adriana; Beatriz Senillosa, Monica; Maria Scozzafava, Silvana; Matias Saimovici, Javier | 72-hour hospital readmission of older people after hospital discharge with home care services | Non frailty patients nor considerer as a frailty patients |
| Bick, Irene; Dowding, Dawn | Hospitalization risk factors of older cohorts of home health care patients: A systematic review | Non frailty patients nor considerer as a frailty patients |
| Ang, Ian Yi Han; Tan, Chuen Seng; Nurjono, Milawaty; Tan, Xin Quan; Koh, Gerald Choon-Huat; Vrijhoef, Hubertus Johannes Maria; Tan, Shermin; Ng, Shu Ee; Toh, Sue-Anne | Retrospective evaluation of healthcare utilisation and mortality of two post-discharge care programmes in Singapore | Non frailty patients nor considerer as a frailty patients |
| Werner, Rachel M.; Coe, Norma B.; Qi, Mingyu; Konetzka, Tamara | Patient Outcomes After Hospital Discharge to Home With Home Health Care vs to a Skilled Nursing Facility | Non frailty patients nor considerer as a frailty patients |
| Kandlakunta, Harika; Anugwom, Chimaobi M.; Obaitan, Itegbemie; Singh, Dhruv P.; Wadhwa, Vaibhav; Garg, Sushil Kumar | INCIDENCE AND PREDICTORS OF 30-DAY READMISSION FOLLOWING COLECTOMY FOR COLON CANCER: AN ANALYSIS OF THE NATIONAL READMISSION DATABASE | Non frailty patients nor considerer as a frailty patients |
| Lockwood, Kylee J.; Harding, Katherine E.; Boyd, Jude N.; Taylor, Nicholas F. | Predischarge home visits after hip fracture: a randomized controlled trial | Non frailty patients nor considerer as a frailty patients |
| Stolz, Erwin; Mayerl, Hannes; Rasky, Eva; Freidl, Wolfgang | Individual and country-level determinants of nursing home admission in the last year of life in Europe | Non frailty patients nor considerer as a frailty patients |
| Vejux, Julien; Ben-Sadoun, Gregory; Piolet, Delphine; Bernat, Valerie; Ould-Aoudia, Vincent; Berrut, Gilles | Screening risk and protective factors of nursing home admission | Non frailty patients nor considerer as a frailty patients |
| DeCherrie, Linda V.; Wajnberg, Ania; Soones, Tacara; Escobar, Christian; Catalan, Elisse; Lubetsky, Sara; Leff, Bruce; Federman, Alex; Siu, Albert | Hospital at Home-Plus: A Platform of Facility-Based Care | Non frailty patients nor considerer as a frailty patients |
| Czarnecki, Andrew; Austin, Peter C.; Fremes, Stephen E.; Tu, Jack V.; Wijeysundera, Harindra C.; Ko, Dennis T. | Association between transitional care factors and hospital readmission after transcatheter aortic valve replacement: a retrospective observational cohort study | Non frailty patients nor considerer as a frailty patients |
| **Authors** | **Study title** | **Exclusion reason** |
| Logan, Diane R. | Transition From Hospital to Home: The Role of the Nurse Case Manager in Promoting Medication Adherence in the Medicare Population | Non frailty patients nor considerer as a frailty patients |
| Padula, Maria S.; D'Ambrosio, Gaetano G.; Tocci, Marina; D'Amico, Roberto; Banchelli, Federico; Angeli, Letizia; Scarpa, Marina; Capelli, Oreste; Cricelli, Claudio; Boriani, Giuseppe | Home care for heart failure: can caregiver education prevent hospital admissions? A randomized trial in primary care | Non frailty patients nor considerer as a frailty patients |
| Berggren, Monica; Karlsson, Asa; Lindelof, Nina; Englund, Undis; Olofsson, Birgitta; Nordstrom, Peter; Gustafson, Yngve; Stenvall, Michael | Effects of geriatric interdisciplinary home rehabilitation on complications and readmissions after hip fracture: a randomized controlled trial | Non frailty patients nor considerer as a frailty patients |
| Mengelers, Angela M. H. J.; Bleijlevens, Michel H. C.; Verbeek, Hilde; Capezuti, Elizabeth; Tan, Frans E. S.; Hamers, Jan P. H. | Professional and family caregivers' attitudes towards involuntary treatment in community-dwelling people with dementia | Non frailty patients nor considerer as a frailty patients |
| Tanderup, Anette; Lassen, Annmarie Touborg; Rosholm, Jens-Ulrik; Ryg, Jesper | Disability and morbidity among older patients in the emergency department: a Danish population-based cohort study | Non frailty patients nor considerer as a frailty patients |
| Dahlberg, Lena; Agahi, Neda; Schon, Par; Lennartsson, Carin | Planned and Unplanned Hospital Admissions and Their Relationship with Social Factors: Findings from a National, Prospective Study of People Aged 76 Years or Older | Non frailty patients nor considerer as a frailty patients |
| Middleton, Addie; Graham, James E.; Bettger, Janet Prvu; Haas, Allen; Ottenbacher, Kenneth J. | Facility and Geographic Variation in Rates of Successful Community Discharge After Inpatient Rehabilitation Among Medicare Fee-for-Service Beneficiaries | Non frailty patients nor considerer as a frailty patients |
| Levin, Kovi; Borg, Brigitte; Miller, Belinda; Kee, Kirk; Dabscheck, Eli | Characteristics of patients who progress from bridging to long-term oxygen therapy | Non frailty patients nor considerer as a frailty patients |
| Balentine, Courtney J.; Kenzik, Kelly; Chu, Daniel, I; Morris, Melanie S.; Knight, Sara J.; Brown, Cynthia J.; Bhatia, Smita | Planning post-discharge destination for gastrointestinal surgery patients: Room for improvement? | Non frailty patients nor considerer as a frailty patients |
| Makela, Petra; Godfrey, Mary; Cradduck-Bamford, Andrea; Ellis, Graham; Shepperd, Sasha | A protocol for the process evaluation of a multi-centre randomised trial to compare the effectiveness of geriatrician-led admission avoidance hospital at home versus inpatient admission | Non frailty patients nor considerer as a frailty patients. Non RCT |
| Helvik, Anne-Sofie; Selbaek, Geir; Benth, Jurate Saltyte; Roen, Irene; Bergh, Sverre | The course of neuropsychiatric symptoms in nursing home residents from admission to 30-month follow-up | Non frailty patients nor considerer as a frailty patients |
| Sevilla-Cazes, Jonathan; Ahmad, Faraz S.; Bowles, Kathryn H.; Jaskowiak, Anne; Gallagher, Tom; Goldberg, Lee R.; Kangovi, Shreya; Alexander, Madeline; Riegel, Barbara; Barg, Frances K.; Kimmel, Stephen E. | Heart Failure Home Management Challenges and Reasons for Readmission: a Qualitative Study to Understand the Patient's Perspective | Non frailty patients nor considerer as a frailty patients |
| Gruneir, Andrea; Fung, Kinwah; Fischer, Hadas D.; Bronskill, Susan E.; Panjwani, Dilzayn; Bell, Chaim M.; Dhalla, Irfan; Rochon, Paula A.; Anderson, Geoff | Care setting and 30-day hospital readmissions among older adults: a population-based cohort study | Non frailty patients nor considerer as a frailty patients |
| Konetzka, R. Tamara; Stuart, Elizabeth A.; Werner, Rachel M. | The effect of integration of hospitals and post-acute care providers on Medicare payment and patient outcomes | Non frailty patients nor considerer as a frailty patients |

| **Authors** | **Study title** | **Exclusion reason** |
| --- | --- | --- |
| Reistetter, Timothy A.; Eschbach, Karl; Prochaska, John; Jupiter, Daniel C.; Hong, Ickpyo; Haas, Allen M.; Ottenbacher, Kenneth J. | Understanding Variation in Postacute Care Developing Rehabilitation Service Areas Through Geographic Mapping | Interventions and/or outcomes do not meet criteria Non included main outcomes nor studied interventions. Nor Randomized Control Trials (RCT) |
| Mitsutake, Seigo; Ishizaki, Tatsuro; Tsuchiya-Ito, Rumiko; Uda, Kazuaki; Teramoto, Chie; Shimizu, Sayuri; Ito, Hideki | Associations of Hospital Discharge Services With Potentially Avoidable Readmissions Within 30 Days Among Older Adults After Rehabilitation in Acute Care Hospitals in Tokyo, Japan | Interventions and/or outcomes do not meet criteria Non included main outcomes nor studied interventions. Nor RCT |
| Weerahandi, Himali; Bao, Haikun; Herrin, Jeph; Dharmarajan, Kumar; Ross, Joseph S.; Jones, Simon; Horwitz, Leora I. | Home Health Care After Skilled Nursing Facility Discharge Following Heart Failure Hospitalization | Interventions and/or outcomes do not meet criteria Non included main outcomes nor studied interventions. Nor RCT |
| Paredes, Anghela Z.; Malik, Azeem T.; Cluse, Marcus; Strassels, Scott A.; Santry, Heena R.; Eiferman, Daniel; Jones, Christian; Vazquez, Daniel | Discharge disposition to skilled nursing facility after emergent general surgery predicts a poor prognosis | Interventions and/or outcomes do not meet criteria Non included main outcomes nor studied interventions. Nor RCT |
| Popescu, Ioana; Sood, Neeraj; Joshi, Sushant; Huckfeldt, Peter; Escarce, Jose; Nuckols, Teryl K. | Trends in the Use of Skilled Nursing Facility and Home Health Care Under the Hospital Readmissions Reduction Program An Interrupted Time-series Analysis | Interventions and/or outcomes do not meet criteria Non included main outcomes nor studied interventions. Nor RCT |
| Rofu, Aseel; Boulos, Daniel; Hanna, Mary; Jackson, Bruce; Coutsouvelis, John; Mak, Vivienne; Rofu, Noor; Egorova, Tatyana; Uruthirasigna, Nivethikga; Bhatia, Kiren; Kirsa, Sue | Validation of a risk stratification tool for a Hospital Outreach Medication Review (HOMR) program | Interventions and/or outcomes do not meet criteria Non included main outcomes nor studied interventions. Nor RCT |
| Simpson, Michelle; Tejada, Jonny Macias; Driscoll, Amy; Singh, Maharaj; Klein, Matthew; Malone, Michael | The Bundled Hospital Elder Life Program-HELP and HELP in Home Care-and Its Association With Clinical Outcomes Among Older Adults Discharged to Home Healthcare | Interventions and/or outcomes do not meet criteria Non included main outcomes nor studied interventions. Nor RCT |
| Yu, Jenny Jia; Sunderland, Yana | Outcomes of hospital in the home treatment of acute decompensated congestive cardiac failure compared to traditional in-hospital treatment in older patients | Interventions and/or outcomes do not meet criteria Non included main outcomes nor studied interventions. Nor RCT |
| Seamer, Paul; Brake, Simon; Moore, Patrick; Mohammed, Mohammed A.; Wyatt, Steven | Did government spending cuts to social care for older people lead to an increase in emergency hospital admissions? An ecological study, England 2005-2016 | Interventions and/or outcomes do not meet criteria Non included main outcomes nor studied interventions. Nor RCT |
| Tanderup, Anette; Ryg, Jesper; Rosholm, Jens-Ulrik; Lassen, Annmarie Touborg | Association between the level of municipality healthcare services and outcome among acutely older patients in the emergency department: a Danish population-based cohort study | Interventions and/or outcomes do not meet criteria Non included main outcomes nor studied interventions. Nor RCT |
| Cunha Ferre, Maria Florencia; Gallo Acosta, Cristian Matias; Ruth Dawidowski, Adriana; Beatriz Senillosa, Monica; Maria Scozzafava, Silvana; Matias Saimovici, Javier | 72-hour hospital readmission of older people after hospital discharge with home care services | Interventions and/or outcomes do not meet criteria Non included main outcomes nor studied interventions. Nor RCT |
| Bick, Irene; Dowding, Dawn | Hospitalization risk factors of older cohorts of home health care patients: A systematic review | Interventions and/or outcomes do not meet criteria Non included main outcomes nor studied interventions. Nor RCT |
| Werner, Rachel M.; Coe, Norma B.; Qi, Mingyu; Konetzka, Tamara | Patient Outcomes After Hospital Discharge to Home With Home Health Care vs to a Skilled Nursing Facility | Interventions and/or outcomes do not meet criteria Non included main outcomes nor studied interventions. Nor RCT |
| **Authors** | **Study title** | **Exclusion reason** |
| Kandlakunta, Harika; Anugwom, Chimaobi M.; Obaitan, Itegbemie; Singh, Dhruv P.; Wadhwa, Vaibhav; Garg, Sushil Kumar | INCIDENCE AND PREDICTORS OF 30-DAY READMISSION FOLLOWING COLECTOMY FOR COLON CANCER: AN ANALYSIS OF THE NATIONAL READMISSION DATABASE | Interventions and/or outcomes do not meet criteria Non included main outcomes nor studied interventions. Nor RCT |
| Briquet, Caroline; Cornu, Olivier; Servais, Valerie; Blasson, Chloe; Vandeleene, Bernard; Yildiz, Halil; Stainier, Annabelle; Yombie, Jean Cyr | Clinical characteristics and outcomes of patients receiving outpatient parenteral antibiotic therapy in a Belgian setting: a single-center pilot study | Interventions and/or outcomes do not meet criteria Non included main outcomes nor studied interventions. Nor RCT |
| Bowles, Kathryn H.; Ratcliffe, Sarah J.; Holmes, John H.; Keim, Sue; Potashnik, Sheryl; Flores, Emilia; Humbrecht, Diane; Whitehouse, Christina R.; Naylor, Mary D. | Using a Decision Support Algorithm for Referrals to Post-Acute Care | Interventions and/or outcomes do not meet criteria Non included main outcomes nor studied interventions. Nor RCT |
| Flanagan, Priti; Kelly, Ronald | Discharge Home From Hospital: How DIRE Can It Be? | Interventions and/or outcomes do not meet criteria Non included main outcomes nor studied interventions. Nor RCT |
| Stolz, Erwin; Mayerl, Hannes; Rasky, Eva; Freidl, Wolfgang | Individual and country-level determinants of nursing home admission in the last year of life in Europe | Interventions and/or outcomes do not meet criteria Non included main outcomes nor studied interventions. Nor RCT |
| Vejux, Julien; Ben-Sadoun, Gregory; Piolet, Delphine; Bernat, Valerie; Ould-Aoudia, Vincent; Berrut, Gilles | Screening risk and protective factors of nursing home admission | Interventions and/or outcomes do not meet criteria Non included main outcomes nor studied interventions. Nor RCT |
| DeCherrie, Linda V.; Wajnberg, Ania; Soones, Tacara; Escobar, Christian; Catalan, Elisse; Lubetsky, Sara; Leff, Bruce; Federman, Alex; Siu, Albert | Hospital at Home-Plus: A Platform of Facility-Based Care | Interventions and/or outcomes do not meet criteria Non included main outcomes nor studied interventions. Nor RCT |
| Czarnecki, Andrew; Austin, Peter C.; Fremes, Stephen E.; Tu, Jack V.; Wijeysundera, Harindra C.; Ko, Dennis T. | Association between transitional care factors and hospital readmission after transcatheter aortic valve replacement: a retrospective observational cohort study | Interventions and/or outcomes do not meet criteria Non included main outcomes nor studied interventions. Nor RCT |
| Logan, Diane R. | Transition From Hospital to Home: The Role of the Nurse Case Manager in Promoting Medication Adherence in the Medicare Population | Interventions and/or outcomes do not meet criteria Non included main outcomes nor studied interventions. Nor RCT |
| Mengelers, Angela M. H. J.; Bleijlevens, Michel H. C.; Verbeek, Hilde; Capezuti, Elizabeth; Tan, Frans E. S.; Hamers, Jan P. H. | Professional and family caregivers' attitudes towards involuntary treatment in community-dwelling people with dementia | Interventions and/or outcomes do not meet criteria Non included main outcomes nor studied interventions. Nor RCT |
| Tanderup, Anette; Lassen, Annmarie Touborg; Rosholm, Jens-Ulrik; Ryg, Jesper | Disability and morbidity among older patients in the emergency department: a Danish population-based cohort study | Interventions and/or outcomes do not meet criteria Non included main outcomes nor studied interventions. Nor RCT |
| Dahlberg, Lena; Agahi, Neda; Schon, Par; Lennartsson, Carin | Planned and Unplanned Hospital Admissions and Their Relationship with Social Factors: Findings from a National, Prospective Study of People Aged 76 Years or Older | Interventions and/or outcomes do not meet criteria Non included main outcomes nor studied interventions. Nor RCT |
| Adsersen, Mathilde; Thygesen, Lau Caspar; Neergaard, Mette Asbjoern; Jensen, Anders Bonde; Sjogren, Per; Damkier, Anette; Clausen, Lars Michael; Groenvold, Mogens | Cohabitation Status Influenced Admittance to Specialized Palliative Care for Cancer Patients: A Nationwide Study from the Danish Palliative Care Database | Interventions and/or outcomes do not meet criteria Non included main outcomes nor studied interventions. Nor RCT |
| Cochran, Allyson R.; Raub, Kyle M.; Murphy, Keith J.; Iannitti, David A.; Vrochides, Dionisios | Novel use of REDCap to develop an advanced platform to display predictive analytics and track compliance with Enhanced Recovery After Surgery for pancreaticoduodenectomy | Interventions and/or outcomes do not meet criteria Non included main outcomes nor studied interventions. Nor RCT |
| **Authors** | **Study title** | **Exclusion reason** |
| Middleton, Addie; Graham, James E.; Bettger, Janet Prvu; Haas, Allen; Ottenbacher, Kenneth J. | Facility and Geographic Variation in Rates of Successful Community Discharge After Inpatient Rehabilitation Among Medicare Fee-for-Service Beneficiaries | Interventions and/or outcomes do not meet criteria Non included main outcomes nor studied interventions. Nor RCT |
| Mroz, Tracy M.; Meadow, Ann; Colantuoni, Elizabeth; Leff, Bruce; Wolff, Jennifer L. | Home Health Agency Characteristics and Quality Outcomes for Medicare Beneficiaries With Rehabilitation-Sensitive Conditions | Interventions and/or outcomes do not meet criteria Non included main outcomes nor studied interventions. Nor RCT |
| Drake, Rachel; Ozols, Audrey; Nadeau, William J.; Braid-Forbes, Mary Jo | Hospital Inpatient Admissions With Dehydration and/or Malnutrition in Medicare Beneficiaries Receiving Enteral Nutrition: A Cohort Study | Interventions and/or outcomes do not meet criteria Non included main outcomes nor studied interventions. Nor RCT |
| Valtorta, Nicole K.; Moore, Danielle Collingridge; Barron, Lynn; Stow, Daniel; Hanratty, Barbara | Older Adults' Social Relationships and Health Care Utilization: A Systematic Review | Interventions and/or outcomes do not meet criteria Non included main outcomes nor studied interventions. Nor RCT |
| Tsunoda, Aki; Kido, Yoshifumi; Kayama, Mami | Japanese Outreach Model Project for patients who have difficulty maintaining contact with mental health services: Comparison of care between higher-functioning and lower-functioning groups | Interventions and/or outcomes do not meet criteria Non included main outcomes nor studied interventions. Nor RCT |
| Athilingam, Ponrathi; Jenkins, Bradlee A.; Zumpano, Heather; Labrador, Miguel A. | Mobile technology to improve heart failure outcomes: A proof of concept paper | Interventions and/or outcomes do not meet criteria Non included main outcomes nor studied interventions. Nor RCT |
| Abtan, Robert; Rotondi, Nooshin Khobzi; Macpherson, Alison; Rotondi, Michael Anthony | The effect of informal caregiver support on utilization of acute health services among home care clients: a prospective observational study | Interventions and/or outcomes do not meet criteria Non included main outcomes nor studied interventions. Nor RCT |
| Mann, Elizabeth; Zepeda, Orlando; Soones, Tacara; Federman, Alex; Leff, Bruce; Siu, Albert; Boockvar, Kenneth | Adverse drug events and medication problems in "Hospital at Home" patients | Interventions and/or outcomes do not meet criteria Non included main outcomes nor studied interventions. Nor RCT |
| La Manna, Jacqueline B.; Bushy, Angeline; Gammonley, Denise | Post-hospitalization experiences of older adults diagnosed with diabetes: "It was daunting!" | Interventions and/or outcomes do not meet criteria Non included main outcomes nor studied interventions. Nor RCT |
| Freburger, Janet K.; Li, Dongmei; Fraher, Erin P. | Community Use of Physical and Occupational Therapy After Stroke and Risk of Hospital Readmission | Interventions and/or outcomes do not meet criteria Non included main outcomes nor studied interventions. Nor RCT |
| Tsai, Hsiu-Hsin; Tsai, Yun-Fang | Development, validation and testing of a nursing home to emergency room transfer checklist | Interventions and/or outcomes do not meet criteria Non included main outcomes nor studied interventions. Nor RCT |
| Mehta, Hemalkumar B.; Hughes, Byron D.; Sieloff, Eric; Sura, Sneha O.; Shan, Yong; Adhikari, Deepak; Senagore, Anthony | Outcomes of Laparoscopic Colectomy in Younger and Older Patients: An Analysis of Nationwide Readmission Database | Interventions and/or outcomes do not meet criteria Non included main outcomes nor studied interventions. Nor RCT |
| Jones, Masha G.; Ornstein, Katherine A.; Skovran, David M.; Soriano, Theresa A.; DeCherrie, Linda V. | Characterizing the high-risk homebound patients in need of nurse practitioner co-management | Interventions and/or outcomes do not meet criteria Non included main outcomes nor studied interventions. Nor RCT |
| Eliadi, Irene; Tsoumi, Georgia; Kampouropoulou, Olga; Theofanis, Vasileios; Mantzourani, Marina; Samarkos, Michael | Characterization of the medical admissions in a tertiary Greek hospital | Interventions and/or outcomes do not meet criteria Non included main outcomes nor studied interventions. Nor RCT |
| **Authors** | **Study title** | **Exclusion reason** |
| Martin, Stacy; Anderson, Bill; Vincenzo, Jennifer L.; Zai, Sajid Yousuf | A Retrospective Comparison of Home Telehealth and Nursing Care With or Without Rehabilitation Therapy on Rehospitalization Rates of Individuals With Heart Failure | Interventions and/or outcomes do not meet criteria Non included main outcomes nor studied interventions. Nor RCT |
| Corbett, Lisa Q.; Funk, Marjorie; Fortunato, Gilbert; O'Sullivan, David M. | Pressure Injury in a Community Population A Descriptive Study | Interventions and/or outcomes do not meet criteria Non included main outcomes nor studied interventions. Nor RCT |
| Yim, Cindi K.; Barron, Yolanda; Moore, Stanley; Murtaugh, Chris; Lala, Anuradha; Aldridge, Melissa; Goldstein, Nathan; Gelfman, Laura P. | Hospice Enrollment in Patients With Advanced Heart Failure Decreases Acute Medical Service Utilization | Interventions and/or outcomes do not meet criteria Non included main outcomes nor studied interventions. Nor RCT |
| Lalmolda, C.; Coll-Fernandez, R.; Martinez, N.; Bare, M.; Colet, M. Teixido; Epelde, F.; Monso, E. | Effect of a rehabilitation-based chronic disease management program targeting severe COPD exacerbations on readmission patterns | Interventions and/or outcomes do not meet criteria Non included main outcomes nor studied interventions. Nor RCT |
| Kuluski, Kerry; Gandhi, Sima; Diong, Christina; Gray, Carolyn Steele; Bronskill, Susan E. | Patterns of community follow-up, subsequent health service use and survival among young and mid-life adults discharged from chronic care hospitals: a retrospective cohort study | Interventions and/or outcomes do not meet criteria Non included main outcomes nor studied interventions. Nor RCT |
| Heckel, M.; Stiel, S.; Frauendorf, T.; Hanke, R. M.; Ostgathe, C. | Comparison of Patients and their Care in Urban and Rural Specialised Palliative Home Care - A Single Service Analysis | Interventions and/or outcomes do not meet criteria Non included main outcomes nor studied interventions. Nor RCT |
| Kothari, Anai N.; Yau, Ryan M.; Blackwell, Robert H.; Schaidle-Blackburn, Colleen; Markossian, Talar; Zapf, Matthew A. C.; Lu, Amy D.; Kuo, Paul C. | Inpatient Rehabilitation after Liver Transplantation Decreases Risk and Severity of 30-Day Readmissions | Interventions and/or outcomes do not meet criteria Non included main outcomes nor studied interventions. Nor RCT |
| Kasteridis, Panagiotis; Mason, Anne; Goddard, Maria; Jacobs, Rowena; Santos, Rita; Rodriguez-Sanchez, Beatriz; McGonigal, Gerard | Risk of Care Home Placement following Acute Hospital Admission: Effects of a Pay-for-Performance Scheme for Dementia | Interventions and/or outcomes do not meet criteria Non included main outcomes nor studied interventions. Nor RCT |
| Zhang, Ning; Li, Yue; Rodriguez-Monguio, Rosa; Barenberg, Andrew; Temkin-Greener, Helena; Gurwitz, Jerry | Are Obese Residents More Likely to Be Admitted to Nursing Homes That Have More Deficiencies in Care? | Interventions and/or outcomes do not meet criteria Non included main outcomes nor studied interventions. Nor RCT |
| Pivodic, Lara; Pardon, Koen; Miccinesi, Guido; Vega Alonso, Tomas; Moreels, Sarah; Donker, Ge A.; Arrieta, Enrique; Onwuteaka-Philipsen, Bregje D.; Deliens, Luc; Van den Block, Lieve | Hospitalisations at the end of life in four European countries: a population-based study via epidemiological surveillance networks | Interventions and/or outcomes do not meet criteria Non included main outcomes nor studied interventions. Nor RCT |
| Sinn, Chi-Ling Joanna; Tran, Jake; Pauley, Tim; Hirdes, John | Predicting Adverse Outcomes After Discharge From Complex Continuing Care Hospital Settings to the Community | Interventions and/or outcomes do not meet criteria Non included main outcomes nor studied interventions. Nor RCT |
| Font, C.; Fernandez-Aviles, F.; Calderon, C.; Garcia-Fernandez, T.; Arab, N.; Pineda, E.; Buxo, E.; Ayora, P.; Carreno, M.; Pereira, V.; Viladot, M.; Moreno, C.; Gallego, C.; Hernando, A.; Creus, N.; Barrera, C.; Alcaraz, R.; Sanchez, J.; Prat, A.; Tuca, A. | Home management of acute medical complications in cancer patients: a prospective pilot study | Interventions and/or outcomes do not meet criteria Non included main outcomes nor studied interventions. Nor RCT |
| Ihl, Ralf; Cujai, Nadine; Krah, Katrin | Admission into a Nursing Home Delay or prevention with the use of a complete support network? | Interventions and/or outcomes do not meet criteria Non included main outcomes nor studied interventions. Nor RCT |
| Kang, Youjeong; McHugh, Matthew D.; Chittams, Jesse; Bowles, Kathryn H. | Utilizing Home Healthcare Electronic Health Records for Telehomecare Patients With Heart Failure A Decision Tree Approach to Detect Associations With Rehospitalizations | Interventions and/or outcomes do not meet criteria Non included main outcomes nor studied interventions. Nor RCT |
| **Authors** | **Study title** | **Exclusion reason** |
| Hill, A. D.; Fowler, R. A.; Pinto, R.; Herridge, M. S.; Cuthbertson, B. H.; Scales, D. C. | Long-term outcomes and healthcare utilization following critical illness - a population-based study | Interventions and/or outcomes do not meet criteria Non included main outcomes nor studied interventions. Nor RCT |
| Chen, Hsueh-Fen; Carlson, Erin; Popoola, Taiye; Suzuki, Sumihiro | The Impact of Rurality on 30-Day Preventable Readmission, Illness Severity, and Risk of Mortality for Heart Failure Medicare Home Health Beneficiaries | Interventions and/or outcomes do not meet criteria Non included main outcomes nor studied interventions. Nor RCT |
| Sacks, Greg D.; Lawson, Elise H.; Dawes, Aaron J.; Weiss, Robert E.; Russell, Marcia M.; Brook, Robert H.; Zingmond, David S.; Ko, Clifford Y. | Variation in Hospital Use of Postacute Care After Surgery and the Association With Care Quality | Interventions and/or outcomes do not meet criteria Non included main outcomes nor studied interventions. Nor RCT |
| Oliver, David | David Oliver: Keeping care home residents out of hospital | Interventions and/or outcomes do not meet criteria Non included main outcomes nor studied interventions. Nor RCT |
| Vitacca, Michele; Fumagalli, Lia Paola; Borghi, Gabriella; Colombo, Fausto; Castelli, Alberto; Scalvini, Simonetta; Masella, Cristina | Home-Based Telemanagement in Advanced COPD: Who Uses it Most? Real-Life Study in Lombardy | Interventions and/or outcomes do not meet criteria Non included main outcomes nor studied interventions. Nor RCT |
| Jubelt, Lindsay E.; Goldfeld, Keith S.; Chung, Wei-yi; Blecker, Saul B.; Horwitz, Leora I. | Changes in Discharge Location and Readmission Rates Under Medicare Bundled Payment | Interventions and/or outcomes do not meet criteria Non included main outcomes nor studied interventions. Nor RCT |
| Reed, Richard L.; Isherwood, Linda; Ben-Tovim, David | Why do older people with multi-morbidity experience unplanned hospital admissions from the community: a root cause analysis | Interventions and/or outcomes do not meet criteria Non included main outcomes nor studied interventions. Nor RCT |
| Mabire, Cedric; Buela, Christophe; Morin, Diane; Goulet, Celine | Nursing discharge planning for older medical inpatients in Switzerland: A cross-sectional study | Interventions and/or outcomes do not meet criteria Non included main outcomes nor studied interventions. Nor RCT |
| Young, Yuchi; Kalamaras, John; Kelly, Lindsay; Hornick, David; Yucel, Recai | Is Aging in Place Delaying Nursing Home Admission? | Interventions and/or outcomes do not meet criteria Non included main outcomes nor studied interventions. Nor RCT |
| Acher, Alexandra W.; LeCaire, Tamara J.; Hundt, Ann Schoofs; Greenberg, Caprice C.; Carayon, Pascale; Kind, Amy J.; Weber, Sharon M. | Using Human Factors and Systems Engineering to Evaluate Readmission after Complex Surgery | Interventions and/or outcomes do not meet criteria Non included main outcomes nor studied interventions. Nor RCT |
| Kalista, Tom; Lemay, Virginia; Cohen, Lisa | Postdischarge community pharmacist provided home services for patients after hospitalization for heart failure | Interventions and/or outcomes do not meet criteria Non included main outcomes nor studied interventions. Nor RCT |
| Qaddoura, Amro; Yazdan-Ashoori, Payam; Kabali, Conrad; Thabane, Lehana; Haynes, R. Brian; Connolly, Stuart J.; Van Spall, Harriette Gillian Christine | Efficacy of Hospital at Home in Patients with Heart Failure: A Systematic Review and Meta-Analysis | Interventions and/or outcomes do not meet criteria Non included main outcomes nor studied interventions. Nor RCT |
| Chen, Hsueh-Fen; Popoola, Taiye; Radhakrishnan, Kavita; Suzuki, Sumihiro; Homan, Sharon | Improving Diabetic Patient Transition to Home Healthcare: Leading Risk Factors for 30-Day Readmission | Interventions and/or outcomes do not meet criteria Non included main outcomes nor studied interventions. Nor RCT |
| Mujal, A.; Sola, J.; Hernandez, M.; Villarino, M. -A.; Machado, M. -L.; Baylina, M.; Tajan, J.; Oristrell, J. | Safety and effectiveness of home intravenous antibiotic therapy for multidrug-resistant bacterial infections | Interventions and/or outcomes do not meet criteria Non included main outcomes nor studied interventions. Nor RCT |
| **Authors** | **Study title** | **Exclusion reason** |
| Shah, Tina; Churpek, Matthew M.; Perraillon, Marcelo Coca; Konetzka, R. Tamara | Understanding Why Patients With COPD Get Readmitted | Interventions and/or outcomes do not meet criteria Non included main outcomes nor studied interventions. Nor RCT |
| Risco, Ester; Cabrera, Esther; Jolley, David; Stephan, Astrid; Karlsson, Staffan; Verbeek, Hilde; Saks, Kai; Hupli, Maija; Sourdet, Sandrine; Zabalegui, Adelaida | The association between physical dependency and the presence of neuropsychiatric symptoms, with the admission of people with dementia to a long-term care institution: A prospective observational cohort study | Interventions and/or outcomes do not meet criteria Non included main outcomes nor studied interventions. Nor RCT |
| Kolte, Dhaval; Khera, Sahil; Aronow, Wilbert S.; Palaniswamy, Chandrasekar; Mujib, Marjan; Ahn, Chul; Iwai, Sei; Jain, Diwakar; Sule, Sachin; Ahmed, Ali; Cooper, Howard A.; Frishman, William H.; Bhatt, Deepak L.; Panza, Julio A.; Fonarow, Gregg C. | Regional Variation in the Incidence and Outcomes of In-Hospital Cardiac Arrest in the United States | Interventions and/or outcomes do not meet criteria Non included main outcomes nor studied interventions. Nor RCT |
| Hall, Rasheeda K.; Toles, Mark; Massing, Mark; Jackson, Eric; Peacock-Hinton, Sharon; O'Hare, Ann M.; Colon-Emeric, Cathleen | Utilization of Acute Care among Patients with ESRD Discharged Home from Skilled Nursing Facilities | Interventions and/or outcomes do not meet criteria Non included main outcomes nor studied interventions. Nor RCT |
| Garland, Allan; Olafson, Kendiss; Ramsey, Clare D.; Yogendran, Marina; Fransoo, Randall | A Population-Based Observational Study of Intensive Care Unit-Related Outcomes With Emphasis on Post-Hospital Outcomes | Interventions and/or outcomes do not meet criteria Non included main outcomes nor studied interventions. Nor RCT |
| Chen, Christina Y.; Thorsteinsdottir, Bjorg; Cha, Stephen S.; Hanson, Gregory J.; Peterson, Stephanie M.; Rahman, Parvez A.; Naessens, James M.; Takahashi, Paul Y. | Health Care Outcomes and Advance Care Planning in Older Adults Who Receive Home-Based Palliative Care: A Pilot Cohort Study | Interventions and/or outcomes do not meet criteria Non included main outcomes nor studied interventions. Nor RCT |
| Cramer, Susanne; Fonager, Kirsten | Risk factors of 30-days re-hospitalization after Hospital at Home in a cohort of patients treated with parenteral therapy | Interventions and/or outcomes do not meet criteria Non included main outcomes nor studied interventions. Nor RCT |
| Langstaff, Caryn; Martin, Cally; Brown, Gwen; McGuinness, Don; Mather, Jo; Loshaw, Jennifer; Jones, Nancy; Fletcher, Kim; Paterson, John | Enhancing Community-Based Rehabilitation for Stroke Survivors: Creating a Discharge Link | Interventions and/or outcomes do not meet criteria Non included main outcomes nor studied interventions. Nor RCT |
| McCann, Mark; Grundy, Emily; O'Reilly, Dermot | Urban and rural differences in risk of admission to a care home: A census-based follow-up study | Interventions and/or outcomes do not meet criteria Non included main outcomes nor studied interventions. Nor RCT |
| Sanford, Dominic E.; Olsen, Margaret A.; Bommarito, Kerry M.; Shah, Manish; Fields, Ryan C.; Hawkins, William G.; Jaques, David P.; Linehan, David C. | Association of Discharge Home with Home Health Care and 30-Day Readmission after Pancreatectomy | Interventions and/or outcomes do not meet criteria Non included main outcomes nor studied interventions. Nor RCT |
| Olson, Catherine H.; Dierich, Mary; Westra, Bonnie L. | Automation of a high risk medication regime algorithm in a home health care population | Interventions and/or outcomes do not meet criteria Non included main outcomes nor studied interventions. Nor RCT |
| Mashaw, Arsheeya | IMPLEMENTATION OF A HOSPITAL READMISSIONS PREVENTION PROGRAM IN A RURAL GERIATRIC POPULATION | Interventions and/or outcomes do not meet criteria Non included main outcomes nor studied interventions. Nor RCT |
| Peel, Nancye M.; Navanathan, Sukumar; Hubbard, Ruth E. | Gait speed as a predictor of outcomes in post-acute transitional care for older people | Interventions and/or outcomes do not meet criteria Non included main outcomes nor studied interventions. Nor RCT |
| Stevenson, David G.; Dusetzina, Stacie B.; O'Malley, A. James; Mitchell, Susan L.; Zarowitz, Barbara J.; Chernew, Michael E.; Newhouse, Joseph P.; Huskamp, Haiden A. | High-Risk Medication Use by Nursing Home Residents Before and After Hospitalization | Interventions and/or outcomes do not meet criteria Non included main outcomes nor studied interventions. Nor RCT |
| **Authors** | **Study title** | **Exclusion reason** |
| Luscombe-Marsh, Natalie; Chapman, Ian; Visvanathan, Renuka | Hospital admissions in poorly nourished, compared with well-nourished, older South Australians receiving 'Meals on Wheels': Findings from a pilot study | Interventions and/or outcomes do not meet criteria Non included main outcomes nor studied interventions. Nor RCT |
| You, Emily (Chuanmei); Dunt, David Robert; White, Vanessa; Vander Hoorn, Stephen; Doyle, Colleen | Risk of death or hospital admission among community-dwelling older adults living with dementia in Australia | Interventions and/or outcomes do not meet criteria Non included main outcomes nor studied interventions. Nor RCT |
| Eastwood, Cathy A.; Howlett, Jonathan G.; King-Shier, Kathryn M.; McAlister, Finlay A.; Ezekowitz, Justin A.; Quan, Hude | Determinants of Early Readmission After Hospitalization for Heart Failure | Interventions and/or outcomes do not meet criteria Non included main outcomes nor studied interventions. Nor RCT |
| Cirillo, Massimo; Lunardi, Gianluigi; Coati, Francesca; Ciccarelli, Lucia; Alestra, Stefania; Mariotto, Manuela; Micheloni, Beniamino; Cassandrini, Paola Agnese; Inn, Alessandro; Magarotto, Roberto; Nicodemo, Maurizio; Picece, Vincenzo; Turazza, Monica; Gori, Stefania; Venturini, Marco | Management of oral anticancer drugs: feasibility and patient approval of a specific monitoring program | Interventions and/or outcomes do not meet criteria Non included main outcomes nor studied interventions. Nor RCT |
| Gennaro, Nicola; Maggi, Stefania; Pellizzari, Michele; Carlucci, Francesco; Pilotto, Alberto; Saugo, Mario | Early implementation of home care and 30 day readmissions in > 65 years Veneto region patients discharged for heart failure and with disability | Interventions and/or outcomes do not meet criteria Non included main outcomes nor studied interventions. Nor RCT |
| Ponzetti, Agostino; Lista, Patrizia; Pagano, Eva; Demichelis, Maria Maddalena; Ciuffreda, Libero; Ciccone, Giovannino | Role of multidimensional assessment of frailty in predicting short-term outcomes in hospitalized cancer patients: results of a prospective cohort study | Interventions and/or outcomes do not meet criteria Non included main outcomes nor studied interventions. Nor RCT |
| Chitnis, X. A.; Georghiou, T.; Steventon, A.; Bardsley, M. J. | El;ffect of a home-based end-of-life nursing service on hospital use at the end of life and place of death: a study using administrative data and matched controls | Interventions and/or outcomes do not meet criteria Non included main outcomes nor studied interventions. Nor RCT |
| Seematter-Bagnoud, Laurence; Lecureux, Estelle; Rochat, Stephane; Monad, Stefanie; Lenoble-Hoskovec, Constanze; Buela, Christophe J. | Predictors of Functional Recovery in Patients Admitted to Geriatric Postacute Rehabilitation | Interventions and/or outcomes do not meet criteria Non included main outcomes nor studied interventions. Nor RCT |
| Hendrix, Cristina; Tepfer, Sara; Forest, Sabrina; Ziegler, Karen; Fox, Valerie; Stein, Jeannette; McConnell, Eleanor S.; Hastings, Susan Nicole; Schmader, Kenneth; Colon-Emeric, Cathleen | Transitional Care Partners: A hospital-to-home support for older adults and their caregivers | Interventions and/or outcomes do not meet criteria Non included main outcomes nor studied interventions. Nor RCT |
| Anderson, Sarah L.; Marrs, Joel C.; Vande Griend, Joseph P.; Hanratty, Rebecca | Implementation of a Clinical Pharmacy Specialist-Managed Telephonic Hospital Discharge Follow-Up Program in a Patient-Centered Medical Home | Interventions and/or outcomes do not meet criteria Non included main outcomes nor studied interventions. Nor RCT |
| Lopez-Liria, Remedios; Ferre-Salmeron, Rocio; Arrebola-Lopez, Clara; Granados-Valverde, Rocio; Angel Gobernado-Cabero, Miguel; Padilla-Gongora, David | Home-based rehabilitation in the functional recovery of patients with cerebrovascular disease | Interventions and/or outcomes do not meet criteria Non included main outcomes nor studied interventions. Nor RCT |
| Seetoh, Theresa; Lye, David C.; Cook, Alex R.; Archuleta, Sophia; Chan, Monica; Sulaiman, Zuraidah; Zhong, Lihua; Llorin, Ryan M.; Balm, Michelle; Fisher, Dale | An outcomes analysis of outpatient parenteral antibiotic therapy (OPAT) in a large Asian cohort | Interventions and/or outcomes do not meet criteria Non included main outcomes nor studied interventions. Nor RCT |
| **Authors** | **Study title** | **Exclusion reason** |
| Ding, Shih-Tan; Wang, Chuan-Lan; Huang, Yu-Han; Shu, Chin-Chung; Tseng, Yu-Tzu; Huang, Chun-Ta; Hsu, Nin-Chieh; Lin, Yu-Feng; Tsai, Hung-Bin; Yang, Ming-Chin; Ko, Wen-Je | Demand and Predictors for Post-Discharge Medical Counseling in Home Care Patients: A Prospective Cohort Study | Interventions and/or outcomes do not meet criteria Non included main outcomes nor studied interventions. Nor RCT |
| Gruneir, Andrea; Forrester, Jacqueline; Camacho, Ximena; Gill, Sudeep S.; Bronskill, Susan E. | Gender differences in home care clients and admission to long-term care in Ontario, Canada: a population-based retrospective cohort study | Interventions and/or outcomes do not meet criteria Non included main outcomes nor studied interventions. Nor RCT |
| Lau, Liza; Chong, Carol P.; Lim, Wen Kwang | Hospital treatment in residential care facilities is a viable alternative to hospital admission for selected patients | Interventions and/or outcomes do not meet criteria Non included main outcomes nor studied interventions. Nor RCT |
| Konetzka, R. Tamara; Polsky, Daniel; Werner, Rachel M. | Shipping out instead of shaping up: Rehospitalization from nursing homes as an unintended effect of public reporting | Interventions and/or outcomes do not meet criteria Non included main outcomes nor studied interventions. Nor RCT |
| Radhakrishnan, Kavita; Jacelon, Cynthia S.; Bigelow, Carol; Roche, Joan; Marquard, Jenna; Bowles, Kathryn H. | Use of a homecare electronic health record to find associations between patient characteristics and re-hospitalizations in patients with heart failure using telehealth | Interventions and/or outcomes do not meet criteria Non included main outcomes nor studied interventions. Nor RCT |
| Lukas, Lou; Foltz, Carol; Paxton, Hannah | Hospital Outcomes for a Home-Based Palliative Medicine Consulting Service | Interventions and/or outcomes do not meet criteria Non included main outcomes nor studied interventions. Nor RCT |
| Mueller-Buehl, Uwe; Leutgeb, Ruediger; Bungartz, Jessica; Szecsenyi, Joachim; Laux, Gunter | Expenditure of chronic venous leg ulcer management in German primary care: results from a population-based study | Interventions and/or outcomes do not meet criteria Non included main outcomes nor studied interventions. Nor RCT |
| Gorodeski, Eiran Z.; Chlad, Sandra; Vilensky, Seth | Home-based care for heart failure: Cleveland Clinic's "Heart Care at Home" transitional care program | Interventions and/or outcomes do not meet criteria Non included main outcomes nor studied interventions. Nor RCT |
| Takahashi, Paul Y.; Haas, Lindsey R.; Quigg, Stephanie M.; Croghan, Ivana T.; Naessens, James M.; Shah, Nilay D.; Hanson, Gregory J. | 30-day hospital readmission of older adults using care transitions after hospitalization: a pilot prospective cohort study | Interventions and/or outcomes do not meet criteria Non included main outcomes nor studied interventions. Nor RCT |
| Madigan, Elizabeth A.; Gordon, Nahida H.; Fortinsky, Richard H.; Koroukian, Siran M.; Pina, Ileana; Riggs, Jennifer S. | Rehospitalization in a National Population of Home Health Care Patients with Heart Failure | Interventions and/or outcomes do not meet criteria Non included main outcomes nor studied interventions. Nor RCT |
| Tao, Hong; Ellenbecker, Carol Hall; Chen, Jie; Zhan, Lin; Dalton, Joanne | The Influence of Social Environmental Factors on Rehospitalization Among Patients Receiving Home Health Care Services | Interventions and/or outcomes do not meet criteria Non included main outcomes nor studied interventions. Nor RCT |
| Tinetti, Mary E.; Charpentier, Peter; Gottschalk, Margaret; Baker, Dorothy I. | Effect of a Restorative Model of Posthospital Home Care on Hospital Readmissions | Interventions and/or outcomes do not meet criteria Non included main outcomes nor studied interventions. Nor RCT |
| Rueda, Juan C.; Jimenez, Andrea; Caro, Aleidis; Feliu, Francisco; Escuder, Jorge; Gris, Fernando; Spuch, Juan; Vicente, Vicente | Home Treatment of Uncomplicated Acute Diverticulitis | Interventions and/or outcomes do not meet criteria Non included main outcomes nor studied interventions. Nor RCT |
| Rosted, Elizabeth; Wagner, Lis; Hendriksen, Carsten; Poulsen, Ingrid | Geriatric nursing assessment and intervention in an emergency department: a pilot study | Interventions and/or outcomes do not meet criteria Non included main outcomes nor studied interventions. Nor RCT |
| McCann, Mark; Donnelly, Michael; O'Reilly, Dermot | Gender differences in care home admission risk: partner's age explains the higher risk for women | Interventions and/or outcomes do not meet criteria Non included main outcomes nor studied interventions. Nor RCT |
| **Authors** | **Study title** | **Exclusion reason** |
| Watkins, Lynn; Hall, Carol; Kring, Daria | Hospital to Home A Transition Program for Frail Older Adults | Interventions and/or outcomes do not meet criteria Non included main outcomes nor studied interventions. Nor RCT |
| McCann, Mark; Grundy, Emily; O'Reilly, Dermot | Why is housing tenure associated with a lower risk of admission to a nursing or residential home? Wealth, health and the incentive to keep 'my home' | Interventions and/or outcomes do not meet criteria Non included main outcomes nor studied interventions. Nor RCT |
| Garcia-Gollarte, Fermin; Baleriola-Julvez, Jose; Ferrero-Lopez, Isabel; Cruz-Jentoft, Alfonso J. | Inappropriate Drug Prescription at Nursing Home Admission | Interventions and/or outcomes do not meet criteria Non included main outcomes nor studied interventions. Nor RCT |
| Linertova, Renata; Garcia-Perez, Lidia; Ramon Vazquez-Diaz, Jose; Lorenzo-Riera, Antonio; Sarria-Santamera, Antonio | Interventions to reduce hospital readmissions in the elderly: in-hospital or home care. A systematic review | Interventions and/or outcomes do not meet criteria Non included main outcomes nor studied interventions. Nor RCT |
| Palmieri, Vittorio; Pezzullo, Salvatore; Lubrano, Vincenzo; Bettella, Stefania; Olandese, Mariarosaria; Sorrentino, Carmela; Russo, Cesare; Celentano, Aldo | Telemetry for the home control of arterial pressure, heart rate and oxygen saturation in heart failure: impact on hospitalization in a non-experimental context | Interventions and/or outcomes do not meet criteria Non included main outcomes nor studied interventions. Nor RCT |
| Zweig, Steven C.; Popejoy, Lori L.; Parker-Oliver, Debra; Meadows, Susan E. | The Physician's Role in Patients' Nursing Home Care "She's a Very Courageous and Lovely Woman. I Enjoy Caring for Her" | Interventions and/or outcomes do not meet criteria Non included main outcomes nor studied interventions. Nor RCT |
| Martin, Robert C. G.; Brown, Russell; Puffer, Lisa; Block, Stacey; Callender, Glenda; Quillo, Amy; Scoggins, Charles R.; McMasters, Kelly M. | Readmission Rates After Abdominal Surgery The Role of Surgeon, Primary Caregiver, Home Health, and Subacute Rehab | Interventions and/or outcomes do not meet criteria Non included main outcomes nor studied interventions. Nor RCT |
| Shu, Chin-Chung; Hsu, Nin-Chieh; Lin, Yu-Feng; Wang, Jann-Yuan; Lin, Jou-Wei; Ko, Wen-Je | Integrated postdischarge transitional care in a hospitalist system to improve discharge outcome: an experimental study | Interventions and/or outcomes do not meet criteria Non included main outcomes nor studied interventions. Nor RCT |
| Devon, Karen M.; Urbach, David R.; McLeod, Robin S. | Postoperative disposition and health services use in elderly patients undergoing colorectal cancer surgery: A population-based study | Interventions and/or outcomes do not meet criteria Non included main outcomes nor studied interventions. Nor RCT |
| Li, Yue; Cai, Xueya; Cram, Peter | Are Patients With Serious Mental Illness More Likely to be Admitted to Nursing Homes With More Deficiencies in Care? | Interventions and/or outcomes do not meet criteria Non included main outcomes nor studied interventions. Nor RCT |
| Herrera-Espineira, Carmen; Rodriguez del Aguila, Maria del Mar; Navarro Espigares, Jose Luis; Godoy Montijano, Amparo; Garcia Priego, Alfonso; Gomez Rodriguez, Javier; Reyes Sanchez, Isabel | Effect of a telephone care program after hospital discharge from a trauma surgery unit | Interventions and/or outcomes do not meet criteria Non included main outcomes nor studied interventions. Nor RCT |
| Ornstein, Katherine; Smith, Kristofer L.; Foer, Dinah Herlands; Lopez-Cantor, Maria Tereza; Soriano, Theresa | To the Hospital and Back Home Again: A Nurse Practitioner-Based Transitional Care Program for Hospitalized Homebound People | Interventions and/or outcomes do not meet criteria Non included main outcomes nor studied interventions. Nor RCT |
| Unroe, Kathleen T.; Greiner, Melissa A.; Hernandez, Adrian F.; Whellan, David J.; Kaul, Padma; Schulman, Kevin A.; Peterson, Eric D.; Curtis, Lesley H. | Resource Use in the Last 6 Months of Life Among Medicare Beneficiaries With Heart Failure, 2000-2007 | Interventions and/or outcomes do not meet criteria Non included main outcomes nor studied interventions. Nor RCT |
| Claudia Espinel-Bermudez, Maria; Sanchez-Garcia, Sergio; Juarez-Cedillo, Teresa; Juan Garcia-Gonzalez, Jose; Viveros-Perez, Alvaro; Garcia-Pena, Carmen | Impact of the program home care for the chronically ill for elderly: quality of life and hospital readmissions | Interventions and/or outcomes do not meet criteria Non included main outcomes nor studied interventions. Nor RCT |
| **Authors** | **Study title** | **Exclusion reason** |
| Sorknaes, Anne Dichmann; Madsen, Hanne; Hallas, Jesper; Jest, Peder; Hansen-Nord, Michael | Nurse tele-consultations with discharged COPD patients reduce early readmissions - an interventional study | Interventions and/or outcomes do not meet criteria Non included main outcomes nor studied interventions. Nor RCT |
| Kada, Olivia; Brunner, Eva; Likar, Rudolf; Pinter, Georg; Leutgeb, Ines; Francisci, Nina; Pfeiffer, Bettina; Janig, Herbert | From the nursing home to hospital and back again... a mixed methods study on hospital transfers from nursing homes | Interventions and/or outcomes do not meet criteria Non included main outcomes nor studied interventions. Nor RCT |
| Seow, Hsien; Barbera, Lisa; Howell, Doris; Dy, Sydney M. | How End-of-Life Home Care Services Are Used from Admission to Death: A population-based cohort study | Interventions and/or outcomes do not meet criteria Non included main outcomes nor studied interventions. Nor RCT |
| Bahrmann, A.; Abel, Al.; Specht-Leible, N.; Abel, Am.; Woerz, E.; Hoelscher, E.; Zieschang, T.; Oster, P.; Zeyfang, A. | Treatment quality in geriatric patients with diabetes mellitus in various home environments | Interventions and/or outcomes do not meet criteria Non included main outcomes nor studied interventions. Nor RCT |
| Ouslander, Joseph G. | A Crack in the System | Interventions and/or outcomes do not meet criteria Non included main outcomes nor studied interventions. Nor RCT |
| Pecchia, Leandro; Schiraldi, Fernando; Verde, Sossio; Mirante, E.; Bath, Peter A.; Bracale, Marcello | EVALUATION OF SHORT-TERM EFFECTIVENESS OF THE DISEASE MANAGEMENT PROGRAM "DI.PRO.DI." ON CONTINUITY OF CARE OF PATIENTS WITH CONGESTIVE HEART FAILURE | Interventions and/or outcomes do not meet criteria Non included main outcomes nor studied interventions. Nor RCT |
| Peng, Li-Ning; Liang, Chih-Kuang; Chou, Ming-Yueh; Lin, Ming-Hsien; Lai, Hsiu-Yun; Hwang, Shinn-Jang; Chen, Liang-Kung | Association between serum copper, zinc and hospital admissions among care home residents | Interventions and/or outcomes do not meet criteria Non included main outcomes nor studied interventions. Nor RCT |
| Bueno, Hector; Ross, Joseph S.; Wang, Yun; Chen, Jersey; Vidan, Maria T.; Normand, Sharon-Lise T.; Curtis, Jeptha P.; Drye, Elizabeth E.; Lichtman, Judith H.; Keenan, Patricia S.; Kosiborod, Mikhail; Krumholz, Harlan M. | Trends in Length of Stay and Short-term Outcomes Among Medicare Patients Hospitalized for Heart Failure, 1993-2006 | Interventions and/or outcomes do not meet criteria Non included main outcomes nor studied interventions. Nor RCT |
| Chen, Guanmin; Khan, Nadia; King, Kathryn M.; Hemmelgarn, Brenda R.; Quan, Hude | Home care utilization and outcomes among Asian and other Canadian patients with heart failure | Interventions and/or outcomes do not meet criteria Non included main outcomes nor studied interventions. Nor RCT |
| Temple, April; Andel, Ross; Dobbs, Debra | Setting of care modifies risk of nursing home placement for older adults with dementia | Interventions and/or outcomes do not meet criteria Non included main outcomes nor studied interventions. Nor RCT |
| Perner, A.; Goetze, H.; Stuhr, C.; Braehler, E. | Palliative home care of cancer patients in the Leipzig region | Interventions and/or outcomes do not meet criteria Non included main outcomes nor studied interventions. Nor RCT |
| Sankaran, Shankar; Kenealy, Tim; Adair, Allan; Adair, Vivienne; Coster, Heather; Whitehead, Noeline; Sheridan, Nicolette; Parsons, Matthew; Marshall, Elaine; Bailey, Leslie; Price, Catherine; Crombie, Dwayne; Rea, Harry | A complex intervention to support 'rest home' care: a pilot study | Interventions and/or outcomes do not meet criteria Non included main outcomes nor studied interventions. Nor RCT |
| Van Rensbergen, Gilberte; Nawrot, Tim | Medical Conditions of Nursing Home Admissions | Interventions and/or outcomes do not meet criteria Non included main outcomes nor studied interventions. Nor RCT |
| Xu, Huiping; Weiner, Michael; Paul, Sudeshna; Thomas, Joseph, III; Craig, Bruce; Rosenman, Marc; Doebbeling, Caroline Carney; Sands, Laura P. | Volume of Home- and Community-Based Medicaid Waiver Services and Risk of Hospital Admissions | Interventions and/or outcomes do not meet criteria Non included main outcomes nor studied interventions. Nor RCT |
| **Authors** | **Study title** | **Exclusion reason** |
| Onder, Graziano; Lipeyoti, Rosa; Soldato, Manuel; Cipriani, Maria Camilla; Bernabei, Roberto; Landi, Francesco | Depression and risk of nursing home admission among older adults in home care in Europe: Results from the aged in home care (AdHOC) study | Interventions and/or outcomes do not meet criteria Non included main outcomes nor studied interventions. Nor RCT |
| Garcia-Altes, Anna; Borrell, Carme; Cote, Louis; Plaza, Aina; Benet, Josep; Guarga, Alex | Measuring the performance of urban healthcare services: results of an international experience | Interventions and/or outcomes do not meet criteria Non included main outcomes nor studied interventions. Nor RCT |
| Garasen, Helge; Johnsen, Roar | The quality of communication about older patients between hospital physicians and general practitioners: A panel study assessment | Interventions and/or outcomes do not meet criteria Non included main outcomes nor studied interventions. Nor RCT |
| Foroni, Micaela; Salvioli, Gianfranco; Rielli, Rita; Goldoni, Carlo Alberto; Orlandi, Giuliano; Sajani, Stefano Zauli; Guerzoni, Andrea; Maccaferri, Cristina; Daya, Ghassan; Mussi, Chiara | A retrospective study on heat-related mortality in an elderly population during the 2003 heat wave in Modena, Italy: The Argento Project | Interventions and/or outcomes do not meet criteria Non included main outcomes nor studied interventions. Nor RCT |
| Muramatsu, Naoko; Yin, Hongjun; Campbell, Richard T.; Hoyem, Ruby L.; Jacob, Martha A.; Ross, Christopher O. | Risk of nursing home admission among older americans: Does states' spending on home- and community-based services matter? | Interventions and/or outcomes do not meet criteria Non included main outcomes nor studied interventions. Nor RCT |
| Guo, Lin; Chung, Eugene S.; Casey, Donald E., Jr.; Snow, Richard | Redefining hospital readmissions to better reflect clinical course of care for heart failure patients | Interventions and/or outcomes do not meet criteria Non included main outcomes nor studied interventions. Nor RCT |
| Kind, Amy J. H.; Smith, Maureen A.; Frytak, Jennifer R.; Finch, Michael D. | Bouncing back: Patterns and predictors of complicated transitions 30 days after hospitalization for acute ischemic stroke | Interventions and/or outcomes do not meet criteria Non included main outcomes nor studied interventions. Nor RCT |
| Onder, Graziano; Liperoti, Rosa; Soldato, Manuel; Carpenter, Iain; Steel, Knight; Bernabei, Roberto; Landi, Francesco | Case management and risk of nursing home admission for older adults in home care: Results of the AgeD in HOme Care Study | Interventions and/or outcomes do not meet criteria Non included main outcomes nor studied interventions. Nor RCT |
| Stearns, Sally C.; Dalton, Kathleen; Holmes, George M.; Seagrave, Susanne M. | Using propensity stratification to compare patient outcomes in hospital-based versus freestanding skilled-nursing facilities | Interventions and/or outcomes do not meet criteria Non included main outcomes nor studied interventions. Nor RCT |
| Dengler, I.; Leukel, N.; Meuser, T.; Jost, W. H. | Prospective study of the direct and indirect costs of idiopathic Parkinson's disease | Interventions and/or outcomes do not meet criteria Non included main outcomes nor studied interventions. Nor RCT |
| Friedman, Susan M.; Steinwachs, Donald M.; Temkin-Greener, Helena; Mukamel, Dana B. | Informal caregivers and the risk of nursing home admission among individuals enrolled in the program of all-inclusive care for the elderly | Interventions and/or outcomes do not meet criteria Non included main outcomes nor studied interventions. Nor RCT |
| Mitchell, Glenn, II; Salmon, Jennifer R.; Polivka, Larry; Soberon-Ferrer, Horacio | The relative benefits and cost of medicaid home- and community-based services in Florida | Interventions and/or outcomes do not meet criteria Non included main outcomes nor studied interventions. Nor RCT |
| Elkins, Jacob S.; Whitmer, Rachel A.; Sidney, Stephen; Sorel, Mike; Yaffe, Kristine; Johnston, S. Claiborne | Midlife obesity and long-term risk of nursing home admission | Interventions and/or outcomes do not meet criteria Non included main outcomes nor studied interventions. Nor RCT |
| Rosenberg, Reena; Vinker, Shlorno; Yaphe, John; Nakar, Sasson | The role of periodic mortality case review sessions in a primary care teaching clinic | Interventions and/or outcomes do not meet criteria Non included main outcomes nor studied interventions. Nor RCT |
| Schofield, Irene; Knussen, Christina; Tolson, Debbie | A mixed method study to compare use and experience of hospital care and a nurse-led acute respiratory assessment service offering home care to people with an acute exacerbation of Chronic Obstructive Pulmonary Disease | Interventions and/or outcomes do not meet criteria Non included main outcomes nor studied interventions. Nor RCT |
| **Authors** | **Study title** | **Exclusion reason** |
| van Bilsen, PMA; Hamers, JPH; Groot, W; Spreeuwenberg, C | Demand of elderly people for residential care: an exploratory study | Interventions and/or outcomes do not meet criteria Non included main outcomes nor studied interventions. Nor RCT |
| Ara, J; Estrada, O; Riera, C; Bonet, G; Cuxart, A; Romero, R | The usefulness of hospital at home in nephrology | Interventions and/or outcomes do not meet criteria Non included main outcomes nor studied interventions. Nor RCT |
| Oura, A; Washio, M; Wada, J; Arai, Y; Mori, M | Factors related to institutionalization among the frail elderly with home-visiting nursing service in Japan | Interventions and/or outcomes do not meet criteria Non included main outcomes nor studied interventions. Nor RCT |
| Martinez, Andres; Everss, Estrella; Luis Rojo-Alvarez, Jose; Pascual Figal, Domingo; Garcia-Alberola, Arcadio | A systematic review of the literature on home monitoring for patients with heart failure | Interventions and/or outcomes do not meet criteria Non included main outcomes nor studied interventions. Nor RCT |
| Kales, HC; Chen, PJ; Blow, FC; Welsh, DE; Mellow, AM | Rates of clinical depression diagnosis, functional impairment, and nursing home placement in coexisting dementia and depression | Interventions and/or outcomes do not meet criteria Non included main outcomes nor studied interventions. Nor RCT |
| Anderson, MA; Clarke, MM; Helms, LB; Foreman, MD | Hospital readmission from home health care before and after prospective payment | Interventions and/or outcomes do not meet criteria Non included main outcomes nor studied interventions. Nor RCT |
| Saynajakangas, O; Kinnunen, T; Tuuponen, T; Keistinen, T | Length of stay and interval to readmission in emergency hospital treatment of COPD | Interventions and/or outcomes do not meet criteria Non included main outcomes nor studied interventions. Nor RCT |
| Li, H; Morrow-Howell, N; Proctor, EK | Post-acute home care and hospital readmission of elderly patients with congestive heart failure | Interventions and/or outcomes do not meet criteria Non included main outcomes nor studied interventions. Nor RCT |
| Landi, F; Onder, G; Cesari, M; Barillaro, C; Lattanzio, F; Carbonin, PU; Bernabei, R | Comorbidity and social factors predicted hospitalization in frail elderly patients | Interventions and/or outcomes do not meet criteria Non included main outcomes nor studied interventions. Nor RCT |
| de Zuazu, HMR; de los Cobos, JR; Basurto, EA; Ruiz, JMC; Borau, FA; Eraso, PL | Treatment of congestive heart failure in the setting of hospital at home. Study of 158 patients | Interventions and/or outcomes do not meet criteria Non included main outcomes nor studied interventions. Nor RCT |
| Anttila, SK; Huhtala, HS; Pekurinen, MJ; Pitkajarvi, TK | Cost-effectiveness of an innovative four-year post-discharge programme for elderly patients - Prospective follow-up of hospital and nursing home use in project elderly and randomized controls | Interventions and/or outcomes do not meet criteria Non included main outcomes nor studied interventions. Nor RCT |
| Bergman, H; Beland, F | Evaluating innovation in the care of Canada's frail elderly population | Interventions and/or outcomes do not meet criteria Non included main outcomes nor studied interventions. Nor RCT |
| Kongensgaard R, Hansen TK, Krogseth M, Gregersen M. | Impact of involvement of relatives in early home visits by a hospital-led geriatric team | Non RCT |
| Tierney B, Melby V, Todd S. Service evaluation comparing Acute Care at Home for older people service and conventional service within an acute hospital care of elderly ward | Service evaluation comparing Acute Care at Home for older people service and conventional service within an acute hospital care of elderly ward | Non RCT |
| **Authors** | **Study title** | **Exclusion reason** |
| Ma C, McDonald MV, Feldman PH, Miner S, Jones S, Squires A. | Continuity of Nursing Care in Home Health: Impact on Rehospitalization Among Older Adults With Dementia | Non RCT |
| Carter J, Hassan S, Walton A, Yu L, Donelan K, Thorndike AN. | Effect of Community Health Workers on 30-Day Hospital Readmissions in an Accountable Care Organization Population: A Randomized Clinical Trial | Non frailty patients nor considerer as a frailty patients |
| Takahashi PY, Chandra A, McCoy RG, Borkenhagen LS, Larson ME, Thorsteinsdottir B, Hickman JA, Swanson KM, Hanson GJ, Naessens JM. | Outcomes of a Nursing Home-to-Community Care Transition Program | Non RCT |
| Snoek JA, Prescott EI, van der Velde AE, Eijsvogels TMH, Mikkelsen N, Prins LF, Bruins W, Meindersma E, González-Juanatey JR, Peña-Gil C, González-Salvado V, Moatemri F, Iliou MC, Marcin T, Eser P, Wilhelm M, Van't Hof AWJ, de Kluiver EP. | Effectiveness of Home-Based Mobile Guided Cardiac Rehabilitation as Alternative Strategy for Nonparticipation in Clinic-Based Cardiac Rehabilitation Among Elderly Patients in Europe: A Randomized Clinical Trial | Non frailty patients nor considerer as a frailty patients |
| Viberg B, Erlandsen Claville LU, Andersen LR, Fredholm L, Dall-Hansen D, Grejsen H. | Standardized, Coordinated Care in Nursing Homes Lowers Rehospitalization After Hip Fracture | Interventions and/or outcomes do not meet criteria Non included main outcomes nor studied interventions. Nor RCT |
| Freeman CR, Scott IA, Hemming K, Connelly LB, Kirkpatrick CM, Coombes I, Whitty J, Martin J, Cottrell N, Sturman N, Russell GM, Williams I, Nicholson C, Kirsa S, Foot H. | Reducing Medical Admissions and Presentations Into Hospital through Optimising Medicines (REMAIN HOME): a stepped wedge, cluster randomised controlled trial | Interventions and/or outcomes do not meet criteria Non included main outcomes nor studied interventions. |
| Cassarino M, Robinson K, Trépel D, O'Shaughnessy Í, Smalle E, White S, Devlin C, Quinn R, Boland F, Ward ME, McNamara R, Steed F, O'Connor M, O'Regan A, McCarthy G, Ryan D, Galvin R. | Impact of assessment and intervention by a health and social care professional team in the emergency department on the quality, safety, and clinical effectiveness of care for older adults: A randomised controlled trial | Interventions and/or outcomes do not meet criteria Non included main outcomes nor studied interventions. |
| Castier S et al | Surviving COVID-19 After Hospital Discharge: Symptom, Functional, and Adverse Outcomes of Home Health Recipients | Non RCT. Non frailty patients nor considerer as a frailty patients |
| Buller LT et al | Association between anticholinergic burden and hospital readmission in older patients: a prospective analysis | Non RCT |
| Morrow C et al | Safety of Same and Next Day Discharge Following Revision Hip and Knee Arthroplasty Using Modern Perioperative Protocols | Non RCT. Non frailty patients nor considerer as a frailty patients |
| Robert B et al | Patient Characteristics Are Not Associated With Documentation of Weight and Heart Failure Related Sign and Symptom Assessment in Skilled Nursing Facilities | Non RCT. Non frailty patients nor considerer as a frailty patients |
| Robert B et al | A Case-Control Study of the Sub-Acute Care for Frail Elderly (SAFE) Unit on Hospital Readmission, Emergency Department Visits and Continuity of Post-Discharge Care | Non RCT |
| Freeman CR | Risk factors of readmission after geriatric hospital care: An interRAI-based cohort study in Finland | Non RCT |
| Schapira M | Reducing Medical Admissions and Presentations Into Hospital through Optimising Medicines (REMAIN HOME): a stepped wedge, cluster randomised controlled trial | Interventions and/or outcomes do not meet criteria Non included main outcomes nor studied interventions. |
| **Authors** | **Study title** | **Exclusion reason** |
| Zhong H, Poeran J, Liu J, Wilson LA, Memtsoudis SG. | Hip fracture characteristics and outcomes during COVID-19: a large retrospective national database review | Non RCT |
| Blackburn J., Balio C.P., Carnahan J.L., Fowler N.R., Hickman S.E., Sachs G.A., Tu W., Unroe K.T. | Facility and resident characteristics associated with variation in nursing home transfers: evidence from the OPTIMISTIC demonstration project | Non RCT. Non frailty patients nor considerer as a frailty patients |
| Reistetter T.A., Eschbach K., Prochaska J., Jupiter D.C., Hong I., Haas A.M., Ottenbacher K.J. | Understanding Variation in Postacute Care: Developing Rehabilitation Service Areas Through Geographic Mapping | Non RCT. Non frailty patients nor considerer as a frailty patients |
| Kerminen H.M., Jäntti P.O., Valvanne J.N.A., Huhtala H.S.A., Jämsen E.R.K. | Risk factors of readmission after geriatric hospital care: An interRAI-based cohort study in Finland | Non RCT |
| Castier S., Preda C., Puisieux F., Beuscart J.-B., Mahmoudi R., Visade F. | Association between anticholinergic burden and hospital readmission in older patients: a prospective analysis | Non RCT |
| Golnari P., Nazari P., Ansari S.A., Hurley M.C., Shaibani A., Potts M.B., Jahromi B.S. | Endovascular thrombectomy after large-vessel ischemic stroke: Utilization, outcomes, and readmissions across the United States | Non RCT. Non frailty patients nor considerer as a frailty patients |
| Bowles K.H., McDonald M., Barrón Y., Kennedy E., O'Connor M., Mikkelsen M. | Surviving COVID-19 After Hospital Discharge: Symptom, Functional, and Adverse Outcomes of Home Health Recipients | Non RCT. Non frailty patients nor considerer as a frailty patients |
| Kheir M., Saleem F., Wang C., Mann A., Chua J. | Higher albumin levels on admission predict better prognosis in patients with confirmed COVID-19 | Non RCT. Non frailty patients nor considerer as a frailty patients |
| Guzman-Clark J., Farmer M.M., Wakefield B.J., Viernes B., Yefimova M., Lee M.L., Hahn T.J. | Why patients stop using their home telehealth technologies over time: Predictors of discontinuation in Veterans with heart failure | Non RCT. Non frailty patients nor considerer as a frailty patients |
| Sanchis J., Sastre C., Ruescas A., Ruiz V., Valero E., Bonanad C., García-Blas S., Fernández-Cisnal A., González J., Miñana G., Núñez J. | Randomized Comparison of Exercise Intervention Versus Usual Care in Older Adult Patients with Frailty After Acute Myocardial Infarction | Interventions and/or outcomes do not meet criteria Non included main outcomes nor studied interventions. |
| Mont M.A., Cool C., Gregory D., Coppolecchia A., Sodhi N., Jacofsky D.J. | Health care utilization and payer cost analysis of robotic arm assisted total knee arthroplasty at 30, 60, and 90 days | Non RCT |
| Borgen I., Romney M.C., Redwood N., Delgado B., Alea P., George B.H., Puzziferro J., Shihabuddin L. | From Hospital to Home: An Intensive Transitional Care Management Intervention for Patients with COVID-19 | Interventions and/or outcomes do not meet criteria Non included main outcomes nor studied interventions. Non frailty patients |
| Simo N., Cesari M., Tchiero H., Rolland Y., De Souto Barreto P., Dartigues J.F., Vellas B., Tabue-Teguo M. | Frailty Index, Hospital Admission and Number of Days Spent in Hospital in Nursing Home Residents: Results from the INCUR Study | Interventions and/or outcomes do not meet criteria Non included main outcomes nor studied interventions. |
| Ryan O.F., Riley M., Cadilhac D.A., Andrew N.E., Breen S., Paice K., Shehata S., Sundararajan V., Lannin N.A., Kim J., Kilkenny M.F. | Factors Associated with Stroke Coding Quality: A Comparison of Registry and Administrative Data | Non RCT |
| Leahy A., O'Connor M., Condon J., Heywood S., Shanahan E., Peters C., Galvin R. | Diagnostic and predictive accuracy of the Clinical Frailty Scale among hospitalised older medical patients: A systematic review and meta-analysis protocol | Non RCT |
| Gray M.T., Hidden K.A., Malik A.T., Khan S.N., Phieffer L., Ly T.V., Quatman C.E. | Octogenarian and Nonagenarians Are at a Higher Risk for Experiencing Adverse 30-Day Outcomes Following ORIF of Ankle Fractures | Non RCT. Non frailty patients nor considerer as a frailty patients |
| **Authors** | **Study title** | **Exclusion reason** |
| Vignatelli L., Zenesini C., Belotti L.M.B., Baldin E., Bonavina G., Calandra-Buonaura G., Cortelli P., Descovich C., Fabbri G., Giannini G., Guarino M., Pantieri R., Samoggia G., Scaglione C., Trombetti S., D'Alessandro R., Nonino F., ParkLink Bologna group | Risk of Hospitalization and Death for COVID-19 in People with Parkinson's Disease or Parkinsonism | Non RCT. Non frailty patients nor considerer as a frailty patients |
| Drake S.A., Conway S.H., Yang Y., Cheatham L.S., Wolf D.A., Adams S.D., Wade C.E., Holcomb J.B. | When falls become fatal—Clinical care sequence | Non RCT. Interventions and/or outcomes do not meet criteria Non included main outcomes nor studied interventions. |
| Bosetti A., Gayot C., Preux P.-M., Tchalla A. | Effectiveness of a Geriatric Emergency Medicine Unit for the Management of Neurocognitive Disorders in Older Patients: Results of the MUPACog Study | Non RCT. Interventions and/or outcomes do not meet criteria Non included main outcomes nor studied interventions. |
| Vogelsmeier A., Popejoy L., Canada K., Galambos C., Petroski G., Crecelius C., Alexander G.L., Rantz M. | Results of the Missouri Quality Initiative in Sustaining Changes in Nursing Home Care: Six-Year Trends of Reducing Hospitalizations of Nursing Home Residents | Non RCT. Interventions and/or outcomes do not meet criteria Non included main outcomes nor studied interventions. |
| Candelaresi P., Manzo V., Servillo G., Muto M., Barone P., Napoletano R., Saponiero R., Andreone V., Palma V., Spitaleri D., D'Onofrio F., Maniscalco G., Salvatore S., Leone G., Capone E., Schettino C., Romano D., Martusciello G., Miniello S., Mazzaferro M.P., Ascione S. | The Impact of Covid-19 Lockdown on Stroke Admissions and Treatments in Campania | Non RCT. Interventions and/or outcomes do not meet criteria Non included main outcomes nor studied interventions. |
| Gallioli A., Albo G., Lievore E., Boeri L., Longo F., Spinelli M.G., Costantino G., Montanari E., De Lorenzis E. | How the COVID-19 Wave Changed Emergency Urology: Results From an Academic Tertiary Referral Hospital in the Epicentre of the Italian Red Zone | Non RCT. Interventions and/or outcomes do not meet criteria Non included main outcomes nor studied interventions. |
| Buller L.T., Hubbard T.A., Ziemba-Davis M., Deckard E.R., Meneghini R.M. | Safety of Same and Next Day Discharge Following Revision Hip and Knee Arthroplasty Using Modern Perioperative Protocols | Non RCT. Interventions and/or outcomes do not meet criteria Non included main outcomes nor studied interventions. |
| Liu D.S., Cheng C., Islam R., Tacey M., Sidhu A., Lam D., Strugnell N. | Prophylactic Negative-pressure Dressings Reduce Wound Complications and Resource Burden After Emergency Laparotomies | Non RCT. Interventions and/or outcomes do not meet criteria Non included main outcomes nor studied interventions. |
| Albo Z., Marino J., Nagy M., Jayaraman D.K., Azeem M.U., Puri A.S., Henninger N. | Relationship of white matter lesion severity with early and late outcomes after mechanical thrombectomy for large vessel stroke | Non RCT. Interventions and/or outcomes do not meet criteria Non included main outcomes nor studied interventions. |
| Sorensen, Andrea; Grotts, Jonathan F.; Tseng, Chi‐Hong; Moreno, Gerardo; Maranon, Richard; Whitmire, Natalie; Viramontes, Omar; Atkins, Sandy; Sefilyan, Ester; Simmons, June W.; Mangione, Carol M. | A Collaboration Among Primary Care–Based Clinical Pharmacists and Community‐Based Health Coaches. | Non RCT |
| Chovanec, Kelli A.; Arsene, Camelia; Beck, Amanda; Zachrich, Kristen; Liedel, Bethany; Wolff-Elliott, Jaclyn | Association of Discharge Disposition with Outcomes. | Non RCT. Interventions and/or outcomes do not meet criteria Non included main outcomes nor studied interventions. |
| Speck, Patricia M.; Baker, Natalie R. | Case Series for the LNC: Assessing Risk for Elder Abuse Using DMES©. | Non RCT. Interventions and/or outcomes do not meet criteria Non included main outcomes nor studied interventions. |
| Fuhrmann, Ana Cláudia; Bierhals, Carla Cristiane Becker Kottwitz; dos Santos, Naiana Oliveira; de Oliveira Machado, Diani; Peixoto Cordova, Fernanda; Girardi Paskulin, Lisiane Manganelli | Construction and validation of an educational manual for family caregivers of older adults after a stroke. | Non RCT. Interventions and/or outcomes do not meet criteria Non included main outcomes nor studied interventions. |
| **Authors** | **Study title** | **Exclusion reason** |
| Mayr, Florian B.; Plowman, Judith L.; Blakowski, Sandra; Sell-Shemansky, Kimberly; Young, Joleene M.; Yende, Sachin | Feasibility of a Home-Based Palliative Care Intervention for Elderly Multimorbid Survivors of Critical Illness. | Non RCT. Interventions and/or outcomes do not meet criteria Non included main outcomes nor studied interventions. |
| Chimenti, Chris; Sears, Geralyn; McIntyre, Jack | Sepsis in Home Health Care: Screening, Education, and Rapid Triage. | Non RCT. Interventions and/or outcomes do not meet criteria Non included main outcomes nor studied interventions. |
| Shah, Neeraj M.; Kaltsakas, Georgios | Telemedicine in the management of patients with chronic respiratory failure. | Non RCT. Interventions and/or outcomes do not meet criteria Non included main outcomes nor studied interventions. |
| M. Tzuang; J. T. Owusu; J. Huang; O. C. Sheehan; G. W. Rebok; M. L. Paudel; E. M. Wickwire; J. D. Kasper; A. P. Spira | Associations of insomnia symptoms with subsequent health services use among community-dwelling US older adults | Non RCT. Interventions and/or outcomes do not meet criteria Non included main outcomes nor studied interventions |
| Ma C, McDonald MV, Feldman PH, Miner S, Jones S, Squires A. | Continuity of Nursing Care in Home Health: Impact on Rehospitalization Among Older Adults With Dementia | Non RCT |
| Han CY, Sharma Y, Yaxley A, Baldwin C, Woodman R, Miller M.Clin Interv Aging. 2023 May 17;18:809-825. | Individualized Hospital to Home, Exercise-Nutrition Self-Managed Intervention for Pre-Frail and Frail Hospitalized Older Adults: The INDEPENDENCE Randomized Controlled Pilot Trial. | RCT pilot. Other reasons |
| Khajehpoor MH, Shahrbabaki PM, Nouhi E. BMC Palliat Care. 2023 Sep 6;22(1):130. | Effects of a home-based palliative heart failure program on quality of life among the elderly: a clinical trial study. | Interventions and/or outcomes do not meet criteria. Non frailty patients |

List of excluded studies and reasons

| **Excluded studies via other methods** | | |
| --- | --- | --- |
| **Authors** | **Study title** | **Exclusion reason** |
| *Cameron ID, Fairhall N, Langron C, Lockwood K, Monaghan N, Aggar C, et al.* | *A multifactorial interdisciplinary intervention reduces frailty in older people: Randomized trial.* | Interventions and/or outcomes do not meet criteria Non included main outcomes nor studied interventions |
| *Ebrahimi Z, Eklund K, Dahlin-Ivanoff S, Jakobsson A, Wilhelmson K.* | *Effects of a continuum of care intervention on frail elders’ self-rated health, experiences of security/safety and symptoms: A randomised controlled trial.* | Interventions and/or outcomes do not meet criteria Non included main outcomes nor studied interventions |
| Muntinga ME, Hoogendijk EO, van Leeuwen KM, van Hout HP, Twisk JW, van der Horst HE, et al. | Implementing the chronic care model for frail older adults in the Netherlands: study protocol of ACT (frail older adults: care in transition). | Other reasons |
| Kono A, Izumi K, Yooshiyuki N, Kanaya Y, Rubenstein LZ. | Effects of an updated preventive home visit program based on a systematic structured assessment of care needs for ambulatory frail older adults in Japan: A randomized controlled trial. | Interventions and/or outcomes do not meet criteria Non included main outcomes nor studied interventions |
| Metzelthin SF, van Rossum E, de Witte LP, Ambergen AW, Hobma SO, Sipers W, et al. | Effectiveness of interdisciplinary primary care approach to reduce disability in community dwelling frail older people: Cluster randomised controlled trial. | Interventions and/or outcomes do not meet criteria Non included main outcomes nor studied interventions |
| **Authors** | **Study title** | **Exclusion reason** |
| Parsons M, Senior H, Kerse N, Chen MH, Jacobs S, Vanderhoorn S, et al. | Should care managers for older adults be located in primary care? A randomized controlled trial. | Other reasons |
| Granbom M, Kristensson J, Sandberg M. | Effects on leisure activities and social participation of a case management intervention for frail older people living at home: a randomised controlled trial. | Interventions and/or outcomes do not meet criteria Non included main outcomes nor studied interventions |
| Andrei CL, Sinescu CJ, Ianula RM, Popa AI, Chioncel VP, Mischie AN, Adam FC, Dasoveanu M, Mincu DE, Grigorean VT. | Can be the home care of the heart failure patients a better economic alternative? European | Other reasons |
| Caplan GA, Coconis J, Woods J. | Effect of hospital in the home treatment on physical and cognitive function: a randomized controlled trial. Journal of Gerontology | Interventions and/or outcomes do not meet criteria Non included main outcomes nor studied interventions. Other reasons. Non frailty patients |
| Echevarria C, Gray J, Hartley T, Steer J, Miller J, Simpson AJ, et al. | Home treatment of COPD exacerbation selected by DECAF score: a non-inferiority, randomised controlledtrial and economic evaluation.] | Interventions and/or outcomes do not meet criteria Non included main outcomes nor studied interventions. Other reasons. Non frailty patients |
| Harris R, Ashton T, Broad J, Connolly G, Richmond D. | The effectiveness, acceptability and costs of a hospital-at-home service compared with acute hospital care: a randomised controlled trial. | Other reasons. Non frailty patients |
| Levine DM, Ouchi K, Blanchfield B, Saenz A, Burke K, Paz M, et al. | Hospital-level care at home for acutely ill adults: a randomized controlled trial. | Other reasons. Non frailty patients |
| Mendoza H, Martin MJ, Garcia A, Aros F, Aizpuru F, Regalado de Los Cobos J et al. | Hospital at home care model as an effective alternative in the management of decompensated chronic heart failure. | Other reasons. Non frailty patients |
| Ricauda NA, Tibaldi V, Leff B, Scarafiotti C, Marinello R, Zanocchi M et al. | Substitutive hospital at home versus inpatient care for elderly patients with exacerbations of chronic obstructive pulmonary disease: a prospective randomised controlled trial. | Other reasons. Non frailty patients |
| Tibaldi V, Isaia G, Scarafiotti C, Gariglio F, Zanocchi M, Bo M et al. | Hospital at home for elderly patients with acute decompensation of chronic heart failure: a prospective randomized controlled trial. | Interventions and/or outcomes do not meet criteria Non included main outcomes nor studied interventions. |
| Wilson A, Parker H, Wynn A, Jagger C, Spiers N, Jones J et al. | Randomised controlled trial of effectiveness of Leicester hospital at home scheme compared with hospital care. | Other reasons. Non frailty patients |
| Anderson C, Rubenach S, Mhurchu CN, Clark M, Spencer C, Winsor A. | Home or hospital for stroke rehabilitation? Results of a randomized controlled trial: I: health outcomes at 6 months. | Other reasons. Non frailty patients |
| Askim T, Rohweder G, Lydersen S, Indredavik B. | Evaluation of an extended stroke unit service with early supported discharge for patients living in a rural community. A randomized controlled trial. | Other reasons. Non frailty patients |
| Díaz Lobatoa S, González Lorenzo F, Gómez Mendieta MA, Mayoralas Alises S, Martín Arechabala I, Villasante Fernández-Montes C. | Evaluation of a home hospitalisation program in patients with COPD exacerbations [Evaluación de un programa de hospitalización domiciliaria en pacientes con EPOC agudizada]. | Interventions and/or outcomes do not meet criteria Non included main outcomes nor studied interventions. Non frailty patients |
| **Authors** | **Study title** | **Exclusion reason** |
| Donald IP, Baldwin RN, Bannerjee M. | Gloucester hospital-at-home: a randomized controlled trial. | Other reasons. Not retrieved |
| Donnelly M, Power M, Russell M, Fullerton K. | Randomised controlled trial of an early discharge rehabilitation service: the Belfast Community Stroke Trial. | Other reasons. Non frailty patients |
| Indredavik B, Bakke F, Slordahl SA, Rokseth R, Haheim LL. | Benefit of an extended stroke unit service with early supported discharge: A randomized, controlled trial. | Other reasons. Non frailty patients |
| Karlsson A, Berggren M, Gustafson Y, B Olofsson, Lindelöf N, Stenvall M... | Effects of geriatric interdisciplinary home rehabilitation on walking ability and length of hospital stay after hip fracture: A randomized controlled trial | Other reasons. Non frailty patients |
| Martin F, Oyewole A, Moloney A. | A randomized controlled trial of a high support hospital discharge team for elderly people. | Other reasons. Not retrieved. Non frailty patients |
| Mayo NE, Wood-Dauphinee S, Cote R, Gayton D, Carlton J, Buttery J, et al. | There's no place like home: an evaluation of early supported discharge for stroke. | Interventions and/or outcomes do not meet criteria Non included main outcomes nor studied interventions. Non frailty patients |
| Palmer Hill S, Flynn J, Crawford EJP. | Early discharge following total knee replacement - a trial of patient satisfaction and outcomes using an orthopaedic outreach team. | Interventions and/or outcomes do not meet criteria Non included main outcomes nor studied interventions. Non frailty patients |
| Richards SH, Coast J, Gunnell DJ, Peters TJ, Pounsford J, Darlow MA. | Randomised controlled trial comparing effectiveness and acceptability of an early discharge, hospital at home scheme with acute hospital care | Interventions and/or outcomes do not meet criteria Non included main outcomes nor studied interventions. Non frailty patients |
| Rudd AG, Wolfe CD, Tilling K, Beech R. | Randomised controlled trial to evaluate early discharge scheme for patients with stroke. | Non frailty elderly patients |
| Skwarska E, Cohen G, Skwarski KM, Lamb C, Bushell D, Parker S, et al.. | Randomised controlled trial of supported discharge in patients with exacerbations of chronic obstructive pulmonary disease | Non frailty elderly patients |
| Suwanwela NC, Phanthumchinda K, Limtongkul S, Suvanprakorn P. | Thai Red Cross Volunteers Bureau. Comparison of short (3-day) hospitalization followed by home care treatment and conventional (10-day) hospitalization for acute ischemic stroke. | Non frailty elderly patients |
| Tibaldi V, Isaia G, Bergerone S, Moiraghi C, Gariglio F, Marchetto C, et al. | A randomized clinical trial on the efficacy of an early discharge to a hospital at home service of elderly patients with acute decompensation of severe chronic heart failure [Studio clinico randomizzato sull’efficacia della dimissione precoce in ospedalizzazione a domicilio di pazienti anzianicon scompenso cardiaco cronico avanzato riacutizzato]. | Other reasons. Not retrieved |
| Utens CM, Goossens LM, Smeenk FW, Rutten-van Mölken MP, Van Vliet M, Braken MW, et al. | Early assisted discharge with generic community nursing for chronic obstructive pulmonary disease exacerbations: Results of a randomised controlled trial. | Non frailty elderly patients |
| Widén Holmqvist L, Von Koch L, Kostulas V, Holm M, Widsell G, Tegler H, et al. | A randomized controlled trial of rehabilitation at home after stroke in southwest Stockholm. | Non frailty elderly patients |
| **Authors** | **Study title** | **Exclusion reason** |
| Taube E, Kristensson J, Midlöv P, Jakobsson U. | The use of case management for community‐dwelling older people: the effects on loneliness, symptoms of depression and life satisfaction in a randomised controlled trial. | Interventions and/or outcomes do not meet criteria Non included main outcomes nor studied interventions. Non frailty patients |
| Caplan GA, Ward JA, Brennan NJ, Coconis J, Board N, Brown A. | Hospital in the home: a randomised controlled trial. | Interventions and/or outcomes do not meet criteria |
| Davies L, Wilkinson M, Bonner S, Calverley PM, Angus RM. | Hospital at home versus hospital care in patients with exacerbations of chronic obstructive pulmonary disease: prospective randomised controlled trial. | Interventions and/or outcomes do not meet criteria Non included main outcomes nor studied interventions. Non frailty patients |
| Ricauda NA, Tibaldi V, Leff B, Scarafiotti C, Marinello R, Zanocchi M et al. | Substitutive hospital at home versus inpatient care for elderly patients with exacerbations of chronic obstructive pulmonary disease: a prospective randomised controlled trial. | Non frailty elderly patients |
| Cunliffe A, Gladman JRF, Husbands SL, Miller P, Dewey ME, Harwood RH. | Sooner and healthier: a randomised controlled trial and interview study of an early discharge rehabilitation service for older people. | Interventions and/or outcomes do not meet criteria Non included main outcomes nor studied interventions. |
| Hoogendijk EO, van der Horst HE, van de Ven PM, Twisk JWR, Deeg DJH, Frijters DHM, et al. | Effectiveness of a Geriatric Care Model for frail older adults in primary care: Results from a stepped wedge cluster randomized trial. European Journal of Internal Medicine | Interventions and/or outcomes do not meet criteria. |
| Suijker JJ, van Rijn M, Buurman BM, Ter Riet G, Moll van Charante EP, de Rooij SE. | Effects of nurse-led multifactorial care to prevent disability in community-living older people: cluster randomized trial. | Non frailty elderly patients included |
| Fordyce et al., | Senior Team Assessment and Referral Program-STAR | Non frailty elderly patients inlcuded |
| Melin et al., | Efficacy of the Rehabilitation of Elderly Primary Health Care Patients After Short-Stay Hospital Treatment | Non frailty elderly patients included |

|  |
| --- |

| **Table S5.** Frailty definition per study | |
| --- | --- |
| **Study** | **Frailty definition / Frailty criteria inclusion** |
| ***Bernabei et al., 1998*** | No frailty criteria measure. People aged 65 and over who were recipients of home health services or home assistance programmes. Usually, patients were receiving these services because of multiple geriatric conditions (for example, dementia, immobility, incontinence, and stroke deficits). |
| ***Gagnon et al., 1999*** | No frailty criteria measure (aged 70 years old or older, discharged home from the hospital ED, passing the abbreviated Mini-Mental State Exam, requiring assistance with at least one activity of daily living (OARS- ADL scale) or two instrumental activities of daily living (OARS-IADL scale), and having a probability of 40% or more of admission to hospital as defined by the Boult assessment tool) |
| ***Dalby et al., 2000*** | No frailty criteria measure (people 70 years of age or more) |
| ***Leung et al., 2004*** | No frailty criteria measure ( aged 65 and over; a recent history of repeated hospitalisations (ie, two or more episodes in the past 6 months); multiple problems (ie, two  or more chronic medical conditions that included hypertension, diabetes, chronic obstructive airway disease, stroke/CVA, heart failure, Parkinson’s disease, etc); home dwelling |
| ***Caplan et al., 2006*** | No frailty criteria measure (inpatients with a length of stay (LOS) exceeding 6 days, who were referred for geriatric rehabilitation) |
| ***Melis et al., 2008*** | No frailty criteria measure (lived in their own home or in a retirement home and were 70 years old or older and had one or more limitations in cognition, (instrumental)  activities of daily living, or mental well-being. |
| ***Courtney et al., 2009*** | No frailty criteria measure (chronic illness, etc.) (aged 65 and older, admitted with a medical diagnosis, and at least one risk factor for readmission (aged _75, multiple admissions in previous 6 months, multiple comorbidities, lived alone, lacked social support, poor self-rated health, moderate to severe functional impairment, and history of depression). |
| ***Senior et al., 2014*** | No frailty criteria measure, but considers its participants as older fragile adults |
| ***Sandberg et al., 2015*** | No frailty criteria measure (aged 65 years or older, be dependent in at least two ADL and have been admitted to hospital at least twice or had at least four visits in outpatient or primary care during the previous 12 months) |
| ***Thygesen et al., 2015*** | No frailty criteria measure. (patients aged 65 years or older discharged alive from the Department of Internal Medicine of Holbæk University Hospital, Denmark and living in one of the three surrounding municipalities and who had dementia or two of the following conditions: two or more hospital admissions within the 12 months before the index admission, loss of physical functioning, treatment of two or more concurrent medical or surgical conditions, mental disorder, six or more prescription medications, symptoms of cognitive disturbance, substance abuse problem, disadvantaged social network, or need for increasing home care following the index admission). |
| ***Bleijenberg et al., 2016*** | To measure risk of frailty, a frailty index (FI) was constructed based the cumulative deficit model using routine care data regarding 50 potential health deficits. |
| ***Ekdahl et al., 2016*** | No frailty criteria measure (Community-dwelling individuals aged 75 years who had received inpatient hospital care 3 or more times in the past 12 months and had 3 or more concomitant medical diagnoses) |
| ***Sahota et al., 2017*** | Frail older people |
| ***Parsons et al., 2017*** | No frailty criteria measure, but considers its participants as older fragile adults |
| ***Mogensen et al., 2018*** | No frailty criteria measure, but considers its participants as older fragile adults |
| ***Spoorenberg et al., 2018*** | Level of frailty was measured using the Groningen Frailty Indicator [GFI] |
| ***Berntsen et al., 2019*** | No frailty criteria measure, but considers its participants as older fragile adults |
| ***Lembeck et al., 2019*** | Frailty concept based on accumulated disabilities (Frailty Index ??) considered patients frail when they had a number of disabilities or known risks for readmission |
| ***Lindhardt et al., 2019*** | No frailty criteria measure, but considers its participants as older fragile adults |
| ***Suikkanen, et al., 2021*** | Based on Frail phenotype of Fried |
| ***Gilbert et al., 2021*** | No frailty criteria measure, but considers its participants as older fragile adults |
| ***Jepma et al, 2021*** | Screening instrument for frail older people of the Dutch Safety Management System (DSMS). Four geriatric conditions (limitation in activities of daily living [ADL], falls, malnutrition and delirium) are part of this frailty tool, and the DSMS-score ranges between 0 and 4 |
| ***Liang et al, 2021*** | No frailty criteria measure, but considers its participants as older fragile adults |
| ***Schapira et al., 2021*** | Frailty criteria measure (Frailty Index) |
| ***Shepperd et al, 2021*** | No frailty criteria measure, but considers its participants as older fragile adults |
| ***Jacobsohn et al, 2022*** | No frailty criteria measure, but considers its participants as older fragile adults |
| ***Casas-Herrero et al., 2022*** | Based on Frail phenotype of Fried |

**Table S6.** Inclusion criteria, main health participants problems and intervention (experimental and control groups)

| **Study** | **Inclusion criteria and Health problems participants reported** | **Intervention** |
| --- | --- | --- |
| ***Bernabei et al., 1998*** | **Inclusion criteria/health problems:** All people aged 65 and over who were recipients of home health services or home assistance programmes (n = 224). Usually, patients were receiving these services because of multiple geriatric conditions (for example, dementia, immobility, incontinence, and stroke deficits), but the evaluation preceding care planning was not based on a comprehensive geriatric assessment. | **Experimental:** case management and care planning by the community geriatric evaluation unit and general practitioners.  **Control:** received primary and community care with the conventional and fragmented organisation of services—that is, general practitioner's regular ambulatory and home visits, nursing and social services, home aids, and meals on wheels. |
| ***Berntsen et al., 2019*** | **Inclusion criteria**: aged> 60 years, referred to a Patient-Centred Team - PACT-team to be reviewed for eligibility to receive PACT care and the patient provided an oral consent. | **Experimental:** Patient-Centred Team intervention across primary and secondary care. The PACT team collaborate with the patient to make and deliver a person-centred, integrated and proactive multi-morbidity care-plan  **Control:** Usual care (evidence-based care for the cause of emergency admission to hospital). Depending on the patient’s condition, care for other diagnoses is either achieved through referrals to the appropriate in-house specialist service, or through a recommendation for follow-up in the discharge letter directed at the GP. Care-coordination and integration at discharge is achieved through standard electronic communication and discharge routines designed in collaboration by the hospital and municipal care organizations. |
| ***Bleijenberg et al., 2016*** | **Inclusion criteria:** potentially frail individuals aged 60 and older were  identified by screening their electronic medical records (EMRs) using predefined screening criteria. Individuals aged 60 and older were considered at risk if they were at risk for frailty, were exposed to polypharmacy, or had not had a visit with their general practitioner (GP) for 3 years or more (consultation gap). A combination of the three criteria was used to exclude  older adults who did not have any health deficits.  **Health problems:** NA | **Intervention A:** Frailty Screening Followed by Routine Care from a General Practitioner  **Intervention B:** Frailty Screening Followed by Personalized Nurse-Led Care  **Control:** care as usual, which was defined as the continuation of daily care practice without the implementation of either intervention. |
| ***Caplan et al., 2006*** | **Inclusion criteria:** inpatients with a length of stay (LOS) exceeding 6 days, who were referred for geriatric rehabilitation, were eligible for inclusion in our trial on condition that they required and were suitable for rehabilitation; that is they were expected to return home and live reasonable independently after rehabilitation and lived in the local area of the hospital, but not in a nursing home.  **Health problems:** ischemic heart diseases, diabetes, and dementia | **Experimental:** Home rehabilitation was provided by a hospital-based multidisciplinary outreach service which also provides home rehabilitation for orthogeriatric and an acute admission substitution Hospital in the Home service. Patients deteriorated once transferred home, certain conditions could be treated at home, such as infections requiring intravenous antibiotics, provided the patient was not hypotensive or hypoxic.  **Control:** Hospital rehabilitation in geriatric rehabilitation ward when a bed was available, and their acute illness was settling. |
| ***Casas-Herrero et al., 2022*** | **Inclusion criteria**: age >75 years, Barthel Index score ≥60 (scale, 0[severe functional dependence] to 100 [functional independence]), being able to communicate and ambulate (with/ without assistance), MCI or mild dementia according to Diagnostic and Statistical Manual of Mental Disorders (DSM)V criteria, Global Deterioration Scale (GDS)-4 (Reisberg classification), pre-frail and frail status according to the Fried criteria,18 and having someone to help supervise the exercises.  **Health problems**: dementia, depression, nutritional impairment, cognitive impairment, disability, mobility impairment. | **Experimental:** In addition to habitual outpatient care, the intervention group received the recently developed Vivifrail multicomponent exercise programme which included resistance, balance, flexibility (3 days/week), and gait-retraining exercises (5 days/week) and was performed for three consecutive months.  **Control:** usual-care group were instructed to continue with their normal ADLs and received habitual outpatient clinical care, including medical treatments and physical rehabilitation when needed. |
| ***Courtney et al., 2009*** | **Inclusion criteria**: aged 65 and older, admitted  with a medical diagnosis, and at least one risk factor for readmission (aged 75, multiple admissions in previous 6 months, multiple comorbidities, lived alone, lacked social support, poor self-rated health, moderate to severe functional impairment, and history of depression  **Health problems**: cardiac disease, respiratory disease, gastrointestinal problems, and falls. The median number of comorbidities was five including cardiac, orthopedic, respiratory, gastrointestinal and endocrine diseases. | **Experimental:** Comprehensive nursing and physiotherapy assessment and individualized program of exercise strategies and nurse-conducted home visit and telephone follow-up commencing in the hospital and continuing for 24 weeks after discharge.  **Control:** routine care, discharge planning, and rehabilitation advice normally provided. |
| ***Dalby et al., 2000*** | **Inclusion criteria**: 70 years of age or more on the roster of 2 physicians affiliated with an HSO in Stoney Creek. Respondents were considered eligible if they reported functional impairment, or admission to hospital or bereavement in the previous 6 months.  **Health problems**: The most common problems were urinary tract infections (27.4%), gastroenteritis (27.4%), chest infections (24.7%), depression (15.1%) viral illnesses (15.1%), insomnia (6.8%) and  hearing impairment (6.8%). | **Experimental:** The nurse reviewed each person’s medical record and completed a comprehensive assessment addressing physical, cognitive, emotional and social function, medication use, and the safety and suitability of the home environment. A care plan was developed together with the primary care physician, the patient, the family, caregivers and other health care professionals. Follow-up visits and phone calls were conducted as needed over the course of the 14-month trial to provide vaccinations, monitor, promote health and provide psychosocial support. The nurse served as a case manager by integrating community services and agencies, such as Home Care, into the participants’ care plan.  **Control:** Usual care |
| ***Ekdahl et al., 2016*** | **Inclusion criteria and health problems**: community dwelling and aged75 years or older, had received inpatient hospital care 3 or more times in the previous 12 months, and had 3 or more concomitant medical diagnoses (hearing and visual impairments, neosplams, certain infectious and parasitic diseases, diseases of the blood and immune mechanism, endocrine, nutritional and metabolic diseases, mental and behavioural disorders, diseases of the nervous system, diseases of the circulatory system, diseases of the respiratory system, diseases of digestive system, diseases of the musculoskeletal system, and symptoms, signs, and abnormal clinical/laboratory findings) | **Experimental:** care based on comprehensive geriatric assessment (CGA) as a complement to usual care in an outpatient setting  **Control:** usual care (usual social and health care, delivered at home, in primary care centers, and in the hospital) |
| ***Gagnon et al., 1999*** | **Inclusion criteria:** Aged 70 years old or older, discharged home from the hospital ED, living in the catchment areas of the CBte des Neiges or Rent Cassin community health centers, speaking English or French, passing the abbreviated Mini-Mental State Exam, requiring assistance with at least one activity of daily living (OARS- ADL scale) or two instrumental activities of daily living (OARS-IADL scale), and having a probability of 40% or more of admission to hospital as defined by the Boult assessment tool12 (measuring self-rated health, admission to hospital in previous 12 months, physician or clinic visit in previous 12 months, ever history of cardiac disease, and current availability of caregiver).  **Health problems participants:** NA | **Experimental:** Nurse case management, which consisted of coordination and provision of healthcare services by nurses, both in and out of hospital, for a 10- month period.  **Control:** Usual care, which varied by healthcare provider and community health center. |
| ***Gilbert et al., 2021*** | **Inclusion criteria and main health problems**: aged 75 years or older hospitalized in a participating acute care geriatric unit for at least 48 hours and returning home after hospitalization (ie, without transfer to step-down community hospital or rehabilitation unit). Patients were included if they were deemed at risk of hospital readmission after returning home based on the presence of at least 2 of the following criteria derived from the Triage Risk Screening Tool and French guidelines (Dependencies in daily living, previous admissions (1 unscheduled hospital admission during the 3 previous months, or 2 or more unscheduled hospital admissions during the previous year), geriatric syndrome (2 or more falls during the previous year, undernutrition, diagnosed major cognitive disorder, or depression), one or o more chronic diseases with high risk of acute decompensation or hospital readmission ((eg, chronic heart failure, chronic respiratory failure), polypharmacy (defined as daily intake of 5 or more drugs), Unfavorable social situation (social isolation, unreliable helper). | **Experimental:** The aim was to bridge the patients’ pathways at 3 steps: (1) during hospitalization (support with discharge planning, communication with community services, and anticipation of needs after discharge); (2) on the day of discharge (making sure all elements of the care plan are made operational with regard to prescriptions or  package of care (POC; ie, the combination of professional helpers and services put together to meet the person’s assessed needs), providing a handover sheet with summary of hospitalization and care plan and providing a contact phone number in case of need); (3) and follow-up after discharge. This follow-up was of 1 month comprising 2 home visits (at 48-72 hours after discharge and during third week) and a minimum of 2 telephone calls during second and fourth week from discharge.  **Control**: usual care plan of each participating hospital. Communication of information to the primary care providers was left to the discretion of the medical teams, with no additional follow-up after discharge. |
| ***Jacobsohn et al, 2022*** | **Inclusion criteria:** at least 60 years of age; resided in either Dane County, Wisconsin, or Monroe County, New York; had a primary care provider affiliated with either health system; had a working telephone; and were discharged from the ED to a community residence within 24 h of arrival.  **Health problems:** NA | **Experimental:** Treatment group participants received the Care Transitions Intervention (CTI), adapted for use following ED visits,26 delivered by community paramedic coaches certified to deliver the CTI by intervention developers.27 The intervention consisted of a home visit 24 to 72 h post-discharge and up to three coaching phone calls within 28 days.  **Control:** Usual care |
| ***Jepma et al, 2021*** | **Inclusion criteria:** Cardiac patients of ≥70 years, admitted to the departments  of cardiology or cardiothoracic surgery and admitted ≥48 h were eligible if they were at high risk of functional loss according to the screening instrument for frail older people of theDutch Safety Management System (DSMS). Four  geriatric conditions (limitation in activities of daily living [ADL], falls, malnutrition and delirium) are part of this frailty tool, and the DSMS-score ranges between 0 and 4. Patients were considered at high risk with a DSMS-score≥2 in patients aged 70–79 years or DSMSscore≥ 1 in patients aged ≥80 years [21]. Regardless of the DSMS-score, we also included patients with an unplanned hospital admission in the prior 6 months as this is associated with increased risk for adverse events  **Health problems:** Heart failure (58%), unplanned hospital admission in the previous 6 months, 56% were at risk of delirium, 47% had fallen in the 6months prior to admission, 39% had ADL-limitations and 33% had malnutrition | **Experimental:** The Cardiac Care Bridge (CCB) programme was performed in three phases: the clinical, discharge and post-clinical phase. The intervention consisted of three care components: (i) case management, (ii) disease management and (iii) home-based Cardiac Rehabilitation (CR).  **Control:** Usual care including consultation by other disciplines during hospitalisation, outpatient visits to the cardiologist and cardiac nurse specialist and centre-based CR if indicated. In addition, standard care was provided by the family physician |
| ***Lembeck et al., 2019*** | **Inclusion criteria**: age 65 or older (during the first 13 months of the study the age limit used was age 78 or older, but due to few participants we extended the age spectrum), discharge with any diagnosis from the Medical, Geriatric, Emergency, Surgical or Orthopedic departments. Minimum 3 out of 9 medical and social conditions had to be met: cognitive and psychiatric disorders, drug or alcohol abuse, lack of social network (recent loss of spouse, ill spouse, living alone), low level of functioning, multiple medications (6 or more drugs), hospital contacts within 6 months before index hospitalization, falls history, suspicion of housing conditions that hamper the patient in his daily activities. The inclusion criteria are not classical frailty criteria but were developed in a local group of practitioners including geriatric specialists and nurses from hospital as well as from the municipalities.  **Health problems**: Stroke, Dehydration, Constipation, Pneumonia, Urinary tract infection, Chronic obstructive, lung disease, Ischemic heart disease, Gastroenteritis, Fragility fracture, Iron deficiency, anemia, Arthritis, Social causes, Pressure ulcers | **Experimental:** reviewed the patient’s hospitalization and discharge plans on the day before discharge. On the day of discharge, the project nurse accompanied the patient to the patient’s home where they met the municipal nurse. Together with the patient and in the patient’s own surroundings the two nurses performed a structured assessment reviewing cognitive skills, medicine, nutrition, home environment, mobility, level of functioning and future appointments in the health care sector. The assessment was followed by an intervention based on the findings in the assessment. If for instance the nurses found cognitive dysfunction, the patient would be referred to skilled nursing specialists on dementia or if there were questions concerning use of drugs the patients GP would be consulted. Another intervention could be minor adjustments in the home environment.  **Control:** usual care implied communication between hospital and municipality and general practitioner by means of electronic communication concerning hospitalization, advice on medications, home care, and rehabilitation in the municipality. |
| ***Leung et al., 2004*** | **Inclusion criteria/health problems:** aged 65 and over; a recent history of repeated hospitalisations (ie, two or more episodes in the past 6 months); multiple problems (ie, two or more chronic medical conditions that included  hypertension, diabetes, chronic obstructive airway disease, stroke/CVA, heart failure, Parkinson’s disease, etc); home-dwelling; and agreement to participate in the project for a period of 12 months. | **Experimental:** case management services, with the case manager coordinating all  services received. The scope of services included: regular monitoring of subjects’ health status so that preventive and corrective interventions could be delivered proactively; availability for phone assistance to subjects daily from 8am to 9pm; home visits, if needed; prescribing of community-based supportive services, including community nursing services; and access to the case geriatrician by the case manager for medical support which included telephone consultation, assessment of subjects in the outpatient department, and admission of subjects to the hospital for further investigation and treatment.  **Control:** usual service of regular medical follow-up through the hospital service  system |
| ***Liang et al, 2021*** | **Inclusion criteria:** inpatients from a 400- bed regional hospital in Taiwan, age > 65 years, high risk for readmission with a length of stay, acuity of admission,  comorbidity, and visits to ED (LACE) index of ≥7, and willingness to participate.  **Health problems:** NA | **Experimental:** each participant in the IG were provided wireless transmission devices, including a one-touch smartphone, blood pressure (BP) monitor, medication dispenser, and a necklace emergency call button. Participants with diabetes were also given a  glucometer to measure their blood sugar. The BP monitor measured systolic BP, diastolic BP, heart rate, and presence of arrhythmia (yes or no). The one-touch smartphone provided to the patient or caregiver enabled communication with a nurse-led 24-hr call center, and the smartphone was used to set up twice daily reminders to check vital signs (BP, heart rate, arrhythmia, and blood sugar biological parameters) and take medication. All wireless devices connected to the onetouch smartphone then were automatically transmitted to the 24-hr call center and healthcare record system  via Bluetooth to assist in physician assessment. The IG program offered continuous telemonitoring through wireless transmission devices and home visits. The 24-hr call center was staffed by eight senior nurses (including one home visit nurse) on two 12-hr shifts a day, with two or three senior nurses per shift. The  nurses composed personalized alerts set for each patient, with established threshold values based on American College of Cardiology or American Heart Association  guideline, which were essential for monitoring the patient’s condition and detecting unusual changes. Each participant checked biological parameters twice a day (at 8:00 a.m. and 6:00 p.m.). Their family or caregivers assisted in connecting the BP cuff for checking vital signs and in measuring their blood sugar. If the call center did not receive valid vital signs on time, nurses called the patients or caregivers through the one-touch smartphone to remind them. Any unusual parameters triggered an alert, and depending on the patient’s condition, the nurses sought an initial assessment of symptoms and rechecking of abnormal parameters. On the basis of this assessment,  the nurses made the decision whether to consider the alert an emergency event and whether to transfer the patient to emergency services or specialist services (outpatient). Additionally, nurses provided patients and caregivers with health education, nutrition and medication consultation, medication reminders, appointment  scheduling, or reminders for follow-ups with a physician and emergency medical service. To ensure integrated program compliance and to meet patients’ medical needs, tele-homecare nurses also conducted home visits (content of care included assessment, patients’ education, nutrition and medication consultation, and medication reminders) on the discharge day (T0), 3 months after discharge (T3), and 6 months after discharge (T6) and additional visits depending on the patient’s individual needs.  **Control:** team members included four senior nurses and physicians. Before discharge, patients received discharge planning to promote their home care ability (content of care included assessment, nutrition and medication consultation, and medication reminders). The home visits were made by the nurses at the patients’ homes for provided home care (content of care included assessment, checking vital signs, patient  education, nutrition and medication consultation, and medication reminders) and data collection at 3 months after discharge and 6 months after discharge. |
| ***Lindhardt et al., 2019*** | **Inclusion criteria**: 65 years or older discharged to their own home less than 72 hours after admission.  **Health problems**: NA | **Intervention A:** patients were informed of health problems and self-care interventions.  **Intervention B:** a motivational conversation targeting activities of daily living with a home care nurse and a home visit with follow-up)  **Control:** usual care based on a final round was held with the physician and a staff nurse, who informed the patient of any plans for further treatment and handed out prescriptions for new drugs if appropriate. |
| ***Melis et al., 2008*** | **Inclusion criteria/health problems:** lived in their own home or in a retirement home and were 70 years old or older and had one or more limitations in cognition, (instrumental) activities of daily living, or mental well-being. | **Experimental:** problem-based selection procedure performed by the primary care physician, rather than population screening to identify patients eligible for participation. The problems targeted concerned cognition, nutrition, behavior, mood, or mobility, and had to require nursing assessment, coordination of care, therapeutic monitoring, or case management (Table 1). Within 2 weeks after referral, a geriatric specialist nurse visited the patient at home. Up to six visits for additional geriatric evaluation and management were planned within the next 3 months. Starting off from  a wide multidimensional assessment, the intervention team developed an individualized, integrated treatment plan for each patient. The nurse conducted the main part of the intervention. The primary care physicians continued their usual medical care. Moreover, they made referrals, medication changes, and other interventions as agreed upon during interdisciplinary consultations with the nurse and geriatrician on individual cases. The primary care physician continued to be primarily responsible for the care of the patient and made the final decisions. It was developed  guidelines for each of the five presenting health problems to structure activities, without losing the flexibility of tailoring the individual interventions.  **Control:** usual care group received unrestricted care |
| ***Mogensen et al., 2018*** | **Inclusion criteria and health problems**: ≥ 65 years old patients with an acute medical condition that required acute hospital in-patient care (acute exacerbation of Chronical Obstructive Pulmonary Disease, Dehydration, Delirium, Fever, Pneumonia Urinary infection diseases, other diseases) | **Experimental:** Hospital at home model based on be cared by the patient´s own general practitioner  **Control:** hospital specialist-based model (based on hospital intern specialist care) |
| ***Parsons et al., 2017*** | **Inclusion criteria**: older people (age >65 years) assessed at high risk of permanent institutional care (dementia, with associated behavioral problems; incontinence; career stress; repeated falls; and frailty)  **Health problems:** visual and hearing problems, memory problems, communication problems, functional ability impairment, cognitive impairment, depression | **Experimental:** Community Flexible Integrated Responsive Support Team (FIRST), a new service delivery model known in New Zealand as restorative home support which improve the quality of home care and maximize the ability of frail older people to continue living in their own homes for as long as possible.  **Control**: usual care |
| ***Sahota et al., 2017*** | **Inclusion criteria:** frail older people aged 70 years and older admitted to hospital as an acute medical emergency.  **Health problems:** NA | **Experimental:** Community In-Reach and Care Transition (CIRACT service)  **Control:** traditional hospital-based rehabilitation THB-Rehab service (standard care) |
| ***Sandberg et al., 2015*** | **Inclusion criteria:** be aged 65 years or older and lived in an ordinary home, be dependent in at least two ADL and have been admitted to hospital at least twice or had at least four visits in outpatient or primary care during the previous 12 months.  **Health problems:** cardiac diseases (atrial fibrillation, flutter, heart failure, essential (primary) hypertension, functional disability, cognitive impairment | **Experimental:** four different parts: ‘‘traditional’’ case management (assessment, care coordination, home visits, telephone calls, advocacy), general information (about the healthcare system, social activities, nutrition, exercise etc.), specific information (related to the participant’s specific health status, individual needs and medication) and safety (the availability of the nurse or physiotherapist by cell phone during working hours)  **Control:** usual care |
| ***Schapira et al., 2021*** | **Inclusion criteria**: adults aged 75 years and older who had an unplanned hospital admission to the internal medicine service and fulfilled the definition of frailty according to the frailty index (FRAIL scale)  **Health problems:** Respiratory infection, Delirium, Cardiovascular diseases, Falls, Hip fracture, Urinary infection, Gastrointestinal disease | **Experimental:** in addition to usual care, a geriatric co-management team performed a comprehensive geriatric assessment during hospitalization, provided tailored recommendations to minimize geriatric syndromes and planned transition of care. A health and social care counselor oversaw continuity of care in patients’ homes after discharge.  **Control:** Usual care treatment arm: all procedures performed during hospitalization  were overseen by a senior internal medicine specialist and complied with pre-defined protocols. Patients had access to specialist care if needed, as well as hospital-at-home or home-based primary care services after discharge |
| ***Senior et al., 2014*** | **Inclusion criteria:** aged ≥65 years (≥55 years for Māori) at high risk of institutionalization  **Health problems**: required help with everyday activities, indoor mobility problems, memory, or communication problems, falls within 6 months | **Experimental:** Promoting Independence Programmes provides case-managed restorative care delivered within both residential care and at home by a multi-disciplinary team, based on ‘home treatment teams’  **Control:** usual care based on community services or permanent placement in residential care |
| ***Shepperd et al, 2021*** | **Inclusion criteria:** 65 years or older; willing and able to give informed consent, or if lacking capacity to consent they had a personal consultee, healthcare proxy or an Independent Mental Capacity Advocate; had been referred to a geriatrician-led admission avoidance hospital at home service with Comprehensive Geriatric Assessment (CGA) and would otherwise require hospital admission; and English speaking**.**  **Health problems:** 72.3% had some cognitive impairment, 6.8% delirium. Health problems and diagnoses that are typically associated with hospital admission for this population (Infection, cardiac disease,…), over 60% reported moderate or severe problems with mobility and 38% reported difficulties with activities of daily living. | **Experimental:** Admission avoidance Comprehensive Geriatric Assessment Hospital at Home (CGAHAH) is equivalent to bed based hospital care for older people with frailty who are medically unwell and physiologically stable, it is a rapid response service that assesses a patient within one to two hours of referral and is provided for a limited time. Similar to bed based hospital care for this population, the acute medical problem is treated in the context of physical, psychological, social and functional issues to optimise recovery and that require multidisciplinary care. Radiological investigations are prioritised as if in hospital, with CGAHAH organising transport. During the design of the trial we established four core components (18) that had to be present for the CGAHAH intervention to provide an alternative to hospitalization. These were i) geriatrician-led admission avoidance HAH; ii) a multi-disciplinary team; iii) healthcare guided by the principles of CGA, that included virtual rounds; and iv) direct access acute hospital based healthcare, such as diagnostics and transfer to hospital.  **Control:** Hospital care delivered (80% patients received geriatrician led care with CGA and 20% general medical hospital care without input from an attending geriatrician). All received multi-disciplinary care. |
| ***Spoorenberg et al., 2018*** | **Inclusion criteria:** all older adults aged 75 and over who were registered with one of the participating general practitioners (GPs) and were living at home or in a home for the elderly were invited to participate.  **Health problems:** NA | **Experimental:** Embrace (in Dutch: SamenOud [ageing together]) is a person-centred and integrated care service for community-living older adults. The intensity, focus, and individual or group approach of the care and support depended on the participant's risk profile. We invited all participants to follow a self-management support and prevention program focusing on staying healthy and independent for as long as possible.  The program included regular Embrace community meetings, in which selfmanagement abilities were encouraged and during which local healthcare and welfare organisations provided information on health maintenance, physical and social activities, and dietary recommendations. In addition, frail people and those with complex care needs received individual support from a case manager. They jointly developed an individual care and support plan targeting all health-related problems, which had to be agreed upon by the Elderly Care Team before implementation. The case managers monitored changes in the medical, psychosocial, or living situation, and navigated the plan's delivery. The Elderly Care Team discussed and evaluated the participants' health status and social situation in monthly meetings. If necessary,  they took proactive steps in dialogue with participants to prevent deterioration. People  with a `Robust' profile were encouraged to contact the team in the event of changes in their health or living situation.  **Control:** care as usual as provided by their GPs and local health and community  organisations. Municipalities are in charge of social care, disease prevention and health promotion. Once a health problem is found, patients enter the health care system in most cases with a visit to their GP. In the Netherlands, GPs are family physicians who usually have a long term relationship with their patients. They act as gatekeepers for specialised services in the Dutch healthcare system: patients need a referral to enter specialised medical care. |
| ***Suikkanen, et al., 2021*** | **Inclusion criteria:** to score at least 1 point in the FRAIL questionnaire and fulfill at least 1 of the frailty phenotype criteria. Two of the phenotype criteria were slightly modified. To define “low physical activity,” we used 30 minutes per week as a cutoff value. For the slowness criterion, we used a common gait speed cutoff value of 0.46 m/s for both genders, which was based on the lowest quartile in the Short Physical Performance Battery.24,25 Participants were classified as pre-frail if they met 1 to 2 phenotype criteria and frail if they met 3 to 5. Other eligibility criteria were as follows: age 65 years, home-dwelling (with or without homecare services), able to walk with or without aid when indoors, a Mini-Mental State Examination (MMSE) score of 17, and no severe illnesses that prevented them taking part in exercise training.  **Health problems**: Cardiovascular diseases, Hypertension, Stroke or TIA, Diabetes, Musculoskeletal diseases, COPD or asthma, Dementia | **Experimental:** physical exercise group performed physiotherapist-supervised home-based physical exercises for 60 minutes, twice a week over 12 months.  **Control:** continued to live their lives as usual, without restrictions. |
| ***Thygesen et al., 2015*** | **Inclusion criteria and health problems**: aged 65 years or older discharged alive from the Department of Internal Medicine of Holbæk University Hospital, Denmark who had dementia or two of the following conditions: two or more hospital admissions within the 12 months before the index admission, loss of physical functioning, treatment of two or more concurrent medical or surgical conditions, mental disorder, six or more prescription medications, symptoms of cognitive disturbance, substance abuse problem, disadvantaged social network, or need for increasing home care following the index admission. | **Experimental:** home visits with a general practitioner and municipal nurse within seven days of discharge focusing on medication, rehabilitation plan, functional level, and need for further health care initiatives. The visit was concluded by planning one or two further visits.  **Control:** usual care (including a discharge letter from the hospital to the general practitioner but not including coordinated home visits from the municipal nurse and the primary general practitioner: |

**Table S7.** Subgroup analyses based on type of intervention, type of control, and evaluation of the risk of bias

|  | Type of intervention | | | | | Type of control | | | | | Evaluation of risk of bias | | | | | |
| --- | --- | --- | --- | --- | --- | --- | --- | --- | --- | --- | --- | --- | --- | --- | --- | --- |
|  | #studies |  | OR | LL | UL |  | #studies | OR | LL | UL |  | | #studies | OR | LL | UL |
| EMERGENCY VISITS | 6 | CM | 0.85 | 0.76 | 0.94 | Ambulatory and home visits | 2 | 0.45 | 0.27 | 0.76 | Low | | - | - | - | - |
|  | 3 | MIXED | 0.90 | 0.82 | 0.99 | Usual care | 8 | 0.91 | 0.76 | 1.09 | Some concerns | | 8 | 0.88 | 0.69 | 1.13 |
|  | 1 | TC | 0.88 | 0.82 | 1.24 | Hospital rehabilitation | - | - | - | - | High | | 2 | 0.70 | 0.44 | 1.13 |
|  | - | HAH | - | - | - |  |  |  |  |  |  | |  |  |  |  |
| HOSPITALIZATIONS/DAYS | 3 | CM | 1.30 | 1.00 | 1.70 | Ambulatory and home visits | - | - | - | - | Low | | - | - | - | - |
|  | 2 | MIXED | 1.06 | 0.50 | 2.21 | Usual care | 5 | 1.29 | 1.04 | 1.59 | Some concerns | | 6 | 0.86 | 0.49 | 1.49 |
|  | 1 | TC | 0.76 | 0.47 | 1.25 | Hospital rehabilitation | 2 | 0.31 | 0.05 | 1.93 | High | | 1 | 0.76 | 0.47 | 1.25 |
|  | 1 | HAH | 0.12 | 0.05 | 0.26 |  |  |  |  |  |  | |  |  |  |  |
| HOSPITALIZATIONS/NUMBER | 5 | CM | 0.91 | 0.66 | 1.25 | Ambulatory and home visits | 1 | 1.13 | 0.65 | 1.98 | Low | |  | - | - | - |
|  | 6 | MIXED | 0.96 | 0.74 | 1.25 | Usual care | 10 | 0.92 | 0.73 | 1.15 | Some concerns | | 10 | 1.02 | 0.87 | 1.19 |
|  | 2 | TC | 1.10 | 0.79 | 1.54 | Hospital rehabilitation | 2 | 1.06 | 0.84 | 1.33 | High | | 3 | 0.83 | 0.53 | 1.28 |
|  | - | HAH | - | - | - |  |  |  |  |  |  | |  |  |  |  |
| MORTALITY | 7 | CM | 1.11 | 0.66 | 1.87 | Ambulatory and home visits | 2 | 0.61 | 0.25 | 1.50 | Low | | 3 | 0.86 | 0.55 | 1.37 |
|  | 13 | MIXED | 0.80 | 0.65 | 0.99 | Usual care | 17 | 0.80 | 0.65 | 0.97 | Some concerns | | 15 | 0.86 | 0.70 | 1.05 |
|  | - | TC | - | - | - | Hospital rehabilitation | 3 | 1.20 | 0.90 | 1.60 | High | | 2 | 1.08 | 0.85 | 1.38 |
|  | 2 | HAH | 1.14 | 0.46 | 2.78 |  |  |  |  |  |  | |  |  |  |  |
|  | Type of intervention | | | | | Type of control | | | | | Evaluation of risk of bias | | | | | |
|  |  |  | ES | LL | UL |  |  | ES | LL | UL |  | |  | ES | LL | UL |
| QUALITY OF LIFE | 2 | CM | 0.07 | -0.12 | 0.26 | Ambulatory and home visits | 1 | 0.35 | 0.04 | 0.66 | Low | | 1 | 0.01 | -0.28 | 0.30 |
|  | 7 | MIXED | 0.10 | -0.02 | 0.21 | Usual care | 3 | 0.33 | -0.11 | 0.77 | Some concerns | | 5 | 0.21 | -0.02 | 0.45 |
|  | - | TC | - | - | - | Hospital rehabilitation | 2 | -0.03 | -0.02 | 0.17 | High | | - | - | - | - |
|  | 1 | HAH | 0.00 | -0.34 | 0.34 |  |  |  |  |  |  | |  |  |  |  |
| OR: Odd Ratio; LL: Low limit; UL: Upper limit; ES: Effect Size | | | | | | | | | | | |  |  |  |  |  |

**Table S8.** Meta-regression according to duration of the intervention, mean age of the intervention group, and percentage of women in the intervention group

|  | Duration of intervention | | Mean age of participants | | % women included in studies | |
| --- | --- | --- | --- | --- | --- | --- |
|  | Coefficient | p | Coefficient | p | Coefficient | p |
| EMERGENCY VISITS | 0.004 | 0.876 | 0.008 | 0.769 | 0.008 | 0.420 |
| HOSPITALIZATIONS/DAYS | -0.005 | 0.804 | -0.042 | 0.712 | -0.008 | 0.802 |
| HOSPITALIZATIONS/NUMBER | -0.006 | 0.639 | -0.013 | 0.619 | -0.004 | 0.683 |
| MORTALITY | -0.007 | 0.392 | 0.018 | 0.602 | 0.003 | 0.788 |
| QUALITY OF LIFE | 0.003 | 0.957 | -0.087 | 0.137 | -0.001 | 0.956 |

**Table S9.** Sensitivity analyses

| **Emergency** | **OR** | **LL** | **UL** | |  |
| --- | --- | --- | --- | --- | --- |
| Bernabei., et al | -0.1096 | -0.1365 | 0.0172 | |  |
| Berntsen., et al | -0.1176 | -0.2714 | 0.0361 | |  |
| Bleijenberg., et al (A) | -0.1427 | -0.2909 | 0.0055 | |  |
| Bleijenberg., et al (B) | -0.1379 | -0.2923 | 0.0164 | |  |
| Dalby., et al | -0.1226 | -0.2561 | 0.0107 | |  |
| Gagnon., et al | -0.1571 | -0.2730 | -0.0413 | |  |
| Jacobsohn., et al | -0.1339 | -0.2744 | 0.0067 | |  |
| Leung., et al | -0.1067 | -0.2313 | 0.0180 | |  |
| Liang., et al | -0.1041 | -0.2234 | 0.0153 | |  |
| Sandberg., et al | -0.1334 | -0.2671 | 0.0003 | |  |
| Schapira., et al | -0.1002 | -0.2355 | 0.0350 | |  |
| Suikkanen., et al | -0.1494 | -0.2778 | -0.0210 | |  |
| **Hospitalization days** |  |  |  | |  |
| Caplan., et al | 1.0882 | 0.7925 | 1.3839 | |  |
| Dalby., et al | 0.8620 | 0.3041 | 1.4199 | |  |
| Ekdahl., et al | 1.0015 | 0.3887 | 1.6143 | |  |
| Gagnon., et al | 0.8864 | 0.3126 | 1.4602 | |  |
| Gilbert., et al | 0.8475 | 0.3218 | 1.3732 | |  |
| Sahota., et al | 0.9899 | 0.3401 | 1.6398 | |  |
| Sandberg., et al | 0.9353 | 0.3410 | 1.5297 | |  |
| **Hospitalization number** |  |  |  | |  |
| Berntsen., et al | -0.0728 | -0.1860 | 0.0404 | |  |
| Bleijenberg., et al (A) | -0.1093 | -0.2504 | 0.0319 | |  |
| Bleijenberg., et al (B) | -0.1024 | -0.2467 | 0.0418 | |  |
| Dalby., et al | -0.1216 | -0.2471 | 0.0038 | |  |
| Ekdahl., et al | -0.1048 | -0.2316 | 0.0219 | |  |
| Gagnon., et al | -0.1260 | -0.2540 | 0.0019 | |  |
| Jacobsohn., et al | -0.1162 | -0.2457 | 0.0134 | |  |
| Jepma., et al | -0.1240 | -0.2522 | 0.0042 | |  |
| Leung., et al | -0.1067 | -0.2345 | 0.0211 | |  |
| Liang., et al | -0.1183 | -0.2455 | 0.0088 | |  |
| Lindhardt., et al (A) | -0.0935 | -0.2205 | 0.0335 | |  |
| Lindhardt., et al (B) | -0.0891 | -0.2130 | 0.0349 | |  |
| Sahota., et al | -0.1232 | -0.2486 | 0.0021 | |  |
| Sandberg., et al | -0.1133 | -0.2415 | 0.0149 | |  |
| Schapira., et al | -0.0855 | -0.2049 | 0.0339 | |  |
| Shepperd., et al | -0.1207 | -0.2531 | 0.0116 | |  |
| Suikkanen., et al | -0.13623 | -0.2578 | -0.0146 | |  |
| **Mortality** |  |  |  |  |  |
| Bernabei., et al | -0.1311 | -0.2905 | 0.0283 | | |
| Berntsen., et al | -0.0783 | -0.2136 | 0.0570 | | |
| Bleijenberg., et al (A) | -0.1396 | -0.3011 | 0.0218 | | |
| Bleijenberg., et al (B) | -0.1348 | -0.2985 | 0.0290 | | |
| Caplan., et al | -0.1331 | -0.2908 | 0.0246 | | |
| Casas-Herrero., et al | -0.1297 | -0.2825 | 0.0230 | | |
| Courtney., et al | -0.1271 | -0.2833 | 0.0290 | | |
| Dalby., et al | -0.1414 | -0.2920 | 0.0091 | | |
| Ekdahl., et al | -0.0914 | -0.2342 | 0.0515 | | |
| Gagnon., et al | -0.1415 | -0.2982 | 0.0152 | | |
| Gilbert., et al | -0.1214 | -0.2761 | 0.0333 | | |
| Jepma., et al | -0.1394 | -0.3051 | 0.0263 | | |
| Lembeck., et al | -0.1476 | -0.3087 | 0.0134 | | |
| Leung., et al | -0.1369 | -0.2903 | 0.0165 | | |
| Liang., et al | -0.1099 | -0.2561 | 0.0362 | | |
| Lindhardt., et al (B) | -0.1358 | -0.2898 | 0.0183 | | |
| Lindhardt., et al(A) | -0.1297 | -0.2860 | 0.0266 | | |
| Melis., et al | -0.1305 | -0.2876 | 0.0266 | | |
| Mogensen., et al | -0.1326 | -0.2875 | 0.0223 | | |
| Parsons., et al | -0.1242 | -0.2823 | 0.0340 | | |
| Sandberg., et al | -0.1460 | -0.2906 | -0.0013 | | |
| Schapira., et al | -0.1141 | -0.2769 | 0.0487 | | |
| Senior., et al | -0.1223 | -0.2795 | 0.0348 | | |
| Shepperd., et al | -0.1711 | -0.3224 | -0.0197 | | |
| Spoorenberg., et al | -0.1275 | -0.2851 | 0.0301 | | |
| Suikkanen., et al | -0.1314 | -0.2912 | 0.0284 | | |
| Thygesen., et al | -0.1264 | -0.2892 | 0.0363 | | |

| **Quality of life / Health related quality of life** | | | |
| --- | --- | --- | --- |
|  | **ES** | **LL** | **UL** |
| Bleijenberg., et al (A) | 0.1017 | -0.01201 | 0.2154 |
| Bleijenberg., et al (B) | 0.1007 | -0.0172 | 0.2185 |
| Casas-Herrero., et al | 0.0857 | -0.0147 | 0.1861 |
| Courtney., et al | 0.0311 | -0.0184 | 0.0807 |
| Gilbert., et al | 0.0791 | -0.0285 | 0.1868 |
| Liang., et al | 0.0559 | -0.0348 | 0.1466 |
| Lindhart., et al (IG1) | 0.0796 | -0.0211 | 0.1803 |
| Lindhart., et al (IG2) | 0.0836 | -0.0175 | 0.1847 |
| Mogensen., et al | 0.0847 | -0.0143 | 0.1839 |
| Shepperd., et al | 0.1011 | -0.0060 | 0.2083 |

**Table S10.** Publication bias

|  | **slope** | **Standard error** | **p** |
| --- | --- | --- | --- |
| **Mortality** | 0.7358 | 0.3057 | 0.024 |
| **Emergency** | -0.0977 | 0.8952 | 0.915 |
| **Hospitalization days** | 4.3375 | 1.1190 | 0.012 |
| **Hospitalization number** | 1.0485 | 0.5958 | 0.099 |
| **Quality of life** | 1.8756 | 0.8589 | 0.061 |

| 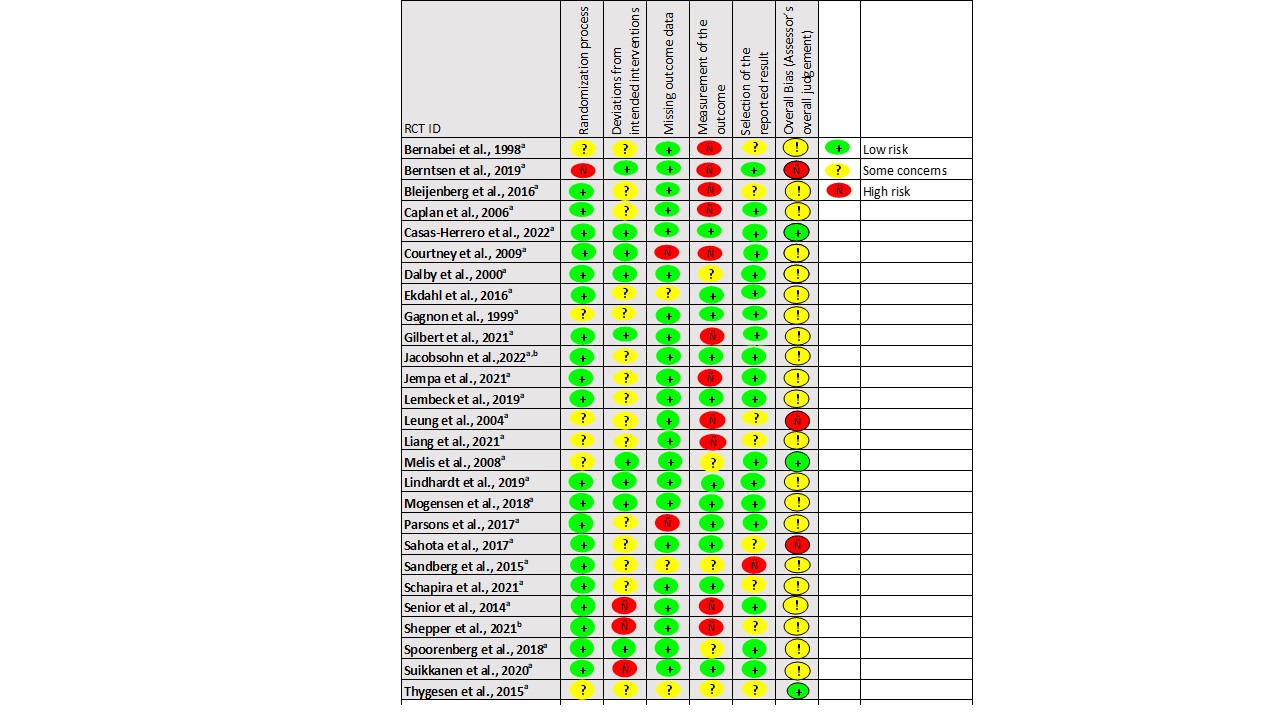 |
| --- |
| **Figure S1.** Risk of bias of studies included as assessed with the RoB2 tool.  ^a^ assignment to intervention (the 'intention-to-treat' effect); ^b^ adhering to intervention (the 'per-protocol' effect) |
|  |
